# Inequalities in Research on Food Environment Policies: An Evidence Map of Global Evidence from 2010-2020

**DOI:** 10.1016/j.advnut.2024.100306

**Published:** 2024-09-23

**Authors:** Laurence Blanchard, Stephanie Ray, Cherry Law, María Jesús Vega-Salas, Harry Rutter, Matt Egan, Mark Petticrew, Monique Potvin Kent, Claire Bennett, Patricia J Lucas, Cécile Knai

**Affiliations:** 1 Faculty of Public Health Policy, London School of Hygiene and Tropical Medicine, London, United Kingdom; 2 Department of Agri-Food Economics and Marketing, University of Reading, Reading, United Kingdom; 3 Centre for Exercise, Nutrition and Health Sciences, School for Policy Studies, University of Bristol, Bristol, United Kingdom; 4 Departamento de Nutrición y Dietética, Escuela de Ciencias de la Salud, Facultad de Medicina, Pontificia Universidad Católica de Chile, Santiago, Chile; 5 Department of Social & Policy Sciences, University of Bath, Bath, United Kingdom; 6 SPECTRUM Consortium, Edinburgh, United Kingdom; 7 School of Epidemiology and Public Health, University of Ottawa, Ottawa, Canada; 8 Public Health and Wellbeing team, Greenwich Borough, London, United Kingdom; 9 School for Policy Studies, University of Bristol, Bristol, United Kingdom; 10 Colectiv Tech, Bristol, United Kingdom

**Keywords:** policy, diet, equity, research, regulation, self-regulation, public-private partnership, evidence map, evidence synthesis

## Abstract

There has been increasing pressure to implement policies for promoting healthy food environments worldwide. We conducted an evidence map to critically explore the breadth and nature of primary research from 2010–2020 that evaluated the effectiveness, cost-effectiveness, development, and implementation of mandatory and voluntary food environment policies. Fourteen databases and 2 websites were searched for “real-world” evaluations of international, national, and state level policies promoting healthy food environments. We documented the policy and evaluation characteristics, including the World Cancer Research Fund International NOURISHING framework’s policy categories and 10 equity characteristics using the PROGRESS-Plus framework. Data were synthesized using descriptive statistics and visuals. We screened 27,958 records, of which 482 were included. Although these covered 70 countries, 81% of publications focused on only 12 countries (United States, United Kingdom, Australia, Canada, Mexico, Brazil, Chile, France, Spain, Denmark, New Zealand, and South Africa). Studies from these countries employed more robust quantitative methods and included most of the evaluations of policy development, implementation, and cost-effectiveness. Few publications reported on Africa (*n* = 12), Central and South Asia (*n* = 5), and the Middle East (*n* = 6) regions. Few also assessed public-private partnerships (PPPs, *n* = 31, 6%) compared to voluntary approaches by the private sector (*n* = 96, 20%), the public sector (*n* = 90, 19%), and mandatory approaches (*n* = 288, 60%). Most evaluations of PPPs reported on the same 2 partnerships. Only 50% of publications assessing policy effectiveness compared outcomes between population groups stratified by an equity characteristic, and this proportion has decreased over time. There are striking inequities in the origin, scope, and design of these studies, suggesting that research capacity and funding lies in the hands of a few expert teams worldwide. The small number of studies on PPPs questions the evidence base underlying the international push for PPPs to promote health. Policy evaluations should consider impacts on equity more consistently.

This study was registered at PROSPERO as CRD42020170963.

**Statement of Significance:**

This study indicates the presence of striking imbalances in the origin, quality, and scope of the evidence on food environment policies and a lack of focus on health equity. It also demonstrates the limited availability of evidence on public-private partnerships at the international, national, and state levels as a means to improve food environments.

## Introduction

Unhealthy diets are one of the risk factors responsible for the most deaths and disability-adjusted life years globally. According to the Global Burden of Disease Study 2017 [1], this is especially due to high sodium intake and low intakes of whole grains, fruit, vegetables, nuts, seeds, and ω-3 fatty acids. Furthermore, the daily consumption of sugar-sweetened beverages (SSBs), sodium, and both red and processed meat all exceed the “optimal level” of intake associated with a reduced risk from all causes of death [1]. Much of this is because ultraprocessed food and beverages, which tend to be high in fat, sugar, and/or salt, are generally inexpensive, easily accessible, highly promoted, and therefore highly consumed, compared to healthier alternatives [2]. This is often referred to as unhealthy “food environments.” According to the FAO, food environments include the availability and physical access to food (proximity), their economic access (affordability), and the marketing and information about products and health, as well as food quality and safety [2]. There are particularly startling inequalities in the distribution of food available, their price, quality, and promotion, as well in dietary and health outcomes, by socioeconomic status (SES), geographic location, ethnicity, and gender [2,3].

Given the major influence of the food environment on population diet, research suggests that upstream interventions tackling the latter are more promising than actions targeting individual behavior, despite their greater political implementation challenges. Upstream approaches are also generally more effective at reaching a large proportion of the population, including vulnerable groups [2,4,5]. These actions include policies promoting healthy food environments (named hereafter “food environment policies”). These policies aim to make healthier options more easily available and affordable and to limit the availability, affordability, and promotion of unhealthy ones. Since the 2010s, there has been increasing pressure to implement food environment policies; for example, by the United Nations (UN) High-level Meeting of the General Assembly on the Prevention and Control of Noncommunicable Diseases (2011) and its associated global action plan (2013), the framework of the Second International Conference on Nutrition (2014), and the UN Decade of Action on Nutrition (2016) [6,7]. A growing number of such policies are now being implemented worldwide and often relate to sugar, salt, and fat reduction, child food marketing, labeling, and infant formulas [[8], [9], [10]]. Many of these policies tend to be managed at the national or state/provincial level, depending on the division of power between levels of government.

Different regulatory approaches can be employed to implement such policies. These include *1*) mandatory approaches (public regulation with no involvement of private sector actors); *2*) voluntary approaches (whereby the public or private sector designs and monitors its own standards of conduct, including codes and self-regulation); and *3*) public-private partnerships (PPPs; defined hereafter as formalized agreements between ≥1 public and ≥1 private organization with a shared commitment to improve health [11,12]).

Evidence should play a key role in diet and food policymaking, and policy decisions should typically be based on data on effectiveness, cost-effectiveness, and implementability [13]. In the past decade, several evidence syntheses have explored the potential impact of various food environment policies using simulation and experimental studies (e.g., [[14], [15], [16]]). The nature of research on actual real-life food environment policies, either adopted or implemented, is unknown.

Evidence maps are a type of evidence synthesis that is increasingly being employed to examine the breadth, nature, and gaps in the evidence available in a given field using a systematic approach (e.g., [17,18]). They can be used, for example, to provide an overview of the types of interventions and outcomes assessed in studies covering a broad field in a single or multiple countries. Together with scoping reviews and evidence gap maps, they form what Campbell et al. [19] have described as the “Big Picture review family.” This contrasts with systematic reviews (with or without meta-analyses), which analyze the specific results of each of the studies included, often relating to intervention effectiveness, and which tend to focus on narrower research questions about fewer interventions. By including multiple interventions, outcomes, contexts, and types of evidence, the broad scope of evidence maps has the advantage of reflecting the nature of policy questions [20]. Descriptive by nature, evidence maps are often presented as a first step to informing research prioritization and agendas, guiding the design of in-depth syntheses of some of the included studies, and/or pointing to new primary research needs [19,20]. They can be complemented with an evidence gap map, which visually presents data in an interactive tabulation based on a theoretical framework. The aim of this study was to examine the breadth and nature of primary research evaluating the effectiveness, cost-effectiveness, development, and implementation of real-world mandatory, voluntary, and PPP food environment policies at the international, national, and state level between the years 2010–2020.

## Methods

This evidence map is part of a larger body of work investigating the effectiveness, cost-effectiveness, development, and implementation of mandatory and voluntary policies promoting healthy food environments and is registered on PROSPERO (CRD42020170963) [21]. As part of our larger project, the evidence map was also employed to identify studies to be analyzed in-depth in 5 additional evidence syntheses on different subareas identified in the map. A detailed protocol was submitted to our funder (National Institute for Health and Care Research). The evidence map was reported using the PRISMA extension for scoping reviews [22], where relevant (Supplementary Table 1).

### Eligibility criteria

This evidence map focused on real-world evaluations of food environment policies published between 2010 and 2020. No restriction on language or country was applied to include studies from around the world. “Real-world” referred to data collected either when a policy was adopted or implemented or as part of a state or national public consultation. Experiments, simulations and modeling studies were therefore ineligible unless based on real-world policy data. Policies had to target the general public, i.e., those focusing on specific groups such as athletes, the army, and employees were excluded. The first 3 categories of food environment by the FAO, i.e., proximity (e.g., school food standards), affordability (e.g., taxes), and marketing/information (e.g., advertising control, labeling, and displays in shops) were included with some exceptions. Food quality and safety were excluded both as a topic and an outcome, as well as policies not targeting ordinary food, e.g., “natural” products, supplements, alcohol, and sweeteners. To keep the size of the evidence map manageable, the following topics were also excluded: breastfeeding, health and nutrition claims, food fortification, international trade, taxes not specific to food, food security, undernutrition, and double/triple burden of malnutrition, as well as agriculture, farming, and sustainability as a primary focus rather healthy diet.

Both the policies and the evaluations had to be conducted at the international, national, or state level. However, assuming that the characteristics of products offered in supermarket chains or advertised on major television channels are similar across a region, audits of food products, shops, and television advertisements could be conducted at any level of government except in local independent premises or channels. All outcomes relating to the effectiveness, cost-effectiveness, and factors affecting the development or implementation of a policy were considered. Views of the general public outside public consultations as well as policy inventories and benchmarks were excluded. Protocols, working papers, thesis, and preprints were excluded. Given our focus on regulatory approaches (i.e., mandatory and voluntary actions—see note below regarding PPPs), studies of multiple policies with unclear approaches or that did not consider these in their analysis were excluded. We did not specifically search for grey literature, but those retrieved were screened for eligibility. An extended list of excluded topics and the eligibility criteria are provided in Supplementary Table 2.

### Literature search and study selection strategy

Fourteen bibliographic databases were searched in November 2020: ProQuest ABI/INFORM Global, Campbell Collaboration, Cochrane Library, OvidSP EconLit, OvidSP Embase Classic+Embase, Epistemonikos, Ovid SP Medline, and OvidSP PsycINFO, as well as on Web of Science: Science Citation Index Expanded, Social Sciences Citation Index, Arts & Humanities Citation Index, Conference Proceedings Citation Index–Science, Conference Proceedings Citation Index–Social Science & Humanities, and Emerging Sources Citation Index. The search was structured around 3 concepts using free text and controlled vocabulary: (mandatory OR PPP OR voluntary) AND policy AND diet. Given that some terms refer to several of these concepts at once (e.g., taxes may be classified as both policy and mandatory interventions), 8 different Boolean phrases were conducted and combined at the end (Supplementary Table 3). We did not search specific policy names as this was beyond the scope of our resources. The search strategy in MEDLINE was peer-reviewed by a librarian using the Peer Review of Electronic Search Strategies statement [23] and tested on a sample of 38 potential studies that had been previously identified. Additionally, we screened the publications listed on the NOURISHING database (https://policydatabase.wcrf.org/) and the Global Food Research Program website (https://www.globalfoodresearchprogram.org/). The reference lists of evidence syntheses on cost-effectiveness and of relevant overviews of reviews retrieved in the searches were also checked, as well as those of studies included in 2 other syntheses conducted as part of the overarching project.

Records were uploaded to EPPI-Reviewer Web (EPPI-Centre, University College London, UK) for the removal of duplicates, screening, and data extraction. Both titles and abstract, and eligible full texts, were screened by ≥2 reviewers independently (SR, LB, MJVS, CK, CL) until a 90% agreement rate was reached, representing 12% (*n* = 3346) of titles and abstracts and 33% (*n* = 637) of full texts. The remaining were checked by single reviewers except for records excluded for not being “a policy,” which were all double-checked because disagreements were more common. Disagreements were discussed with a third reviewer in the same review team.

### Data extraction

General policy characteristics extracted included countries and World Bank regions as of 2020 [24], names of national and international policies, policy level (international, national, state), regulatory approach (mandatory, voluntary by the public/nonprofit sectors, by the private sector, PPP, or “mixed” when a policy involved both a mandatory and voluntary component), food policy types by adapting the “NOURIS” part (which focuses on the food environment) of the NOURISHING framework [25]: N-Labeling, O-specific settings including schools, childcare, healthcare and leisure, U-Economic tools, R-Advertising & marketing control, I-Product reformulation by manufacturers, S-Retail and food services environment (excluding those under ‘O’ and ‘I’). Subcategories for each policy type were created iteratively. Initially, PPPs were coded as a separate regulatory approach, but because definitions of voluntary policies and PPPs were not consistent across included records, we treated PPPs as a subcategory of voluntary policies.

Evaluation characteristics consisted of the publication date, general study aim and study design. Six categories of study aims were documented: studies assessing *1*) policy effectiveness, *2*) policy cost-effectiveness, *3*) factors influencing the policy implementation, *4*) factors influencing the policy development, *5*) policy portrayal in the media, and *6*) responses to public consultations. “Effectiveness” included studies assessing the impact of the policy on human health and behavior (e.g., diseases, dietary intake, purchases, use of labels), and effects on the food environment (e.g., characteristics and marketing of food items, including food composition, labels, price, availability, and advertising), as well as the characteristics of the policy itself, for instance, to determine if the latter are aligned with guidelines. As studies did not consistently define effectiveness, policy adherence, compliance, or implementation, when the outcomes mentioned above were assessed, we classified all such study aims as policy “effectiveness” and also noted the type of “participants” evaluated (see below). Regarding study design, additional information was extracted for quantitative studies: *1*) the classification of natural experiments by Leatherdale [26], and *2*) the presence of a relevant comparison group. According to Leatherdale, the most robust natural experiments include time series, follow-up studies, and studies that include a pre-post design and a control (or comparison) group [26]. The comparison groups we considered in the quantitative studies included comparisons of ≥2 policies, a policy compared with none, and participants or products targeted by a policy compared with others not targeted.

Lastly, for studies assessing policy effectiveness, we documented the types of participants employed (i.e., humans, environment characteristics such as food items, documents), the types of outcomes evaluated, and the health equity characteristics considered in the analyses of policy outcomes. A study was considered as exploring an equity characteristic if it compared a policy outcome between different groups by the said characteristic (i.e., not just to describe the sample). The health equity characteristics examined consisted of the sociodemographic characteristics from the PROGRESS-Plus framework. PROGRESS-Plus stands for Place, Race, Occupation, Gender, Religion & culture, Education, SES at the individual level, and Social capital [27]. Age and disability were considered for the “Plus.” In “Place,” in addition to place of residence, we included location of shops. In “Age,” we also considered comparisons between media, menus, and products targeting children/babies compared with adults and between households with and without children. In “Education,” we also considered comparison of school characteristics, e.g., middle compared with high schools. Deprivation indices that encompass a range of PROGRESS-Plus characteristics were coded as SES. We calculated the frequency of each characteristic being evaluated, including throughout the years, and the proportion of the papers that considered ≥1 and ≥2 of them overall.

Except for the publication date and study designs, all categories were nonmutually exclusive, i.e., a publication could have >1. Data were extracted by one reviewer and checked by another (LB, SR, CL), except for the quantitative study designs, which were extracted by one reviewer and 10% were checked by another. A third reviewer was involved to resolve disagreements. No risk of bias or quality appraisal was conducted, as this is not a characteristic of evidence maps given the large number of studies included [19,20].

### Data synthesis

Data were synthesized narratively by the data categories and frameworks mentioned above using descriptive statistics and visuals, including graphs, geographical maps, and tabulations produced in Excel and Word, and an interactive evidence gap map designed with EPPI-Mapper (EPPI-Centre). Drawing from the findings about the policy and evaluation characteristics assessed, we then identified key issues for policy and research, which are highlighted in the discussion.

## Results

**FIGURE 1:**
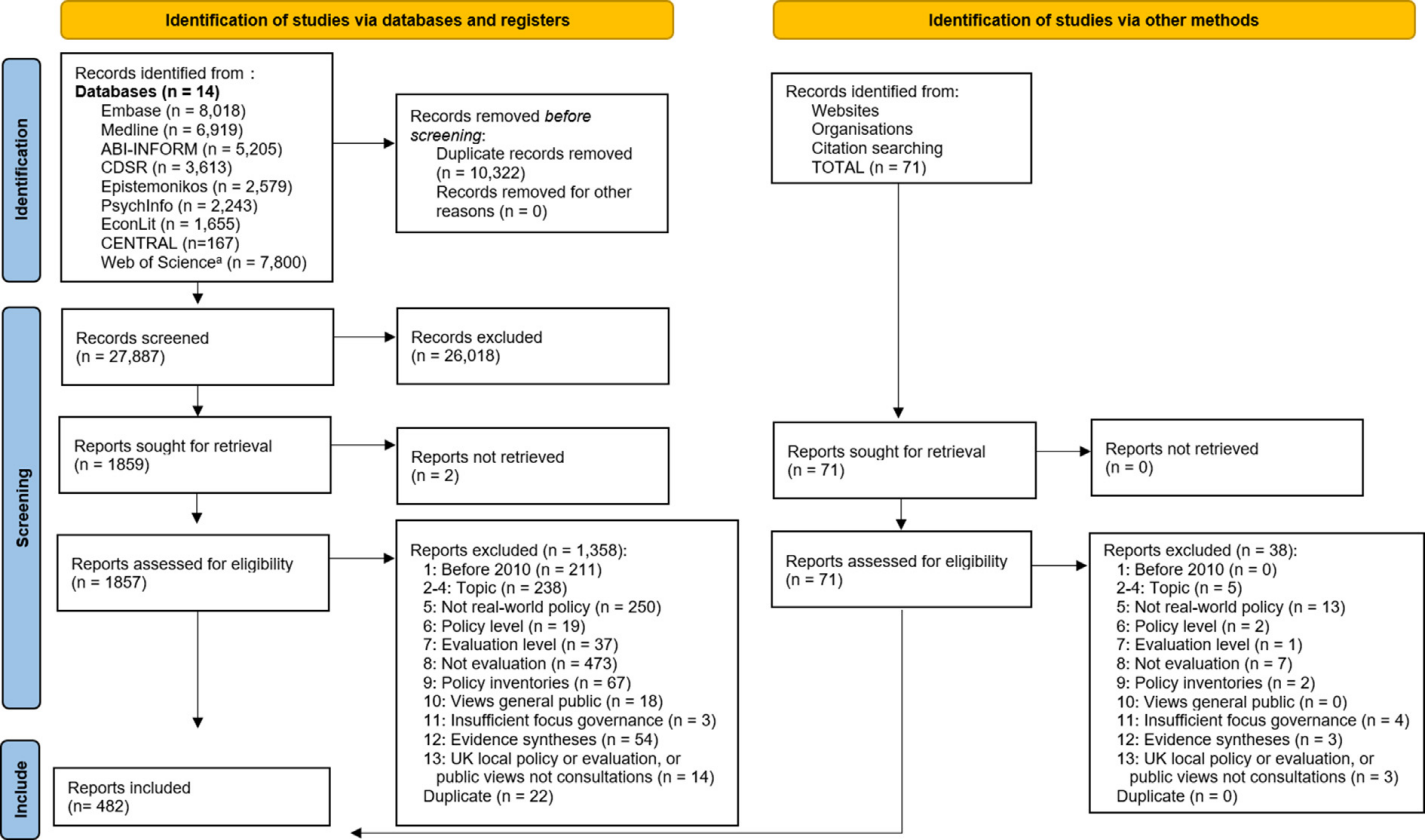
Study selection process for the evidence map (primary studies only) and the overarching project (primary studies and evidence syntheses). ^a^Web of Science included Science Citation Index Expanded, Social Sciences Citation Index, Arts & Humanities Citation Index, Conference Proceedings Citation Index–Science, Conference Proceedings Citation Index–Social Science & Humanities, and Emerging Sources Citation Index. Adapted from the PRISMA template by Page et al. [28].

After removing duplicates, 27,887 records remained and their title and abstract were screened against the eligibility criteria. Of these, 1859 met the criteria and had their full text screened. Another 71 additional full texts were identified on the websites and in reference lists. Overall, 482 publications reporting on primary research evaluations met the eligibility criteria for the evidence map. The selection process is detailed in Figure 1 [28], and the characteristics of the 482 included publications are described in Supplementary Table 4. In accordance with Cochrane guidance [29], the full texts excluded for the least apparent reasons (*n* = 174) are listed in Supplementary Table 5. Given the size of this evidence synthesis, we did not attempt to link publications reporting on the same studies. Thus, the numbers reported refer to publications, not studies.

### Region and country origin of publications

**FIGURE 2:**
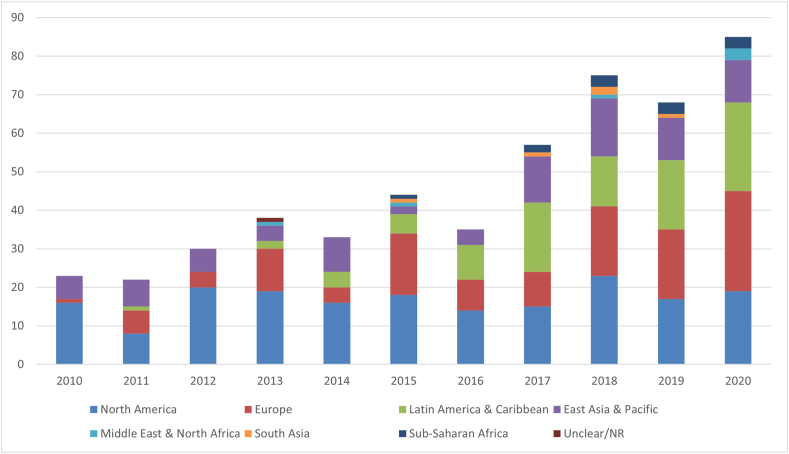
Number of publications by world region and year. Since a publication could cover >1 world region (nonmutually exclusive category), this graph does not reflect the total number of publications per year. NR, not reported.

Figure 2 shows that the number of evaluations published each year on eligible real-world policies has nearly quadrupled over 11 years, ranging from 23 in 2010 to 85 in 2020, but with apparent geographic inequities. Although 70 countries are documented overall, North American countries (i.e., the United States and Canada, representing 38% of publications overall) dominated except in 2017 and 2020. The increase in publications was mainly driven by the World Bank regions of Europe and Central Asia and of Latin American and the Caribbean (25% and 19% of total publications, respectively), although no Central Asian country was documented, and only 3 publications assessed the Caribbean. East Asia and the Pacific were covered by 18% of total publications. Regarding countries, 30% percent (*n* = 146) of publications included the United States, 11% (*n* = 54) the United Kingdom, 10% (*n* = 50) Australia, 9% (*n* = 41) Canada, 8% (*n* = 40) Mexico, 5% (*n* = 25) Brazil, 4% (*n* = 19) Chile, 3% (*n* = 14) France and Spain each, 3% (*n* = 13) Denmark, 2% (*n* = 12) New Zealand, and 2% (*n* = 11) South Africa. The number of publications documenting each country can be found in Supplementary Table 6. Eighty-one percent (*n* = 389) of publications considered these 12 countries alone (without any other country), hereafter referred to as the 12 “dominant” countries. By contrast, 32 countries were included in only 1 or 2 publications each. Although some of the latter were high-income countries (HICs), disparities were startling, with 12 publications found about Sub-Saharan Africa (all but 1 about South Africa), 6 about the Middle East and North Africa (4 of which about Saudi Arabia), and 5 about South Asia (all about India). As some of these evaluations included multiple countries, the level of detail was particularly limited. One publication included unclear locations because it assessed companies’ stock markets [30]. Nearly all publications were in English (*n* = 416); 9 were in Spanish, 2 in Portuguese, and 1 in French.

### Types of policies evaluated

The policies evaluated consisted of 236 national policies (assessed in 73% of publications), 26 “groups” of state policies (e.g., school food policies from different states of the same country were identified under the same label; 26% of publications, all in the 12 dominant countries), 9 international policies (6% of publications), and 1 with both a national and a state component. The names of all policies assessed in the publications (or policy groups for similar policies in different states of a same country) are listed in Supplementary Table 7 by country, along with their policy level, regulatory approach, and number of publications, primarily using information reported by the study authors. When the specific policy name was not reported, we indicated instead the policy type (e.g., back-of-pack labeling).

The 5 most assessed policies consisted of US state school food standards (*n* = 37), the Children’s Food and Beverage Advertising Initiative (a US national voluntary self-regulation advertising industry code, *n* = 21), the national Mexican tax on SSBs (*n* = 19), the national UK Soft Drink Industry Levy (*n* = 14), and various US state SSB taxes (*n* = 14). The latter 3 taxes together represented 54% of publications about economic interventions. The 3 most frequently evaluated international policies were the Australasian Health Star Rating (Australia and New Zealand, *n* = 11), the European Union Pledge (*n* = 5), and the Code of Marketing Breastmilk Substitutes by WHO (*n* = 5).

Using the six “NOURIS” categories, the most assessed policy types were those specific to school, childcare, healthcare and leisure settings (O, *n* = 122, 76% of which were about schools), followed by labeling (N, *n* = 105, 39% about front-of-pack labels), advertising and marketing control (R, *n* = 103, 54% about television advertising alone), and economic interventions (U, *n* = 94, 86% about SSB taxes). Evaluations of product reformulation by manufacturers (I, *n* = 66, 61% about salt) and of the retail and catering sectors (S, *n* = 12, 83% aimed to increase the availability of healthy options) were much less common. Eleven publications assessed policies covering a wide range of categories.

### Regulatory arrangements evaluated

**FIGURE 3:**
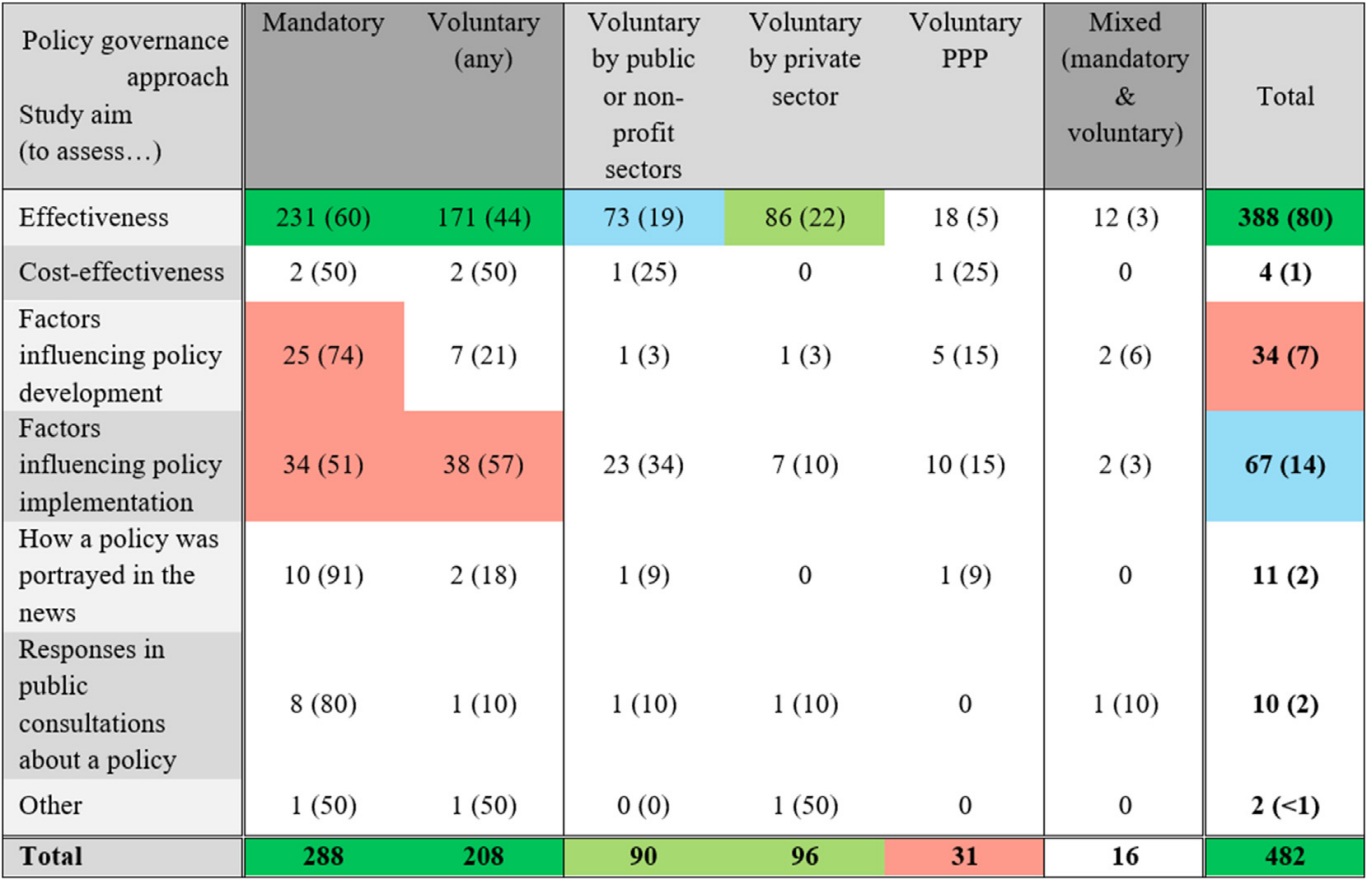
Number (%) of publications by study aim category and regulatory approach. A publication could include >1 study aim and >1 regulatory approach (nonmutually exclusive categories). Legend: Dark green: ≥100 publications; Pale green: 75–99; Blue: 50–74; Red: 25–49; White: <25. PPP, public-private partnership.

Sixty percent (*n* = 288) of publications reported on ≥1 mandatory initiative, 43% (*n* = 208) on ≥1 voluntary arrangement, and 16 assessed mixed approaches (e.g., the combined use of mandatory labeling and voluntary limits for trans-fats in Canada) (Figure 3). Voluntary actions were mainly led by the private (*n* = 96) and public/nonprofit sectors (*n* = 90). Only 31 evaluations investigated PPPs, two-thirds of which were either about the Responsibility Deal in England, UK (*n* = 12) or the Australian Food and Health Dialogue and its continuity, the Healthy Food Partnership (n=10). Mandatory policies were the regulatory arrangement the most commonly assessed in all World Bank regions, ranging from 87% of publications in Latin America and the Caribbean to 55% in Europe, with the exception of voluntary approaches, which represented 57% of publications in East Asia and the Pacific.

The interactive evidence gap map in Supplementary Figure 1 (see HTML file) combines information on policy types, regulatory approaches and world regions. It shows that evaluations about N-labeling were mainly about mandatory approaches in North America (especially menu labeling) and Latin America (especially front-of-pack labeling), followed by the voluntary front-of-pack Health Star Rating in Australia and New Zealand. Most publications on O-specific settings evaluated mandatory initiatives in schools in North America and Europe. Evaluations of U-economic interventions were mostly taxes (and thus mandatory) and concentrated in Latin America, Europe, and the United States. Publications on R-advertising and marketing control were mainly in North America, East Asia and the Pacific, and Europe and predominantly evaluated voluntary actions by the private sector, except in Latin America. Evaluations of I-product reformulation were more equally distributed across the regulatory approaches in the same 4 world regions as above, with more PPP evaluations in East Asia and the Pacific. The few evaluations of policies targeting the retail and catering sectors or using a wide range of categories were only in North America, Europe, and East Asia and the Pacific, and the majority involved the private sector. The 18 evaluations covering Africa, the Middle East, and South Asia documented a variety of policy areas which were mainly mandatory, including 5 publications on SSB taxes.

### Study aims, participants, and outcomes evaluated

The vast majority of publications assessed the effectiveness of a policy (*n* = 388, 80%), followed by factors affecting their implementation (*n* = 67, 14%), factors influencing their development (*n* = 34, 7%), how a policy was portrayed in the news (*n* = 11, 2%), responses to public consultations (*n* = 10, 2%), and cost-effectiveness (*n* = 4, 1%). One investigated whether the New Zealand Advertising Standards Authority self-regulation code protects child rights. Only 25 of the 119 evaluations assessing aspects other than effectiveness covered nondominant countries (although, proportional to the number of publications by country, these evaluations tended to focus more often on nondominant countries). Figure 3 shows the number of publications by study aim and regulatory approach. The majority of evaluations of effectiveness (60%), policy development (74%), news analyses (91%), and public consultations (80%) assessed ≥1 mandatory policy. A slightly greater proportion of evaluations of policy implementation focused on ≥1 voluntary policy (57%), and the 4 cost-effectiveness studies assessed 2 mandatory and 2 voluntary interventions. PPPs were the approach the most holistically assessed with 32% of publications assessing factors influencing implementation and 16% assessing policy development, whereas only 11% of voluntary policies led by private actors were evaluated on aspects other than effectiveness.

The types of participants and outcomes were documented in the effectiveness studies: 166 (43%) relied on data collected via humans only, 181 (47%) did not involve humans at all (i.e., they collected data directly on the food environment, in the news or in documents), and 41 (11%) involved both. Nine percent (*n* = 35) investigated health-related outcomes (e.g., mortality, diseases, disability-adjusted life years, nutritional status, and anthropometrics) and were nearly all conducted in the United States (*n* = 22) followed to a smaller extent by Denmark (*n* = 4) and Portugal (*n* = 3). Other types of outcomes included food environment features (*n* = 255, 66%), human behaviors (e.g., dietary intake, sales, purchases, advertising viewing, use of labels; *n* = 137, 35%), policy characteristics or implementation status (*n* = 22, 6%), and others (*n* = 9, 2%). All policy categories mainly assessed food environment features except for economic interventions (U), which mostly examined human behaviors.

**FIGURE 4:**
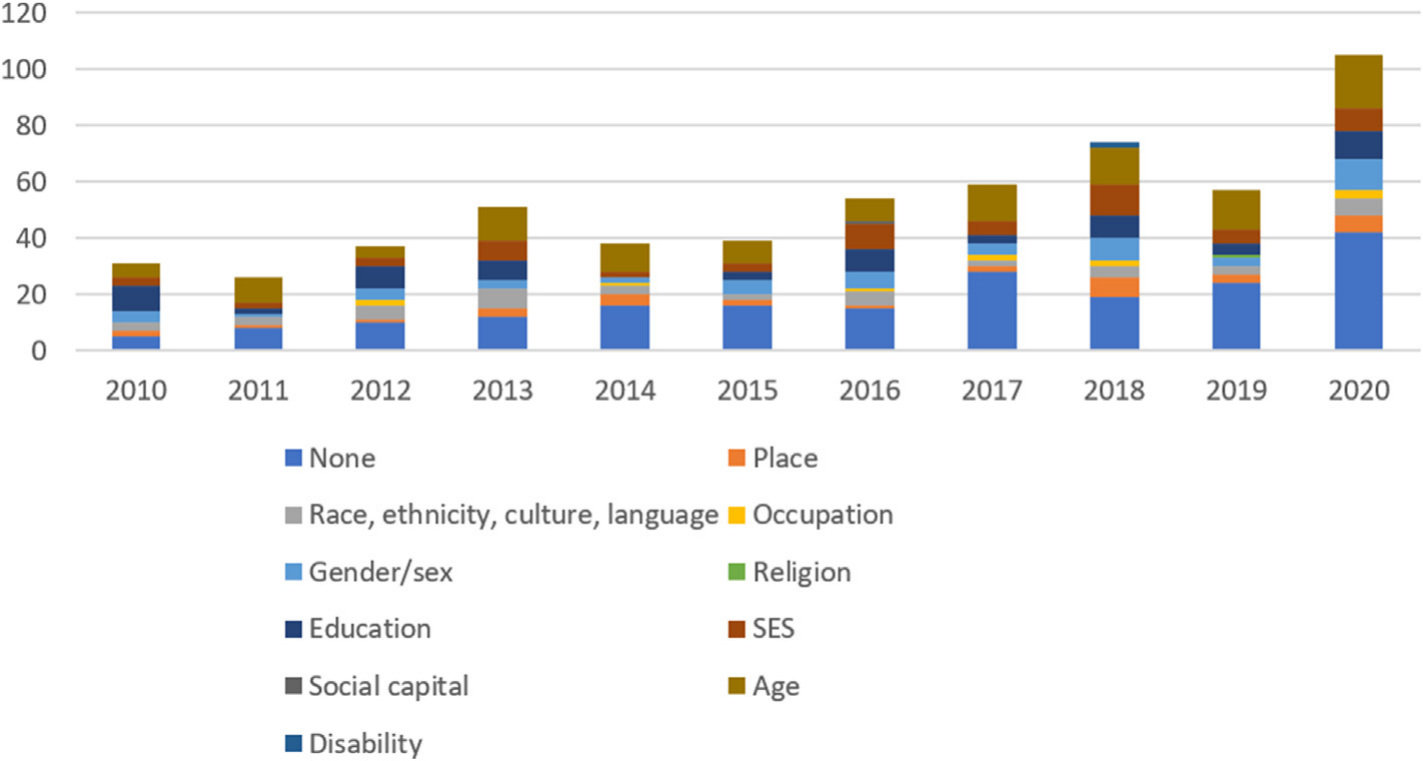
Number of publications by publication year comparing policy outcomes between groups by PROGRESS-Plus characteristics. A publication could include >1 health equity characteristic (nonmutually exclusive category). SES, socioeconomic status.

Among the publications assessing effectiveness (*n* = 388), we also documented those that compared a policy outcome between ≥2 population groups by 10 PROGRESS-Plus equity (or sociodemographic) characteristics. Fifty percent did not consider any equity characteristic, 50% assessed ≥1, and 21% measured ≥2. Age was the most assessed (29%), followed by education (16%, although mainly relating to school characteristics, not the individual level), SES at the individual level (15%, including composite scores), gender/sex (13%), race and culture (11%), and place (8%). Only 11 publications considered occupation, and 1 or 2 examined religion, social capital, and disability each. Equity was most frequently considered in studies from the United States (*n* = 75), Australia (*n* = 20), Canada and Mexico (*n* = 18 each), the United Kingdom (*n* = 17), and Chile and France (*n* = 8 each). Figure 4 illustrates the distribution of effectiveness studies by publication year and equity characteristic. Although the absolute number of publications reporting on ≥1 equity characteristic increased in the most recent years, their proportion reduced from 72% (13 of 18) in 2010 to 40% (28 of 70) in 2020.

### Study designs employed

Table 1 shows the number of publications by study design for the whole map as well as for 3 groups of countries: the 12 dominant countries alone, the United States alone, and the nondominant countries. Most publications employed a quantitative design (*n* = 375, 78%), with one-third of all publications reporting a post cross-sectional study (i.e., one data collection) and one-quarter using a repeat cross-sectional design. Follow-up studies and time series (*n* = 81, 17%), qualitative methods (*n* = 63, 13%), mixed methods (*n* = 27, 6%), policy document analyses alone (*n* = 17, 4%), and modeling or scenarios (*n* = 9, 2%) were less common. However, we only applied the label “follow-up studies” to human participants because nonhuman “participants” such as products and advertisements were generally not the same throughout the evaluation period. Three publications analyzed the implementation phase alone (i.e., pre-pre, between the policy adoption and implementation dates). Compared to studies that only used human-related data, a greater proportion of studies on the environment or documents alone employed both single (33% compared with 46%) and repeat (27% compared with 38%) cross-sectional designs, and fewer follow-up studies/time series (34% compared with 6%) and pre-post designs (52% compared with 32%).

**TABLE 1:** Number of publications by study design in the whole map and for 3 groups of countries.

*N* publications covering…		All countries		The 12 dominantcountries alone^1^		United States alone		Nondominantcountries	
Study designs		*N*	(%)	*N*	(%)	*N*	(%)	*N*	(%)
**Quantitative (all)**		**375**	**(78)**	**309**	**(79)**	**121**	**(86)**	**66**	**(71)**
Follow-up studies and time series	**(all)**	**81**	**(17)**	**68**	**(17)**	**22**	**(16)**	**13**	**(14)**
	Pre-post	69	(14)	58	(15)	13	(9)	11	(12)
	Post-post	12	(2)	10	(3)	9	(6)	2	(2)
Repeat cross-sectional	**(all)**	**121**	**(25)**	**101**	**(26)**	**41**	**(29)**	**20**	**(22)**
	Pre-pre	3	(>1)	3	(>1)	1	(1)	0	(0)
	Pre-post	89	(18)	74	(19)	30	(21)	15	(16)
	Post-post	29	(6)	24	(6)	10	(7)	5	(5)
Cross-sectional, post		164	(34)	133	(34)	58	(41)	31	(33)
Modeling & scenarios		9	(2)	7	(2)	0	(0)	2	(2)
**Qualitative**		63	(13)	44	(11)	13	(9)	19	(20)
**Mixed methods**		27	(6)	21	(5)	4	(3)	6	(6)
**Policy document analysis**		17	(4)	15	(4)	3	(2)	2	(2)
Total		482	(100)	389	(100)	141	(100)	93	(100)

Note: a publication could only include 1 study design (mutually exclusive category).

1 Australia, Brazil, Canada, Chile, Denmark, France, Mexico, New Zealand, South Africa, Spain, United Kingdom, and United States.

Table 1 also highlights that a similar proportion of quantitative designs was employed across the 3 country groupings, with 43%–48% of quantitative studies being single cross-sectional, 30%–34% being repeat cross-sectional, 18%–22% being follow-up studies or time series analyses, and 0%–3% being modeling and scenarios using real-world data. However, compared to nondominant countries, a greater proportion of quantitative studies on the 12 dominant countries alone employed pre-post designs (28% compared with 34%) and relevant comparison groups (i.e., comparisons of different policies, policy compared with none, and participants/products within a policy compared with not; 60% for the 12 dominant countries, 66% for the United States alone, and 44% for nondominant countries). By contrast, qualitative methods were employed proportionally nearly twice as often to evaluate nondominant countries than dominant countries or the United States alone. The majority of publications about Africa, the Middle East, and South Asia were either single cross-sectional or qualitative studies.

By regulatory arrangement, the majority of quantitative studies (61%), follow-up studies and time series analyses (80%), modeling studies (7 of 9), pre-post designs (64%), and qualitative studies (68%) analyzed ≥1 mandatory policy, whereas a greater proportion of mixed methods studies (56%) and policy documents analyses (65%) assessed ≥1 voluntary policy (Table 2). Mandatory policies (32%) and voluntary policies by both public/nonprofit (43%) and private (45%) actors were predominantly evaluated by single cross-sectional studies. PPPs were mostly examined using either repeat cross-sectional or qualitative analyses (29% each). Mixed regulatory policies were mostly assessed with repeat cross-sectional studies (38%). A greater proportion of quantitative publications evaluating voluntary initiatives by the private sector employed relevant comparison groups (78%) compared to the other policy approaches (ranging from 36% for mixed regulatory approaches to 56% for PPPs). By policy type, most follow-up studies/time series analyses and modeling/scenarios evaluated economic interventions. As these were mainly taxes, these study designs also primarily focused on mandatory interventions.

**TABLE 2:** Number of publications by study design and regulatory approach.

Regulatory approach		Mandatory		Voluntary (any)		Voluntary by public or nonprofit sectors		Voluntary by private sector		Voluntary PPP		Mixed (mandatory and voluntary)		Total	
Study design		*N*	(%)	*N*	(%)	*N*	(%)	*N*	(%)	*N*	(%)	*N*	(%)	*N*	(%)
**Quantitative (all)**		**227**	**(61)**	**162**	**(43)**	**74**	**(20)**	**79**	**(21)**	**15**	**(4)**	**11**	**(3)**	**375**	**(78)**
Follow-up studies and time series	**(all)**	**65**	**(80)**	**18**	**(22)**	**6**	**(7)**	**9**	**(11)**	**3**	**(4)**	**2**	**(2)**	**81**	**(17)**
	Pre-post	54	(78)	16	(23)	5	(7)	8	(12)	3	(4)	2	(3)	69	(14)
	Post-post	11	(92)	2	(17)	1	(8)	1	(8)	0	(0)	0	(0)	12	(2)
Repeat cross-sectional	**(all)**	**64**	**(53)**	**58**	**(48)**	**28**	**(23)**	**26**	**(21)**	**9**	**(7)**	**6**	**(5)**	**121**	**(25)**
	Pre-pre	2	(67)	1	(33)	0	(0)	0	(0)	1	(33)	0	(0)	3	(1)
	Pre-post	47	(53)	42	(47)	22	(25)	19	(21)	6	(7)	4	(4)	89	(18)
	Post-post	15	(52)	15	(52)	6	(21)	7	(24)	2	(7)	2	(7)	29	(6)
Cross-sectional, post		91	(55)	84	(51)	39	(24)	43	(26)	3	(2)	3	(2)	164	(34)
Modeling and scenarios		7	(78)	2	(22)	1	(11)	1	(11)	0	(0)	0	(0)	9	(2)
**Qualitative**		43	(68)	20	(32)	7	(11)	6	(10)	9	(14)	4	(6)	63	(13)
**Mixed methods**		12	(44)	15	(56)	8	(30)	6	(22)	1	(4)	1	(4)	27	(6)
**Policy document analysis**		6	(35)	11	(65)	1	(6)	5	(29)	6	(35)	0	(0)	17	(4)

Note: a publication could evaluate >1 regulatory approach (nonmutually exclusive category).

Abbreviation: PPP, public-private partnership.

## Discussion

This evidence map documented the policy and evaluation characteristics of a large body of evidence on a wide range of food environment policies, evaluated over a decade. It included 482 publications assessing their effectiveness, cost-effectiveness, development, and implementation worldwide. Below, we summarize the key results and discuss their implications for policy and research. They are grouped around 3 main issues that emerged from the policy and evaluation characteristics evaluated: *1*) inequities in the origin, design, and scope (aims) of the evidence; *2*) limited availability of evidence on PPPs; and *3*) lack of evaluations of health equity.

### Inequities in the origin, design, and scope of the evidence

Our findings suggest that policy evaluations are published, and likely to be conducted, inequitably across the world in terms of both quantity and quality. Though the number of publications has substantially increased between 2010 and 2020 and covers 70 countries, 81% focused on only 12 countries, and 30% included the United States. Several countries were only documented in multicountry analyses, which provides little detail about them. This geographic imbalance is particularly inequitable because the burden of diet-related diseases is higher in countries and world regions that are absent or underrepresented in the evidence map. For instance, in 2019, the age-standardized mortality rates from noncommunicable diseases attributable to dietary factors were highest in Eastern Europe, Oceania, and Central Asia [31]. Uzbekistan, Solomon Islands, Tajikistan, and Mongolia had among the highest age-standardized rates of diet-related cardiovascular disease mortality [32]. Although arguably not all countries have implemented food environment policies, several have and were not (or barely) captured in the evidence map. For instance, in Eastern Europe, Croatia, Slovenia, Czech Republic, and Poland have government-endorsed voluntary interpretive front-of-pack logos [33]. In Oceania, several islands including Fiji, Kiribati, Marshall Islands, and Nauru have implemented an SSB tax [9]. In Central Asia, Uzbekistan has a national mandatory school meals policy, and Mongolia has adopted a national school policy mandating restrictions on both competitive foods and marketing on foods and drinks [34]. These are but a few examples of policies from which the global food and nutrition research community could learn, especially that their experience is likely to be different from that of the 12 dominant countries. Instead, what our evidence map suggests is that there is lesser evidence available on real-world food environment policies in countries where the needs are particularly high. This reminds us of the “reverse evidence law,” which proposes that although the most upstream health promotion interventions are the most promising, they have the least robust evidence base, both in terms of quantity and quality [35].

Inequities were also detected in the design of quantitative studies, with a greater proportion of publications on the 12 dominant countries employing pre-post designs and relevant comparison groups than publications on nondominant countries. Additionally, there is a need to conduct more longitudinal, time series, and pre-post cross-sectional studies worldwide, as well as more qualitative, mixed methods, and policy document analyses. Most studies on other aspects than effectiveness focused on the 12 dominant countries. The near absence of cost-effectiveness analyses is of particular concern given their key role in policy decisions. Policies need not only to be effective at making the desired change but also to use public resources optimally. Best buy interventions evaluated in one setting may not be cost effective in others, and low- and middle-income countries (LMICs) face greater pressure to identify and prioritize more cost effective and equitable interventions to reduce noncommunicable diseases [13]. The presence of only 35 studies of health-related outcomes in our evidence map suggests that the production of cost-effectiveness studies might be more limited by information on real-world effectiveness rather than on costs. Several cost-effectiveness studies were excluded because they used data from hypothetical policies, from other policies than the one evaluated, or from the policy preadoption or -implementation stages. Lastly, conducting such studies requires specific expertise, and governmental evaluations may not be made publicly available.

Overall, the concentration of evaluations in number, scope, and quality on a few countries suggest that the capacity and funding to assess real-world food environment policies lies in the hands of a few expert teams in a few high- and middle-income countries worldwide. This is different from the classic HIC-LMIC divide in global health. Where countries have implemented food environment policies, evaluations are essential to ensure appropriate measures for the local geographical context, so it is valuable to know that evaluations are absent or not published. Solutions to support both a more equitable geographical representation of the evidence and a better use of resources are likely to include increasing local research and publication capacity, including the conduction of cost-effectiveness studies within policy evaluations more consistently. A systematic meta-narrative review of health research capacity in LMICs highlighted the problem of research agendas being controlled by international funders and HIC researchers [36]. The authors describe a strong focus of research on a handful of diseases rather than on policy and practice as a result and the need to develop research capacity from a systems approach beyond the individual level. This shows the importance to investigate barriers further than a simple lack of resources and capacity or competing priorities.

Although it is possible that we have missed eligible publications (searches were conducted in English, in databases of studies predominantly published in English, with few grey literature), we believe that the systematic map reflects the evidence that is the most easily accessible worldwide and thus the most likely to be used by both researchers and policymakers globally. Indeed, although accessing studies in databases in English can involve substantial paywalls, requires speaking English, and does not reflect local evidence, we think that these sources are still easier to find and to disseminate than grey literature reports and studies in other languages. Searching the latter 2 requires extensive resources, knowing multiple languages, and is unlikely to fill the gaps mentioned above.

### Limited availability of evidence on PPPs

Our evidence map shows that PPPs have been very little evaluated at the international, national, and state levels compared to other regulatory approaches. Indeed, only 32 (6.6%) publications are about PPPs, of which two-thirds are about the same 2 partnerships in England and Australia. PPPs have not only been encouraged by leading international organizations as an effective way to promote health; they are presented as good practice and the gold standard in core global health commitments, from the Declaration of Alma Ata in 1978 [37] to the UN Sustainable Development Goals in 2015 [38]. Yet, in a systematic review that was part of our larger project, we reviewed the studies that assessed the effectiveness of PPPs for making food environments healthier (*n* = 17) [21]. The results showed a lack of effectiveness in addition to the PPPs having limited scopes, participation, and monitoring. These equivocal results and the limited evidence on PPPs in this evidence map question both the ability of PPPs to promote healthy food environments and how they have gained such traction at the global level as a means to promote health [21]. One of the arguments that the food industry uses is that it needs to be “part of the solution.” Yet, a growing body of evidence on the commercial determinants of health stresses the presence of competing interests between the public sector and the food industry and thus between health promotion and corporate profits [39,40]. Some studies suggest that by using extensive lobbying and presenting themselves as partners to public authorities, corporate actors can have a direct influence on governmental processes and reduce the risk of regulation [[41], [42], [43]].

### Lack of evaluations of health equity

In 2008, the WHO Commission on Social Determinants of Health prioritized the measurement and evaluation of equity as 1 of the 3 key principles of action for tackling health inequalities [44]. However, our evidence map revealed that only half of the publications that assessed policy effectiveness compared outcomes by any equity characteristic and that this proportion has decreased over time. This was despite using a generous interpretation of the PROGRESS-Plus framework that considered the characteristics of products and settings in addition to that of human participants. Equity analyses most frequently commented on age, education (mainly school characteristics), or SES at the individual level. Occupation and education at the individual level, religion and culture, social capital, and disability were barely considered (although we recognize that social capital is more challenging to operationalize and capture). This means that the equity assessments are missing key vulnerable populations. The lack of evaluations of food environment policies focusing on equity has been reported in 2 other overviews of reviews (one of which is part of our larger project) [21,45]. Both called for more primary research in this area. To expand the evidence base and support decision makers in the reduction of health inequities, researchers should include the assessment of differential impacts of food environment policies by sociodemographic characteristics, with an emphasis on those neglected to date. Developing guidance for researchers specific to food environment policy evaluations, with examples for studies of products, settings, and documents in addition to human studies, might be helpful because current generic guidance largely focuses on the latter [27].

### Strengths and limitations

To our knowledge, this is the first evidence synthesis that covers such a wide range of real-world food environment policies. It demonstrates the value of evidence maps beyond identifying trends and gaps in research: they can also be employed to critically analyze evidence from a policy perspective, including the scientific evidence itself, the policy instruments employed, and equity implications. The literature search for this evidence map was extensive, and a very large amount of literature was reviewed and synthesized. This provides a comprehensive overview of the global landscape of published literature in this field. A strength of the analysis is that our policy categories are largely aligned with those of the INFORMAS framework [46] (with the exception of health and nutrition claims and food trade and investments). The INFORMAS framework is a guide developed by a global network, and recognized by WHO, for monitoring and benchmarking public and private sector interventions aiming to improve food environments.

Screening by 2 independent reviewers for only 12% of titles and abstracts and 33% of full texts might be seen as a limitation, but these actually represent 3346 titles and abstracts and 637 full text articles. We aimed for a 90% agreement rate before moving to individual screening, and all records excluded for reasons with a lower rate were double-checked.

Regarding potential biases, it is reasonable to suppose that publication bias will have particularly operated on the grey literature and studies not in English included, as they are more likely to have positive findings than those unretrieved. The possibility of publication and selective outcome bias relating to observational study designs should also be considered (especially regarding equity as an outcome) given that preregistration of such studies and their protocol is not mandatory [47]. The small number of publications on the retail and catering sectors is likely to reflect the current limited capacity to implement such initiatives at the state or national level rather than biases in the search strategy.

## Conclusions

This evidence map reveals striking imbalances in the global evidence on policies promoting healthy food environments. It raises questions on the lack of published evaluations in multiple countries and on the evidence used for promoting PPPs in food environment policies. Lastly, it calls for greater consideration of equity in policy evaluations.

## Author contributions

The authors’ responsibilities were as follows – LB: designed the study, conducted the literature searches, analyzed the data, drafted the manuscript and has primary responsibility for final content; LB, SR, CL, CK, MJVS: conducted the research, including screening references and/or extracting data; HR, ME, MP: provided feedback throughout the project duration; MPK, CB, PL: provided feedback as part of the project’s Study Scientific Committee; and all authors: read and approved the final manuscript.

## Funding

This work was supported by the Public Health Research Programme of the National Institute for Health and Care Research (England), grant number PHR NIHR128607 (obtained by CK (PI), ME, CL, MP, and HR).

## Data availability

Data described in the manuscript will be made available upon request pending application and approval.

## Conflict of interest

All authors declare no conflicts of interest. HR, MP and CK have funding through the SPECTRUM consortium which is funded by the UK Prevention Research Partnership, a consortium of UK funders (UK research and innovation research councils [Medical Research Council, Engineering and Physical Sciences Research Council, Economic and Social Research Council, and Natural Environment Research Council], charities [British Heart Foundation, Cancer Research UK, the Wellcome Trust, and The Health Foundation], and government [Scottish Government Chief Scientist Office, Health and Care Research Wales, NIHR, and Public Health Agency]). The funders were not involved in any part of the development, delivery or publication of this study.
