# Supplementary material for: Inequalities in Research on Food Environment Policies: An Evidence Map of Global Evidence from 2010-2020
Source: Adv Nutr. 2024 Sep 23;15(11):100306. doi: 10.1016/j.advnut.2024.100306 (PMC11555335; doi:10.1016/j.advnut.2024.100306)
Supplement: multimedia component 1 [file mmc1.docx]

Supplementary Material

Contents

[Supplementary Table 1: Preferred Reporting Items for Systematic reviews and Meta-Analyses extension for Scoping Reviews (PRISMA-ScR) Checklist 2](#_Toc165403283)

[Supplementary Table 2: Detailed eligibility criteria 4](#_Toc165403284)

[Supplementary Table 3: Search strategy on OvidSP Medline 7](#_Toc165403285)

[Supplementary Table 4: Characteristics of included studies (n=482) 10](#_Toc165403286)

[Supplementary Table 5: Full-texts excluded for selected reasons (n=174) 53](#_Toc165403287)

[Supplementary Table 6: Number of publications by World Bank region and country (n=482) 58](#_Toc165403288)

[Supplementary Table 7: Name of the policies (and policy groups) included by country, policy level and N publications 60](#_Toc165403289)

# Supplementary Table 1: Preferred Reporting Items for Systematic reviews and Meta-Analyses extension for Scoping Reviews (PRISMA-ScR) Checklist

| **SECTION** | **ITEM** | **PRISMA-ScR CHECKLIST ITEM** | **REPORTED ON PAGE #** |
| --- | --- | --- | --- |
| **TITLE** | | | |
| Title | 1 | Identify the report as a scoping review. | Title page – evidence map |
| **ABSTRACT** | | | |
| Structured summary | 2 | Provide a structured summary that includes (as applicable): background, objectives, eligibility criteria, sources of evidence, charting methods, results, and conclusions that relate to the review questions and objectives. | Abstract |
| **INTRODUCTION** | | | |
| Rationale | 3 | Describe the rationale for the review in the context of what is already known. Explain why the review questions/objectives lend themselves to a scoping review approach. | 1-2 |
| Objectives | 4 | Provide an explicit statement of the questions and objectives being addressed with reference to their key elements (e.g., population or participants, concepts, and context) or other relevant key elements used to conceptualize the review questions and/or objectives. | 3 |
| **METHODS** | | | |
| Protocol and registration | 5 | Indicate whether a review protocol exists; state if and where it can be accessed (e.g., a Web address); and if available, provide registration information, including the registration number. | 3 |
| Eligibility criteria | 6 | Specify characteristics of the sources of evidence used as eligibility criteria (e.g., years considered, language, and publication status), and provide a rationale. | 3-4 + Suppl Table 2 |
| Information sources* | 7 | Describe all information sources in the search (e.g., databases with dates of coverage and contact with authors to identify additional sources), as well as the date the most recent search was executed. | 4-5 |
| Search | 8 | Present the full electronic search strategy for at least 1 database, including any limits used, such that it could be repeated. | Suppl Table 3 |
| Selection of sources of evidence† | 9 | State the process for selecting sources of evidence (i.e., screening and eligibility) included in the scoping review. | 5 |
| Data charting process‡ | 10 | Describe the methods of charting data from the included sources of evidence (e.g., calibrated forms or forms that have been tested by the team before their use, and whether data charting was done independently or in duplicate) and any processes for obtaining and confirming data from investigators. | 5-7 |
| Data items | 11 | List and define all variables for which data were sought and any assumptions and simplifications made. | 5-7 |
| Critical appraisal of individual sources of evidence§ | 12 | If done, provide a rationale for conducting a critical appraisal of included sources of evidence; describe the methods used and how this information was used in any data synthesis (if appropriate). | N/A |
| Synthesis of results | 13 | Describe the methods of handling and summarizing the data that were charted. | 7 |
| **RESULTS** | | | |
| Selection of sources of evidence | 14 | Give numbers of sources of evidence screened, assessed for eligibility, and included in the review, with reasons for exclusions at each stage, ideally using a flow diagram. | 7-8 + Fig. 1 + Suppl Table 5 |
| Characteristics of sources of evidence | 15 | For each source of evidence, present characteristics for which data were charted and provide the citations. | Suppl Table 4 (no citation) |
| Critical appraisal within sources of evidence | 16 | If done, present data on critical appraisal of included sources of evidence (see item 12). | N/A |
| Results of individual sources of evidence | 17 | For each included source of evidence, present the relevant data that were charted that relate to the review questions and objectives. | Suppl Table 4 |
| Synthesis of results | 18 | Summarize and/or present the charting results as they relate to the review questions and objectives. | 8-15 |
| **DISCUSSION** | | | |
| Summary of evidence | 19 | Summarize the main results (including an overview of concepts, themes, and types of evidence available), link to the review questions and objectives, and consider the relevance to key groups. | 15, 17, 18 |
| Limitations | 20 | Discuss the limitations of the scoping review process. | 19-20 |
| Conclusions | 21 | Provide a general interpretation of the results with respect to the review questions and objectives, as well as potential implications and/or next steps. | 20 |
| **FUNDING** | | | |
| Funding | 22 | Describe sources of funding for the included sources of evidence, as well as sources of funding for the scoping review. Describe the role of the funders of the scoping review. | For the evidence map only: Title page |

* Where *sources of evidence* (see second footnote) are compiled from, such as bibliographic databases, social media platforms, and Web sites.

† A more inclusive/heterogeneous term used to account for the different types of evidence or data sources (e.g., quantitative and/or qualitative research, expert opinion, and policy documents) that may be eligible in a scoping review as opposed to only studies. This is not to be confused with *information sources* (see first footnote).

‡ The frameworks by Arksey and O’Malley (6) and Levac and colleagues (7) and the JBI guidance (4, 5) refer to the process of data extraction in a scoping review as data charting*.*

§ The process of systematically examining research evidence to assess its validity, results, and relevance before using it to inform a decision. This term is used for items 12 and 19 instead of "risk of bias" (which is more applicable to systematic reviews of interventions) to include and acknowledge the various sources of evidence that may be used in a scoping review (e.g., quantitative and/or qualitative research, expert opinion, and policy document).

*From:* Tricco AC, Lillie E, Zarin W, O'Brien KK, Colquhoun H, Levac D, et al. PRISMA Extension for Scoping Reviews (PRISMAScR): Checklist and Explanation. Ann Intern Med. 2018;169:467–473. [doi: 10.7326/M18-0850](http://annals.org/aim/fullarticle/2700389/prisma-extension-scoping-reviews-prisma-scr-checklist-explanation).

# Supplementary Table 2: Detailed eligibility criteria

Note: This coding tool is intended to be used in the order presented, e.g., studies must pass criterion 1 to be assessed for criterion 2.

| **EXCLUSION CODE** | **INCLUDED** | **EXCLUDED** |
| --- | --- | --- |
| **1.1: Before 2010** | Published between 2010 and 2020 | Published before 2010 |
| **1.2: After 2020** | Published from 2021 | Published after 2020 |
| **2-3:**  **- Not diet,**  **- Not food environment,**  **- Excluded food environment topics** | Policies that…  - Apply to “ordinary” food including baby formulas, e.g. as in the Eatwell guide (NHS 2019);  AND  - Aim to promote healthy food environments (i.e., food proximity, affordability, marketing and information – as per FAO’s definition) of public spaces. Information relates to information about products and health, such as on labels and displays; not education activities.   AND  - Aims to address the main dietary risk factors associated with the burden of disease in England | - Not diet-related or not targeting ordinary food, e.g., “natural” products, supplements, alcohol, functional foods, gluten free, GMOs, sweeteners, ingredients not intended to be sold to the general public (e.g., emulsifiers);  - Breastfeeding since it is produced and available differently than for other food and drinks;  - Not on the food environment, e.g., education campaigns and interventions, clinical or therapeutic interventions;  - Health claims, food fortification, and international trade (e.g. exports/imports), finance, regulation of free markets, customs duties despite being on the food environment.  - General taxes (e.g. sales taxes not specific to food, taxes on income), tax evasion, service charge; - Food safety, hygiene, allergies, accuracy of nutrition values on labelling;  - Energy drinks when evaluations are about their interaction with alcohol or caffeine since this is more of a safety issue. **INCLUDE energy drinks when they are considered as a sugary drink**;  - Agriculture and farming as a primary focus rather healthy diet (e.g. involving local farms in schools to support the latter rather than to improve health);  - Food security, undernutrition, or double/triple burden of malnutrition, e.g., feeding/supplementation programmes, IYCF for undernutrition, vouchers, cash-transfer programmes, food and milk banks & other food redistribution programmes, international food aid or assistance, because interventions relevant for England tend to target individuals rather than environments **INCLUDE free school meals that are considered from a school food environment perspective;**  - Food sustainability as a primary focus rather than healthy diet (e.g. research on organic products, climate change or animal welfare not aiming to improve diet directly);  - About research collaboration as a topic;  - Historic research. |
| **4: Not general population** | The policies aim to improve the health of the general public. This includes:  - The general public in a workplace accessible to all, e.g., clients in a restaurant, visitors in a hospital canteen;  - Children in schools and nurseries. | The policy only targets:  -  Staff in a workplace;  -  Patients or people with specific health conditions;  -  Athletes;  - The army. |
| **5: Not real-life policy** | Data were collected:  - At least once when the policy was adopted or implemented; or  - as part of a state or national public consultation.  Experiments, simulations and projections are included when based on ‘real-world’ policy data defined as above | - Experiments in non-real-life environments, e.g., online or in laboratories; - Studies that only use data collected before the policy was adopted or implemented, or testing policy scenarios.  - Research initiatives e.g. a trial testing a new approach that is not part of a state/national/international policy);  - Evidence syntheses and comparative studies that have a great proportion of policies not meeting this criterion and that don’t exclude them from at least part of the analyses, e.g. using subgroup analyses. |
| **6: Local**  **POLICY** **level** | Policies implemented at the:  - International level;  - National level;  - Provincial/state level;  - Anywhere from the UK passes this criterion (but see EX13). | - Policies implemented at a lower level than the state **(except in the UK, which passes this criterion).**  - Evidence syntheses and comparative studies that have a great proportion of policies not meeting this criterion and that don’t exclude them from at least part of the analyses, e.g. using subgroup analyses. |
| **7: Local EVALUATION level** | Evaluations conducted:  - At a state, national or international level  - In the UK (any level passes this criterion, but see EX13);  - Not at a state level but represent a big part of a country, or multiple places across a country or state;  - Audits of food products, shops and TV adverts can be conducted at any level unless the evaluation specifies focusing on local independent stores or TV channels | - Evaluations conducted at a lower level than state, e.g., city, or a few institutions not selected to represent a state or more;  -  Evaluations that cannot represent a country or state, e.g., conducted in 2 schools.  - Evidence syntheses that have a great proportion of evaluations not meeting this criterion and that don’t exclude them from at least part of the analyses, e.g. using subgroup analyses. |
| **8: Not evaluation** | - Primary research evaluations  - Evidence syntheses that have searched at least 2 databases, mention eligibility criteria, AND clearly indicate which studies are included (e.g., in a table, series of references at the start of the results section or within each section without needing to track down each reference to make the whole list). | - Reviews that do not meet the inclusion criteria for evidence syntheses identified to the left;  - Theoretical papers, commentaries, viewpoints, editorials, letters;  - Conference abstracts, dissertations, theses;  - Study protocols, working papers, pre-prints.  - Websites, blogs, podcasts, book reviews, book chapters; |
| **9: Policy inventories** | Studies assessing effectiveness, cost-effectiveness, factors influencing policy development or implementation, responses to public consultations, or media coverage of a policy | Studies solely inventorying (‘mapping’) the presence of policies in countries or regions and/or benchmarking their implementation. |
| **10: Views about the general public** | - Studies assessing the views of the general public as part of a state or national public consultation;  - Studies assessing the views of the public in the UK pass this criterion, but see EX13. | - Studies assessing the views of the general public **(except in the UK in which case they are INCLUDED);**  - Evidence syntheses that have a great proportion of studies not meeting this criterion and that don’t exclude them from at least part of the analyses, e.g. using subgroup analyses. |
| **11 :**  **Insufficient focus on governance (multiple policies)** | - Primary research evaluations assessing multiple policies and that indicate, at least broadly, the governance approach of these or analyse them by governance approach  - Evidence syntheses that analyse policies taking their governance approach into account | - Primary research evaluations assessing multiple policies that have unclear governance  - Evidence syntheses that do not consider governance in their analysis (including when the governance approach of each policy is stated but the analysis does not consider it). |
| **12: Not primary study** | - Primary research evaluations; | Evidence syntheses of primary studies and overview of reviews (also called umbrella reviews) |
| **13**  **- UK local level**  **- UK views of general public** | (This is simply to group studies about the UK together)  - Studies not focusing on local policies or evaluations (as defined in EX 6 and EX-7) in the UK  - Studies not assessing the views of the general public in the UK outside public consultations | - Studies focusing on local policies or evaluations (as defined in EX 6 and EX-7) in the UK  - Studies assessing the views of the general public in the UK outside public consultations  - Evidence syntheses that have a great proportion of studies not meeting this criterion and that don’t exclude them from at least part of the analyses, e.g. using subgroup analyses. |
| **Duplicate** | Documents that are not identical. | Identical documents (only keep one of them). |
| **INCLUDE** | Publications passing ALL the inclusion criteria above | Publications failing at one of the exclusion criteria above or more. |

# Supplementary Table 3: Search strategy on OvidSP Medline

The eight key search lines are highlighted in pale grey.

1 exp Diet/

2 exp Food/

3 beverages/ or exp artificially sweetened beverages/ or exp carbonated beverages/ or exp coffee/ or exp drinking water/ or exp energy drinks/ or exp “fruit and vegetable juices”/ or exp milk/ or exp milk substitutes/ or exp sugar-sweetened beverages/

4 exp Fruit/

5 exp Vegetables/

6 exp Sodium, Dietary/

7 exp Sugars/

8 exp Fats/

9 exp Dietary Fiber/

10 exp Portion Size/ or exp Serving Size/

11 exp Infant Food/ or exp Infant Formula/

12 (Diet or Nutrition or Food or foods or Snack or snacks or Drink or drinks or Beverage* or Soda or sodas or Fruit or fruits or Vegetable* or Salt or Sodium or Sugar* or Fat or fats or fatty acids or TFAs or Fibre or fibres or fiber or fibers or “Portion size*” or “Serving size*” or Menu or menus or Infant formula or infant formulas or baby formula or baby formulas or baby milk or infant milk or artificial milk or breastmilk substitute* or breast milk substitute*).ti,ab.

13 1 or 2 or 3 or 4 or 5 or 6 or 7 or 8 or 9 or 10 or 11 or 12 ***[Food free + MeSH terms]***

14 exp Legislation, Food/ ***[semi-final line 1; for food policies that are clearly regulatory]***

15 (Law or laws or Legislat* or Regulat* or Decree or “Executive order” or Tax or taxes or taxation or taxed or taxing or Levy or levies or levied or “Excise duty” or “fiscal policy” or “fiscal policies” or “fiscal measure” or “fiscal measures”).ti,ab. ***[terms related to policies that are regulatory]***

16 ((Law or laws or Legislat* or Regulat* or Decree or “Executive order” or Tax or taxes or taxation or taxed or taxing or Levy or levies or levied or “Excise duty” or “fiscal policy” or “fiscal policies” or “fiscal measure” or “fiscal measures”) adj5 (Diet or Nutrition or Food or foods or Snack or snacks or Drink or drinks or Beverage* or Soda or sodas or Fruit or fruits or Vegetable* or Salt or Sodium or Sugar* or Fat or fats or fatty acids or TFAs or Fibre or fibres or fiber or fibers or “Portion size*” or “Serving size*” or Menu or menus or Infant formula or infant formulas or baby formula or baby formulas or baby milk or infant milk or artificial milk or breastmilk substitute* or breast milk substitute*)).ti,ab. ***[semi-final line 2; 15 adj5 12, for policies that are regulatory + food free terms]***

17 exp Fiscal Policy/ or exp Taxes/

18 exp Government Regulation/

19 17 or 18 ***[MeSH terms associated with policies that are regulatory or about governance]***

20 19 and 13 ***[semi-final line 3; MeSH policies that are regulatory + food]***

21 (“Public-private partnership*” or “Responsibility Deal”).mp. ***[terms clearly related to PPPs]***

22 exp Public-Private Sector Partnerships/

23 21 or 22 ***[free key words + MeSH clearly about PPP]***

24 23 and 13 ***[semi-final line 4; clearly PPP + food]***

25 (Regulatory or Compulsory or Obligat* or obliged or Voluntary or Option* or Non-compulsory or Non-regulatory or Non-obligatory or Public-Private).mp. ***[terms related to governance]***

26 exp Regulatory Reporting/ or exp Regulatory Programs/

27 exp Voluntary Programs/

28 25 or 26 or 27 ***[free and MeSH terms related to governance]***

29 exp Nutrition Policy/

30 exp Food Labeling/

31 exp Food Assistance/

32 29 or 30 or 31 ***[policies that are clearly about food]***

33 28 and 32 ***[semi-final line 5; governance + food-related policies]***

34 (Government* or Governance or Minist* or Senate or ((National or federal or state or provincial) adj (department or agency or institute))).ti,ab. ***[free words related to the national or state public sector]***

35 government/ or exp federal government/ or exp government agencies/ or exp state government/

36 (Industry or industries or Private or Business* or Public-private or Company or companies or Corporat* or Multinational* or Vendor* or Retail* or Shop or shops or Store or stores or supermarket* or Restaura* or Broadcaster*).ti,ab. ***[free terms related to relevant private sectors]***

37 exp Food-Processing Industry/ or exp Food Industry/

38 exp Restaurants/

39 exp Food Services/

40 34 or 35 or 36 or 37 or 38 or 39 ***[free and MeSH terms about the public and private sectors]***

41 (Policy or policies or Plan or Strategy or strategies or Standard or standards or Scheme* or Program* or Guide or guides or guidance or guidelines or Code or codes or Measure or Measures or Rulebook or Target or targets or Limit or limits or limitation or Reformulat* or Remov* or Restrict* or Prohibit* or Ban or bans or banned or Label* or Population intervention* or population-level intervention* or population-based intervention*).ti,ab. ***[free terms frequently used to name diet-related policies]***

42 exp Policy Making/

43 41 or 42 ***[free and MeSH terms about policy]***

44 13 and 28 and 40 and 43 ***[semi-final line 6; food + governance + public/private actors + policy]***

45 (Agreement* or Alliance* or Coalition* or Collaboration or Cooperation or “Joint deliver*” or Partnership* or Pledge* or Self-regulat*).ti,ab. ***[free terms related to partnership]***

46 ((Agreement* or Alliance* or Coalition* or Collaboration or Cooperation or “Joint deliver*” or Partnership* or Pledge* or Self-regulat*) adj5 (Industry or industries or Private or Business* or Public-private or Company or companies or Corporat* or Multinational* or Vendor* or Retail* or Shop or shops or Store or stores or supermarket* or Restaura* or Broadcaster*)).ti,ab. ***[45 adj5 36, to identify partnerships with private actors free terms]***

47 46 and 13 ***[semi-final line 7; partnerships with private actors + food]***

48 (“policy option” or “policy options”).mp.

49 48 and 13 ***[semi-final line 8; policy options + food]***

50 14 or 16 or 20 or 24 or 33 or 44 or 47 or 49 ***[combination of the 8 strategies]***

51 exp Pharmacology/

52 exp Food Safety/

53 exp Hygiene/

54 exp Food Hypersensitivity/

55 exp Genetics/

56 exp Toxicology/

57 exp cell physiological phenomena/ or exp genetic phenomena/ or exp microbiological phenomena/

58 exp heterocyclic compounds/ or exp polycyclic compounds/ or exp macromolecular substances/ or exp “hormones, hormone substitutes, and hormone antagonists”/ or exp “enzymes and coenzymes”/ or exp “nucleic acids, nucleotides, and nucleosides”/ or exp complex mixtures/ or exp biological factors/ or exp “biomedical and dental materials”/

59 (Cell* or mitochondr* or abell* or mononucl* or nucle* or reductase or abellin* or oxydat* or oxidase or homeostas* or overexpress* or phenotype* or embryo* or abellinge* or PCR or RNA or gene or genes or genetic* or ((calcium or salt or sodium) adj2 ion)).mp.

60 exp animals/ not humans/

61 exp Animal Experimentation/

62 exp Hydrocarbons/

63 exp Forensic Genetics/

64 exp pharmacologic actions/

65 exp plant extracts/ or exp prescription drugs/

66 exp Drug Therapy/

67 exp Biopharmaceutics/

68 51 or 52 or 53 or 54 or 55 or 56 or 57 or 58 or 59 or 60 or 61 or 62 or 63 or 64 or 65 or 66 or 67

69 50 not 68

70 limit 69 to yr=”2000 -Current”

71 exp address/ or exp bibliography/ or exp biography/ or exp collected work/ or exp collection/ or exp comment/ or exp congress/ or exp dataset/ or exp dictionary/ or exp directory/ or exp editorial/ or exp guideline/ or exp lecture/ or exp letter/ or exp news/ or exp newspaper article/ or exp overall/ or exp periodical index/ or exp video-audio media/ or exp webcast/

72 70 not 71

# Supplementary Table 4: Characteristics of included studies (n=482)

| **1st author (date)**  **(internal ID)** | **Publication title** | **World Bank regions (countries)** | **Policy categories** | **Policy levels** | **Governance approaches** | **Study aims** | **Study design** | **Participants & outcomes (effectiveness studies only)** | **Equity characteristics (effectiveness studies only)** |
| --- | --- | --- | --- | --- | --- | --- | --- | --- | --- |
| Abreu (2018) (ID:63778492) | Cardiovascular disease and high blood pressure trend analyses from 2002 to 2016: after the implementation of a salt reduction strategy | Euro (Portugal) | N- Labelling I- Reformulat | National | Mixed | Effectiveness | Quantitative (pre-post FU/TS) | Humans (health) | Gender/sex Age |
| Abreu (2020) (ID:52966944) | Impact of public health initiatives on acute coronary syndrome fatality rates in Portugal | Euro (Portugal) | N- Labelling I- Reformulat | National | Mixed | Effectiveness | Quantitative (pre-post FU/TS) | Humans (health) | Gender/sex Age |
| Aburto (2017) (ID:52968657) | Taxing Snacks: Impact in Price and Consumption in Mexico | L Am & Car (Mexico) | U- Economic | National | Regulatory | Effectiveness | Quantitative (pre-post FU/TS) | Humans (behaviour) Non-hum (food envt) | (none) |
| Acton (2018) (ID:52947916) | School Food Policies and Student Eating Behaviors in Canada: Examination of the 2015 Cancer Risk Assessment in Youth Survey | N Am (Canada) | O- Settings | State | Volunt (public/non-profit) Regulatory | Effectiveness | Quantitative (post CS) | Humans (behaviour) | Education |
| Alarcon-Calderon (2020) (ID:52946462) | Lack of nutrient declarations and low nutritional quality of pre-packaged foods sold in Guatemalan supermarkets | L Am & Car (Guatemala) | N- Labelling | National | Volunt (public/non-profit) | Effectiveness | Quantitative (post CS) | Non-hum (food envt) | (none) |
| Alkhaldy (2020) (ID:52946693) | Response of the public and restaurant owners to the mandatory menu energy-labelling implementation in restaurants in Saudi Arabia | M East & N Afr (Saudi Arabia) | N- Labelling | National | Regulatory | Implementation Effectiveness | Quantitative (post CS) | Humans (food envt, behaviour) | (none) |
| Allais (2015) (ID:52935453) | Mandatory labels, taxes and market forces: An empirical evaluation of fat policies | Euro (France) | N- Labelling | National | Mixed | Effectiveness | Quantitative (post CS) | Humans (behaviour) | Gender/sex SES Age |
| Allemandi (2019) (ID:52947165) | Monitoring sodium content in processed foods in Argentina 2017-2018: Compliance with national legislation and regional targets | L Am & Car (Argentina) | I- Reformulat | National | Regulatory | Effectiveness | Quantitative (post CS) | Non-hum (food envt) | (none) |
| Alsukait (2020) (ID:52944381) | Evaluating Saudi Arabia's 50% Carbonated Drink Excise Tax: Changes in Prices and Volume Sales | M East & N Afr (Saudi Arabia) | U- Economic | National | Regulatory | Effectiveness | Quantitative (pre-post FU/TS) | Humans (behaviour)  Non-hum (food envt) | (none) |
| Alvarado (2017) (ID:52948096) | Trends in beverage prices following the introduction of a tax on sugar-sweetened beverages in Barbados | L Am & Car (Barbados) | U- Economic | National | Regulatory | Effectiveness | Quantitative (pre-post FU/TS) | Humans (food envt) | (none) |
| Alvarado (2019) (ID:52958479) | Assessing the impact of the Barbados sugar-sweetened beverage tax on beverage sales: an observational study. | L Am & Car (Barbados) | U- Economic | National | Regulatory | Effectiveness | Quantitative (pre-post FU/TS) | Humans (behaviour) | (none) |
| Alvarez-Sanchez (2018) (ID:52947508) | Does the Mexican sugar-sweetened beverage tax have a signaling effect? ENSANUT 2016 | L Am & Car (Mexico) | U- Economic | National | Regulatory | Effectiveness | Quantitative (post CS) | Humans (behaviour) | Place Gender/sex SES Age |
| An (2018) (ID:52946297) | State Laws Governing Competitive Foods and Beverages Sold in Schools and Childhood Obesity among Children with Special Healthcare Needs, 2007-2016 | N Am (USA) | O- Settings U- Economic | State | Regulatory | Effectiveness | Quantitative (post-post CS) | Humans (health) | Disability |
| Andreyeva (2018) (ID:52947809) | Center-Reported Adherence to Nutrition Standards of the Child and Adult Care Food Program | N Am (USA) | O- Settings | National State | Volunt (public/non-profit) Regulatory | Effectiveness  Implementation | Quantitative (post CS) | Humans (food envt) | (none) |
| Arcand (2014) (ID:52950160) | Trans Fatty acids in the Canadian food supply: An updated analysis | N Am (Canada) | I- Reformulat | National | Volunt (public/non-profit) | Effectiveness | Quantitative (post-post CS) | Non-hum (food envt) | Age |
| Au (2020) (ID:52963602) | Post-Healthy, Hunger-Free Kids Act adherence to select school nutrition standards by region and poverty level: The Healthy Communities Study. | N Am (USA) | O- Settings | National | Regulatory | Effectiveness | Quantitative (post CS) | Humans (food envt) Non-hum (food envt) | Place Race Education |
| Azeredo (2020) (ID:52946734) | Are laws restricting soft drinks sales in Brazilian schools able to lower their availability? | L Am & Car (Brazil) | O- Settings | State | Regulatory | Effectiveness | Quantitative (post CS) | Humans (food envt, behaviour) | SES Education |
| Bablani (2020) (ID:52956622) | The impact of voluntary front-of-pack nutrition labelling on packaged food reformulation: A difference-in-differences analysis of the Australasian Health Star Rating scheme. | East As & Pac (Australia, N Zealand) | N- Labelling | International | Volunt (public/non-profit) | Effectiveness | Quantitative (pre-post CS) | Non-hum (food envt) | (none) |
| Bae (2012) (ID:52954270) | Changes in dietary behavior among adolescents and their association with government nutrition policies in Korea, 2005-2009. | East As & Pac (Rep Korea) | O- Settings | National | Mixed | Effectiveness | Quantitative (pre-post CS) | Humans (behaviour) | Gender/sex SES Place Education |
| Bandy (2020) (ID:52946948) | Reductions in sugar sales from soft drinks in the UK from 2015 to 2018 | Euro (UK) | U- Economic | National | Regulatory | Effectiveness | Quantitative (pre-post CS) | Non-hum (food envt) | (none) |
| Barrientos-Gutierrez (2017) (ID:52948204) | Expected population weight and diabetes impact of the 1-peso-per-litre tax to sugar sweetened beverages in Mexico | L Am & Car (Mexico) | U- Economic | National | Regulatory | Effectiveness | Quantitative (M/S) | Humans (health, behaviour) | Gender/sex SES Age |
| Bartfeld (2020) (ID:52946189) | Universal Access to Free School Meals through the Community Eligibility Provision Is Associated with Better Attendance for Low-Income Elementary School Students in Wisconsin | N Am (USA) | O- Settings | National | Volunt (public/non-profit) | Effectiveness | Quantitative (pre-post CS) | Humans (behaviour) | Gender/sex SES |
| Basto-Abreu (2019) (ID:52936476) | Cost-Effectiveness Of The Sugar-Sweetened Beverage Excise Tax In Mexico | L Am & Car (Mexico) | U- Economic | National | Regulatory | Cost-effectiveness | Quantitative (M/S) | N/A | N/A |
| Batis (2016) (ID:52948994) | First-Year Evaluation of Mexico's Tax on (none)ssential Energy-Dense Foods: An Observational Study | L Am & Car (Mexico) | U- Economic | National | Regulatory | Effectiveness | Quantitative (pre-post FU/TS) | Humans (behaviour) | SES |
| BBB National Programs (2020) (ID:62052394) | Annual Report 2019. Children's Food and Beverage Advertising Initiative and Children's Confection Advertising Initiative. | N Am (USA) | R- Advertising | National | Volunt (private) | Effectiveness | Quantitative (post CS) | Humans (food envt) Non-hum (food envt) | (none) |
| Beckelman (2020) (ID:52963601) | Encouraging adults to Choose Healthy Now: A Hawai'i convenience store intervention. | N Am (USA) | S- Retail & catering | State | Voluntary (PPP) | Effectiveness | Quantitative (post CS) | Humans (behaviour) | Gender/sex Race Age |
| Beets (2015) (ID:52949750) | Salty or sweet? Nutritional quality, consumption, and cost of snacks served in afterschool programs | N Am (USA) | O- Settings | National | Volunt (public/non-profit) | Effectiveness | Quantitative (post CS) | Humans (behaviour) Non-hum (food envt) | (none) |
| Benjamin (2016) (ID:52949150) | Comparative Evaluation of a South Carolina Policy to Improve Nutrition in Child Care | N Am (USA) | O- Settings | State | Regulatory | Effectiveness | Quantitative (pre-post CS) | Non-hum (food envt) | (none) |
| Berardi (2016) (ID:52944810) | The Impact of a 'Soda Tax' on Prices: Evidence from French Micro Data | Euro (France) | U- Economic | National | Regulatory | Effectiveness | Quantitative (pre-post FU/TS) | Non-hum (food envt) | (none) |
| Bernhardt (2013) (ID:52950570) | How Television Fast Food Marketing Aimed at Children Compares with Adult Advertisements | N Am (USA) | R- Advertising | National | Volunt (private) | Effectiveness | Quantitative (post CS) | Non-hum (food envt) | Age |
| Berning (2013) (ID:52945148) | Advertising Soft Drinks to Children: Are Voluntary Restrictions Effective? | N Am (USA) | R- Advertising | National | Volunt (private) | Effectiveness | Quantitative (pre-post FU/TS) | Non-hum (food envt) | Age |
| Berning (2014) (ID:52945027) | An Evaluation of Government and Industry Proposed Restrictions on Television Advertising of Breakfast Cereals to Children | N Am (USA) | R- Advertising | National | Volunt (private) | Effectiveness | Quantitative (post-post FU/TS) | Humans (behaviour) Non-hum (food envt) | Age |
| Bertin (2011) (ID:52951923) | Schools meals in French secondary state schools: Compliance to national recommendations and schools catering patterns | Euro (France) | O- Settings | National | Volunt (public/non-profit) | Effectiveness | Quantitative (post CS) | Humans (food envt) Non-hum (food envt) | Education |
| Bhavani (2012) (ID:59697521) | An evaluation of the UK Food Standards Agency's salt campaing | Euro (UK) | I- Reformulat | National | Volunt (public/non-profit) | Effectiveness | Quantitative (pre-post CS) | Humans (behaviour) | Gender/sex Education SES Age |
| Biro (2015) (ID:52944973) | Did the Junk Food Tax Make the Hungarians Eat Healthier? | Euro (Hungary) | U- Economic | National | Regulatory | Effectiveness | Quantitative (pre-post FU/TS) | Humans (behaviour) | SES |
| Bleich (2015) (ID:52935555) | Restaurants With Calories Displayed On Menus Had Lower Calorie Counts Compared To Restaurants Without Such Labels | N Am (USA) | N- Labelling | National | Volunt (private) | Effectiveness | Quantitative (post-post CS) | Non-hum (food envt) | (none) |
| Bleich (2017) (ID:52948210) | Calorie changes in large chain restaurants from 2008 to 2015 | N Am (USA) | N- Labelling | National | Regulatory | Effectiveness | Quantitative (pre-pre CS) | Non-hum (food envt) | Age |
| Bodker (2015) (ID:52949462) | The Danish fat tax-Effects on consumption patterns and risk of ischaemic heart disease | Euro (Denmark) | U- Economic | National | Regulatory | Effectiveness | Quantitative (M/S) | Humans (health, behaviour) | (none) |
| Boyland (2011) (ID:52951912) | The extent of food advertising to children on UK television in 2008 | Euro (UK) | R- Advertising | National | Regulatory | Effectiveness | Quantitative (post-post CS) | Non-hum (food envt) | Age |
| Brookman (2013) (ID:52954536) | Online marketing of food products to children: the effects of national consumer policies in high-income countries | N Am (USA) Euro (France, Spain) | R- Advertising | National | Regulatory Volunt (private) | Effectiveness | Quantitative (post CS) | Non-hum (food envt) | (none) |
| Brownbill (2019) (ID:52947305) | Health Star Ratings: What's on the labels of Australian beverages? | East As & Pac (Australia) | N- Labelling | International | Volunt (public/non-profit) | Effectiveness | Quantitative (post CS) | Non-hum (food envt) | (none) |
| Buckton (2018) (ID:52947557) | The palatability of sugar-sweetened beverage taxation: A content analysis of newspaper coverage of the UK sugar debate | Euro (UK) | U- Economic | National | Regulatory | News coverage | Quantitative (post-post CS) | N/A | N/A |
| Busse (2018) (ID:52947637) | Self-regulation of the Peruvian food industry: health message cues in the context of food and beverage advertisements | L Am & Car (Peru) | R- Advertising | National | Volunt (private) | Effectiveness | Quantitative (post CS) | Non-hum (food envt) | (none) |
| Campos (2016) (ID:72580267) | Analysis of food advertising to children on Spanish television: probing exposure to television marketing | Euro (Spain) | R- Advertising | National | Regulatory | Effectiveness | Quantitative (post CS) | Non-hum (food envt) | Age |
| Campos (2020) (ID:52956632) | The Nutritional Profile of Food Advertising for School-Aged Children via Television: A Longitudinal Approach. | Euro (Spain) | R- Advertising | National | Volunt (private) | Effectiveness | Quantitative (post-post CS) | Non-hum (food envt) | (none) |
| Campos-Vazquez (2019) (ID:52935630) | Pass-through and competition: the impact of soft drink taxes as seen through Mexican supermarkets | L Am & Car (Mexico) | U- Economic | National | Regulatory | Effectiveness | Quantitative (pre-post FU/TS) | Non-hum (food envt) | (none) |
| Capacci (2018) (ID:59683651) | Breaking habits: the effect of the french vending machine ban on school snacking and sugar intakes | Euro (France) | O- Settings | National | Regulatory | Effectiveness | Quantitative (pre-post FU/TS) | Humans (behaviour) | Age |
| Capacci (2019) (ID:52946447) | The impact of the French soda tax on prices and purchases. An ex post evaluation | Euro (France) | U- Economic | National | Regulatory | Effectiveness | Quantitative (pre-post FU/TS) | Humans (behaviour) Non-hum (food envt) | (none) |
| Caro (2018) (ID:52947486) | Chile's 2014 sugar-sweetened beverage tax and changes in prices and purchases of sugar-sweetened beverages: An observational study in an urban environment | L Am & Car (Chile) | U- Economic | National | Regulatory | Effectiveness | Quantitative (pre-post FU/TS) | Humans (food envt) | SES |
| Carter (2013) (ID:52950622) | An independent audit of the Australian food industry's voluntary front-of-pack nutrition labelling scheme for energy-dense nutrition-poor foods | East As & Pac (Australia) | N- Labelling | National | Volunt (private) | Effectiveness | Quantitative (post CS) | Non-hum (food envt) | Age |
| Choi (2018) (ID:52947690) | Korean Adolescents' Energy Intake of Selected Foods by Eating Place from 1998 to 2012 During Implementation of Two National School Nutrition Policies | East As & Pac (Rep Korea) | O- Settings | National | Mixed | Effectiveness | Quantitative (pre-post CS) | Humans (behaviour) | (none) |
| Chriqui (2013) (ID:52950697) | Association between district and state policies and US public elementary school competitive food and beverage environments | N Am (USA) | O- Settings | State National | Regulatory | Effectiveness | Quantitative (post CS) | Humans (food envt) | Place Race SES Education |
| Chriqui (2020) (ID:52956938) | The harmonizing effect of Smart Snacks on the association between state snack laws and high school students' fruit and vegetable consumption, United States-2005-2017. | N Am (USA) | O- Settings | National State | Regulatory | Effectiveness | Quantitative (post CS) | Humans (behaviour) | (none) |
| Christoforou (2013) (ID:52950894) | Changes in the sodium content of australian ready meals between 2008 and 2011 | East As & Pac (Australia) | I- Reformulat | National | Volunt (public/non-profit) Voluntary (PPP) | Effectiveness | Quantitative (pre-post CS) | Non-hum (food envt) | (none) |
| Chu (2020) (ID:52946765) | The sugar content of children's and lunchbox beverages sold in the UK before and after the soft drink industry levy | Euro (UK) | U- Economic | National | Regulatory | Effectiveness | Quantitative (pre-post CS) | Non-hum (food envt) | (none) |
| Cleveland (2018) (ID:52936269) | Compliance in 2017 With Federal Calorie Labeling in 90 Chain Restaurants and 10 Retail Food Outlets Prior to Required Implementation | N Am (USA) | N- Labelling | National | Regulatory | Effectiveness | Quantitative (post CS) | Humans (food envt) Non-hum (food envt) | (none) |
| Cleveland (2020) (ID:52946755) | Federal calorie labelling compliance at US chain restaurants | N Am (USA) | N- Labelling | National | Regulatory | Effectiveness | Quantitative (post CS) | Non-hum (food envt) | (none) |
| Cohen (2016) (ID:52948879) | Healthier Standards for School Meals and Snacks: Impact on School Food Revenues and Lunch Participation Rates | N Am (USA) | O- Settings | National State | Regulatory | Effectiveness | Quantitative (post CS) | Non-hum (other) | SES |
| Colantuoni (2015) (ID:52935515) | The impact of dosa sales taxes on consumption: evidence from scanner data | N Am (USA) | U- Economic | State | Regulatory | Effectiveness | Quantitative (pre-post FU/TS) | Humans (behaviour) | (none) |
| Colchero (2015) (ID:52969888) | Changes in Prices After an Excise Tax to Sweetened Sugar Beverages Was Implemented in Mexico: Evidence from Urban Areas | L Am & Car (Mexico) | U- Economic | National | Regulatory | Effectiveness | Quantitative (pre-post FU/TS) | Humans (behaviour) Non-hum (food envt) | (none) |
| Colchero (2016) (ID:52949016) | Beverages sales in Mexico before and after implementation of a sugar sweetened beverage tax | L Am & Car (Mexico) | U- Economic | National | Regulatory | Effectiveness | Quantitative (pre-post FU/TS) | Humans (behaviour) | (none) |
| Colchero (2016) (ID:52969736) | Beverage purchases from stores in Mexico under the excise tax on sugar sweetened beverages: observational study | L Am & Car (Mexico) | U- Economic | National | Regulatory | Effectiveness | Quantitative (pre-post FU/TS) | Humans (behaviour) | SES |
| Colchero (2017) (ID:52936200) | In Mexico, Evidence Of Sustained Consumer Response Two Years After Implementing A Sugar-Sweetened Beverage Tax | L Am & Car (Mexico) | U- Economic | National | Regulatory | Effectiveness | Quantitative (pre-post FU/TS) | Humans (behaviour) | SES |
| Colchero (2017) (ID:52948298) | After Mexico implemented a tax, purchases of sugar-sweetened beverages decreased and water increased: Difference by place of residence, household composition, and income level | L Am & Car (Mexico) | U- Economic | National | Regulatory | Effectiveness | Quantitative (pre-post CS) | Humans (behaviour) | Place SES Age |
| Colchero (2017) (ID:52948474) | Changes in prices of taxed sugar-sweetened beverages and (none)ssential energy dense food in rural and semi-rural areas in Mexico | L Am & Car (Mexico) | U- Economic | National | Regulatory | Effectiveness | Quantitative (pre-post CS) | Non-hum (food envt) | (none) |
| Cornelsen (2017) (ID:52940756) | Change in non-alcoholic beverage sales following a 10-pence levy on sugar-sweetened beverages within a national chain of restaurants in the UK: interrupted time series analysis of a natural experiment | Euro (UK) | U- Economic | National | Volunt (private) | Effectiveness | Quantitative (pre-post FU/TS) | Humans (behaviour) | Age |
| Correa (2020) (ID:52935432) | Food Advertising on Television Before and After a National Unhealthy Food Marketing Regulation in Chile, 2016-2017 | L Am & Car (Chile) | R- Advertising | National | Regulatory | Effectiveness | Quantitative (post CS) | Non-hum (food envt) | Age |
| Craig (2020) (ID:52935629) | Do menu-labelling laws translate into results? The disparate impacts on population obesity and diabetes | N Am (USA) | N- Labelling | State | Regulatory | Effectiveness | Quantitative (pre-post CS) | Humans (health) | (none) |
| Cranney (2020) (ID:52942720) | Implementation and acceptance of a state-wide policy to remove sugar-sweetened beverages in hospitals in New South Wales, Australia | East As & Pac (Australia) | O- Settings | State | Regulatory | Effectiveness | Quantitative (post-post CS) | Non-hum (food envt) | (none) |
| Cuadrado (2020) (ID:52947011) | Effects of a sugar-sweetened beverage tax on prices and affordability of soft drinks in Chile: A time series analysis | L Am & Car (Chile) | U- Economic | National | Regulatory | Effectiveness | Quantitative (pre-post FU/TS) | Non-hum (food envt) | (none) |
| Curtis (2016) (ID:52948939) | US Food Industry Progress During the National Salt Reduction Initiative: 2009-2014 | N Am (USA) | I- Reformulat | National | Volunt (public/non-profit) | Effectiveness | Quantitative (post-post CS) | Non-hum (food envt) | (none) |
| Cushman (2012) (ID:52964897) | The impact of short-term food regulations in New Zealand schools. | East As & Pac (N Zealand) | O- Settings | National | Regulatory | Effectiveness | Quantitative (post CS) | Humans (policy characts) | Education |
| Datar (2017) (ID:52948261) | The Effect of State Competitive Food and Beverage Regulations on Childhood Overweight and Obesity | N Am (USA) | O- Settings | State | Regulatory | Effectiveness | Quantitative (post CS) | Humans (health) | (none) |
| de Albuquerque (2016) (ID:52969379) | Evaluation of the adequacy of infant formula labeling | L Am & Car (Brazil) | R- Advertising | National | Regulatory | Effectiveness | Quantitative (post CS) | Non-hum (food envt) | (none) |
| Dhar (2011) (ID:59693518) | Fast-Food Consumption and the Ban on Advertising Targeting Children: The Quebec Experience | N Am (Canada) | R- Advertising | State | Regulatory | Effectiveness | Quantitative (pre-post CS) | Humans (behaviour) | Age Race |
| Dick (2012) (ID:52961443) | Evaluation of implementation of a healthy food and drink supply strategy throughout the whole school environment in Queensland state schools, Australia. | East As & Pac (Australia) | O- Settings | State | Regulatory | Implementation | Quantitative (post CS) | N/A | N/A |
| Dickinson (2013) (ID:52950572) | The impact of amended controls on the advertising of infant formula in the UK: Findings from a before and after study | Euro (UK) | R- Advertising | National | Regulatory | Effectiveness | Quantitative (pre-post CS) | Non-hum (food envt) | Race Gender/sex Age |
| Dillman (2019) (ID:52946839) | Evaluating the impact of Chile's marketing regulation of unhealthy foods and beverages: pre-school and adolescent children's changes in exposure to food advertising on television | L Am & Car (Chile) | R- Advertising | National | Regulatory | Effectiveness | Quantitative (pre-post FU/TS) | Humans (food envt) | Age |
| Dubuisson (2015) (ID:52949581) | The relationship between school lunch attendance and the food intakes of French schoolchildren aged 3-17 years | Euro (France) | O- Settings | National | Volunt (public/non-profit) | Effectiveness | Quantitative (post CS) | Humans (behaviour) | Education |
| Dunford (2011) (ID:52951752) | Changes in the sodium content of bread in Australia and New Zealand between 2007 and 2010: Implications for policy | East As & Pac (Australia, N Zealand) | I- Reformulat | National | Volunt (public/non-profit) Voluntary (PPP) | Effectiveness | Quantitative (pre-post CS) | Non-hum (food envt) | (none) |
| Ebocskei (2010) (ID:52954150) | Measuring Up: An Evaluation of the BC Trans Fat Initiative | N Am (Canada) | I- Reformulat | State | Regulatory | Implementation Effectiveness | Quantitative (post CS) | Non-hum (food envt, policy characts) | (none) |
| Effertz (2012) (ID:52951471) | Do television food commercials target children in Germany? | Euro (Germany) | R- Advertising | International | Volunt (private) | Effectiveness | Quantitative (pre-post CS) | Non-hum (food envt) | Age |
| Ejlerskov (2018) (ID:52947706) | The nature of UK supermarkets' policies on checkout food and associations with healthfulness and type of food displayed: Cross-sectional study | Euro (UK) | S- Retail & catering | National | Volunt (private) | Effectiveness | Quantitative (pre-post FU/TS) | Non-hum (food envt, policy characts) | (none) |
| Ejlerskov (2018) (ID:52958492) | Supermarket policies on less-healthy food at checkouts: Natural experimental evaluation using interrupted time series analyses of purchases. | Euro (UK) | S- Retail & catering | National | Volunt (private) | Effectiveness | Quantitative (post CS) | Humans (behaviour) | Occupation Age |
| Elorriaga (2017) (ID:52948229) | Collecting evidence to inform salt reduction policies in argentina: Identifying sources of sodium intake in adults from a population-based sample | L Am & Car (Argentina) | I- Reformulat | National | Regulatory | Effectiveness | Quantitative (post CS) | Humans (behaviour, food envt) | Gender/sex Education Age |
| Elshiewy (2018) (ID:52963874) | When back of pack meets front of pack: How salient and simplified nutrition labels affect food sales in supermarkets. | Euro (UK) | N- Labelling | National | Volunt (private) | Effectiveness | Quantitative (pre-post FU/TS) | Humans (behaviour) | (none) |
| Erinosho (2018) (ID:52947498) | The quality of nutrition and physical activity environments of child-care centers across three states in the southern U.S | N Am (USA) | O- Settings | National | Volunt (public/non-profit) | Effectiveness | Quantitative (post CS) | Humans (food envt) | Place |
| Essman (2018) (ID:52947699) | Sugar-sweetened beverage intake among chilean preschoolers and adolescents in 2016: A cross-sectional analysis | L Am & Car (Chile) | U- Economic | National | Regulatory | Effectiveness | Quantitative (pre-post CS) | Humans (behaviour) | Gender/sex Age Education |
| Etile, (2020) (ID:65873488) | Market heterogeneity and the distributional incidence of soft-drink taxes: evidence from France | Euro (France) | U- Economic | National | Regulatory | Effectiveness | Quantitative (pre-post FU/TS) | Humans (behaviour) | SES |
| Eyler (2020) (ID:52956933) | Adherence to Updated Childcare Nutrition Regulations in Colorado, United States. | N Am (USA) | O- Settings | National State | Volunt (public/non-profit) | Effectiveness  Implementation | Quantitative (post CS) | Humans (food envt) | Place Age |
| Eyles (2013) (ID:52950543) | Impact of the UK voluntary sodium reduction targets on the sodium content of processed foods from 2006 to 2011: Analysis of household consumer panel data | Euro (UK) | I- Reformulat | National | Volunt (public/non-profit) | Effectiveness | Quantitative (pre-post FU/TS) | Humans (behaviour) Non-hum (food envt) | (none) |
| Fernandes (2013) (ID:52950874) | A National Evaluation of the Impact of State Policies on Competitive Foods in Schools | N Am (USA) | O- Settings | State | Regulatory | Effectiveness | Quantitative (post-post FU/TS) | Humans (food envt) | Race Education SES |
| Fletcher (2010) (ID:52935372) | Taxing Soft Drinks And Restricting Access To Vending Machines To Curb Child Obesity | N Am (USA) | U- Economic O- Settings | State | Regulatory | Effectiveness | Quantitative (post-post FU/TS) | Humans (behaviour, health, food envt) | (none) |
| Fletcher (2010) (ID:52945446) | The Effects of Soft Drink Taxes on Child and Adolescent Consumption and Weight Outcomes | N Am (USA) | U- Economic | State | Regulatory | Effectiveness | Quantitative (post CS) | Humans (behaviour, health) | (none) |
| Fletcher (2010) (ID:52945516) | Can Soft Drink Taxes Reduce Population Weight? | N Am (USA) | U- Economic | State | Regulatory | Effectiveness | Quantitative (post-post CS) | Humans (health) | Race Gender/sex SES Age Education |
| Fletcher (2015) (ID:52944972) | Non-linear Effects of Soda Taxes on Consumption and Weight Outcomes | N Am (USA) | U- Economic | State | Regulatory | Effectiveness | Quantitative (pre-post CS) | Humans (behaviour, health) | (none) |
| Franco-Arellano (2020) (ID:52946461) | Progress towards eliminating industrially produced trans-fatty acids in the Canadian marketplace, 2013-2017 | N Am (Canada) | N- Labelling I- Reformulat | National | Mixed | Effectiveness | Quantitative (post-post CS) | Non-hum (food envt) | Age |
| Freedman (2018) (ID:52935896) | Supply-side subsidies to improve food access and dietary outcomes: Evidence from the New Markets Tax Credit | N Am (USA) | S- Retail & catering | National | Volunt (public/non-profit) | Effectiveness | Quantitative (pre-post FU/TS) | Humans (behaviour) Non-hum (food envt) | SES |
| Gabrielyan (2017) (ID:52948522) | Who's adopting the smarter lunchroom approach? Individual characteristics of innovative food service directors | N Am (USA) | O- Settings | National | Volunt (public/non-profit) | Implementation | Quantitative (post CS) | N/A | N/A |
| Gamboa-Gamboa (2019) (ID:52958144) | Nutritional Content According to the Presence of Front of Package Marketing Strategies: The Case of Ultra-Processed Snack Food Products Purchased in Costa Rica. | L Am & Car (Costa Rica) | N- Labelling | International | Volunt (public/non-profit) | Effectiveness | Quantitative (post CS) | Non-hum (food envt) | (none) |
| Gebreab (2015) (ID:52949704) | Geographic variations in cardiovascular health in the United States: contributions of state- and individual-level factors | N Am (USA) | U- Economic | State | Regulatory | Effectiveness | Quantitative (post CS) | Humans (health) | (none) |
| Ghosh (2018) (ID:52968461) | The Political Economy of Soda Taxation | N Am (USA) | U- Economic | State | Regulatory | Implementation | Quantitative (post CS) | N/A | N/A |
| Godin (2018) (ID:52947698) | Examining changes in school vending machine beverage availability and sugar-sweetened beverage intake among Canadian adolescents participating in the COMPASS study: A longitudinal assessment of provincial school nutrition policy compliance and effectiveness | N Am (Canada) | O- Settings | State | Regulatory Volunt (public/non-profit) | Effectiveness | Quantitative (post-post FU/TS) | Humans (behaviour) Non-hum (food envt) | Education |
| Godin (2019) (ID:52946575) | Examining associations between school food envt and sugar-sweetened beverage consumption among Canadian secondary-school students in the COMPASS study | N Am (Canada) | O- Settings | State | Volunt (public/non-profit) Regulatory | Effectiveness | Quantitative (post CS) | Humans (behaviour) Non-hum (food envt) | Race Gender/sex SES Place Education |
| Goiana-Da-Silva (2020) (ID:52946366) | Projected impact of the Portuguese sugar-sweetened beverage tax on obesity incidence across different age groups: A modelling study | Euro (Portugal) | U- Economic | National | Regulatory | Effectiveness | Quantitative (M/S) | Humans (behaviour, health) | Age |
| Goncalves (2020) (ID:52946144) | Brown sugar, how come you taste so good? The impact of a soda tax on prices and consumption | Euro (Portugal) | U- Economic | National | Regulatory | Effectiveness | Quantitative (pre-post FU/TS) | Humans (behaviour, food envt) | Place |
| Goodman (2011) (ID:52961718) | Use of nutritional information in Canada: national trends between 2004 and 2008. | N Am (Canada) | N- Labelling | National | Regulatory | Effectiveness | Quantitative (pre-post CS) | Humans (behaviour) | Gender/sex Education SES Age |
| Goodman (2018) (ID:52947719) | A quasi-experimental study of a mandatory calorie-labelling policy in restaurants: Impact on use of nutrition information among youth and young adults in Canada | N Am (Canada) | N- Labelling | State | Regulatory Volunt (public/non-profit) | Effectiveness | Quantitative (pre-post FU/TS) | Humans (behaviour, other) | Gender/sex SES Age Race |
| Gorski (2016) (ID:52949144) | Impact of Nutrition Standards on Competitive Food Quality in Massachusetts Middle and High Schools | N Am (USA) | O- Settings | State | Regulatory | Effectiveness | Quantitative (post-post CS) | Non-hum (food envt) | Race SES Education |
| Grabovac (2018) (ID:52935366) | Impact of Austria's 2009 trans fatty acids regulation on all-cause, cardiovascular and coronary heart disease mortality | Euro (Austria) | I- Reformulat | National | Regulatory | Effectiveness | Quantitative (pre-post CS) | Humans (health) | Gender/sex |
| Grafenauer (2018) (ID:52968034) | An Audit of Australian Bread with a Focus on Loaf Breads and Whole Grain | East As & Pac (Australia) | N- Labelling | International | Volunt (public/non-profit) | Effectiveness | Quantitative (pre-post CS) | Non-hum (food envt) | (none) |
| Gregoric (2015) (ID:59693026) | School nutrition guidelines: overview of the implementation and evaluation | Euro (Slovenia) | O- Settings | National | Regulatory | Effectiveness | Quantitative (post CS) | Humans (policy characts) Non-hum (food envt) | Place Education |
| Grogger (2017) (ID:52944734) | Soda Taxes and the Prices of Sodas and Other Drinks: Evidence from Mexico | L Am & Car (Mexico) | U- Economic | National | Regulatory | Effectiveness | Quantitative (pre-post FU/TS) | Non-hum (food envt) | (none) |
| Guerrero-Lopez (2017) (ID:52948089) | Employment changes associated with the introduction of taxes on sugar-sweetened beverages and (none)ssential energy-dense food in Mexico | L Am & Car (Mexico) | U- Economic | National | Regulatory | Effectiveness | Quantitative (pre-post FU/TS) | Humans (other) | Occupation |
| Haroun (2011) (ID:52961772) | Nutrient-based standards for school lunches complement food-based standards and improve pupils' nutrient intake profile. | Euro (UK) | O- Settings | State | Regulatory | Effectiveness | Quantitative (post CS) | Humans (food envt) | (none) |
| Harpainter (2020) (ID:52946518) | Voluntary kids' meal beverage standards: Are they sufficient to ensure healthier restaurant practices and consumer choices? | N Am (USA) | S- Retail & catering | National | Volunt (private) | Effectiveness | Quantitative (post CS) | Humans (food envt) Non-hum (food envt) | (none) |
| Harris (2013) (ID:52950553) | Redefining "child-directed advertising" to reduce unhealthy television food advertising | N Am (USA) | R- Advertising | National | Volunt (private) | Effectiveness | Quantitative (post CS) | Non-hum (food envt) | Age |
| Harris (2015) (ID:52949421) | Sweet promises: Candy advertising to children and implications for industry self-regulation | N Am (USA) | R- Advertising | National | Volunt (private) | Effectiveness | Quantitative (pre-post CS) | Non-hum (food envt) | Age |
| Harris (2017) (ID:68341301) | F.A.C.T.S. 2017. Food industry self-regulation after 10 years: Progress and opportunities to improve food advertising to children | N Am (USA) | R- Advertising | National | Volunt (private) | Effectiveness | Quantitative (post-post CS) | Humans (behaviour, food envt) Non-hum (food envt, other) | Race Age |
| Harris (2018) (ID:52947729) | Food and beverage TV advertising to young children: Measuring exposure and potential impact | N Am (USA) | R- Advertising | National | Volunt (private) | Effectiveness | Quantitative (post CS) | Non-hum (food envt) | Age |
| Hashem (2019) (ID:52935690) | Labelling changes in response to a tax on sugar-sweetened beverages, United Kingdom of Great Britain and Northern Ireland | Euro (UK) | U- Economic | National | Regulatory | Effectiveness | Quantitative (pre-post CS) | Non-hum (food envt) | (none) |
| Health Canada (2018) (ID:59687222) | Sodium Reduction in Processed Foods in Canada: An evaluation of Progress toward Voluntary Targets from 2012 to 2016 | N Am (Canada) | I- Reformulat | National | Volunt (public/non-profit) | Effectiveness | Quantitative (post CS) | Non-hum (food envt) | (none) |
| Hebden (2011) (ID:52951864) | Advertising of fast food to children on Australian television: The impact of industry self-regulation | East As & Pac (Australia) | R- Advertising | National | Volunt (private) | Effectiveness | Quantitative (pre-post CS) | Non-hum (food envt) | (none) |
| Hennessy (2014) (ID:52950233) | State-level school competitive food and beverage laws are associated with children's weight status | N Am (USA) | O- Settings | State | Regulatory | Effectiveness | Quantitative (post CS) | Humans (health) | (none) |
| Hernandez-F (2019) (ID:52947109) | Reduction in purchases of energy-dense nutrient-poor foods in Mexico associated with the introduction of a tax in 2014 | L Am & Car (Mexico) | U- Economic | National | Regulatory | Effectiveness | Quantitative (pre-post FU/TS) | Humans (behaviour) | Place Education SES Age |
| Hobin (2017) (ID:52942049) | Consumers Response to an On-Shelf Nutrition Labelling System in Supermarkets: evidence to Inform Policy and Practice | N Am (Canada) | N- Labelling | International | Volunt (private) | Effectiveness | Quantitative (pre-post CS) | Humans (behaviour) | (none) |
| Hoelscher (2010) (ID:52965248) | Changes in the regional prevalence of child obesity in 4th, 8th, and 11th grade students in Texas from 2000-2002 to 2004-2005. | N Am (USA) | Wide range | State | Regulatory | Effectiveness | Quantitative (pre-post CS) | Humans (health) | Age |
| Hoepner (2014) (ID:52970882) | The Level of Compliance with the International Code of Marketing of Breast-Milk Substitutes: Does it Matter to Stock Markets? | Unclear/ Not reported | R- Advertising | International | Volunt (public/non-profit) | Effectiveness | Quantitative (pre-post CS) | Non-hum (other) | (none) |
| Hoffer (2020) (ID:52935448) | Expenditure Effects from the 2010 Washington Soda Tax | N Am (USA) | U- Economic | State | Regulatory | Effectiveness | Quantitative (post CS) | Humans (behaviour) | (none) |
| Honorio (2020) (ID:52965873) | Consumption of school meals provided by PNAE among brazilian public school adolescents | L Am & Car (Brazil) | O- Settings | National | Regulatory | Effectiveness | Quantitative (post CS) | Humans (behaviour) | Gender/sex Race Occupation SES Age |
| Hooker (2014) (ID:52945001) | Trans-border Reformulation: US and Canadian Experiences with Trans Fat | N Am (Canada, USA) | I- Reformulat N- Labelling | National | Regulatory Mixed | Effectiveness | Quantitative (post-post CS) | Non-hum (food envt) | (none) |
| Hu (2020) (ID:52965823) | How do parents respond to regulation of sugary drinks in child care? Evidence from California | N Am (USA) | O- Settings | State | Regulatory | Effectiveness | Quantitative (pre-post FU/TS) | Humans (behaviour) | SES |
| Huang (2012) (ID:59704561) | Does limited access at school result in compensation at home? The effect of soft drink bans in schools on purchase patterns outside of schools | N Am (USA) | O- Settings | National State | Regulatory | Effectiveness | Quantitative (pre-post FU/TS) | Humans (behaviour, food envt) Non-hum (food envt) | Race Occupation Gender/sex Education SES Age |
| Huang (2013) (ID:52964732) | Buy what is advertised on television? Evidence from bans on child-directed food advertising. | N Am (USA) | R- Advertising | National | Volunt (private) | Effectiveness | Quantitative (pre-post FU/TS) | Humans (behaviour) Non-hum (food envt) | Age |
| Huang (2020) (ID:52966219) | Bus Stops Near Schools Advertising Junk Food and Sugary Drinks | East As & Pac (N Zealand) | R- Advertising | National | Volunt (private) | Effectiveness | Quantitative (post CS) | Non-hum (food envt) | Place Education |
| Hurwitz (2017) (ID:52948477) | Food Marketing to Children Online: A Content Analysis of Food Company Websites | N Am (USA) | R- Advertising | National | Volunt (private) | Effectiveness | Quantitative (post CS) | Non-hum (food envt) | Age |
| Hurwitz (2019) (ID:52936763) | Crowd pleasers: media characters in food company websites and apps for children | N Am (USA) | R- Advertising | National | Volunt (private) | Effectiveness | Quantitative (post CS) | Non-hum (food envt) | Race Gender/sex Age |
| Hutchinson (2018) (ID:52947899) | Comparison of high and low trans-fatty acid consumers: analyses of UK National Diet and Nutrition Surveys before and after product reformulation | Euro (UK) | I- Reformulat | State | Voluntary (PPP) | Effectiveness | Quantitative (pre-post CS) | Humans (behaviour) | Race Occupation Gender/sex Education SES Age Disability |
| Jahn (2018) (ID:52968245) | Product reformulation and nutritional improvements after new competitive food standards in schools | N Am (USA) | I- Reformulat | State | Regulatory | Effectiveness | Quantitative (pre-post CS) | Non-hum (food envt) | (none) |
| Jaichuen (2019) (ID:52958467) | Food Marketing in Facebook to Thai Children and Youth: An Assessment of the Efficacy of Thai Regulations. | East As & Pac (Thailand) | R- Advertising | National | Mixed | Effectiveness | Quantitative (post CS) | Non-hum (food envt) | (none) |
| Jensen (2013) (ID:52945157) | The Danish Tax on Saturated Fat--Short Run Effects on Consumption, Substitution Patterns and Consumer Prices of Fats | Euro (Denmark) | U- Economic | National | Regulatory | Effectiveness | Quantitative (pre-post FU/TS) | Humans (behaviour) | Place |
| Jensen (2015) (ID:52949047) | Effects of the Danish saturated fat tax on the demand for meat and dairy products | Euro (Denmark) | U- Economic | National | Regulatory | Effectiveness | Quantitative (pre-post FU/TS) | Humans (behaviour) | (none) |
| Jensen (2020) (ID:61116918) | Examining Chile's unique food marketing policy: TV advertising and dietary intake in preschool children, a pre- and post- policy study | L Am & Car (Chile) | R- Advertising | National | Regulatory | Effectiveness | Quantitative (pre-post FU/TS) | Humans (behaviour) | (none) |
| Jones (2018) (ID:52947701) | Uptake of australia's health star rating system | East As & Pac (Australia) | N- Labelling | International | Volunt (public/non-profit) | Effectiveness | Quantitative (pre-post CS) | Non-hum (food envt) | (none) |
| Jumpertz (2013) (ID:52950641) | Food label accuracy of common snack foods | N Am (USA) | N- Labelling | National | Regulatory | Effectiveness | Quantitative (post CS) | Non-hum (food envt) | (none) |
| Juniusdottir (2018) (ID:52947760) | Composition of School Meals in Sweden, Finland, and Iceland: Official Guidelines and Comparison With Practice and Availability | Euro (Iceland, Finland, Sweden) | O- Settings | National | Regulatory | Effectiveness | Quantitative (post CS) | Non-hum (food envt) | (none) |
| Kakisu (2018) (ID:52947865) | Analysis of the reduction of trans-fatty-acid levels in the foods of Argentina | L Am & Car (Argentina) | I- Reformulat | National | Regulatory | Effectiveness | Quantitative (pre-post CS) | Non-hum (food envt) | (none) |
| Kanter (2019) (ID:52947230) | Anticipatory effects of the implementation of the Chilean Law of Food Labeling and Advertising on food and beverage product reformulation | L Am & Car (Chile) | N- Labelling | National | Regulatory | Effectiveness | Quantitative (pre-pre CS) | Non-hum (food envt) | (none) |
| Kelly (2016) (ID:52969683) | Children's exposure to food advertising on free-to-air television: an Asia-Pacific perspective | East As & Pac (Rep Korea) | R- Advertising | National | Regulatory | Effectiveness | Quantitative (post CS) | Non-hum (food envt) | Age |
| Kelly (2019) (ID:52947120) | Global benchmarking of children's exposure to television advertising of unhealthy foods and beverages across 22 countries | N Am (Canada) Euro (Slovenia, Spain, UK) L Am & Car (Colombia, Mexico) East As & Pac (Australia, Malaysia, N Zealand, Thailand) Sub-Sah Afr (South Africa) | R- Advertising | National | Regulatory Volunt (private) | Effectiveness | Quantitative (post CS) | Non-hum (food envt) | Age |
| Kim (2013) (ID:52950954) | Restriction of television food advertising in South Korea: impact on advertising of food companies | East As & Pac (Rep Korea) | R- Advertising | National | Regulatory | Effectiveness | Quantitative (pre-post CS) | Non-hum (food envt) | (none) |
| Kim (2016) (ID:52948880) | Is nutritional labeling associated with individual health? the effects of labeling-based awareness on dyslipidemia risk in a South Korean population | East As & Pac (Rep Korea) | N- Labelling | National | Regulatory | Effectiveness | Quantitative (post CS) | Humans (behaviour) | Gender/sex Education Age Occupation SES |
| Kim (2019) (ID:52946577) | A comparison of the Health Star Rating and nutrient profiles of branded and generic food products in Sydney supermarkets, Australia | East As & Pac (Australia) | N- Labelling | International | Volunt (public/non-profit) | Effectiveness | Quantitative (post CS) | Non-hum (food envt) | (none) |
| King (2011) (ID:52951937) | Industry self regulation of television food advertising: Responsible or responsive? | East As & Pac (Australia) | R- Advertising | National | Volunt (private) | Effectiveness | Quantitative (pre-post CS) | Non-hum (food envt) | Age |
| King (2013) (ID:52950971) | Building the case for independent monitoring of food advertising on Australian television | East As & Pac (Australia) | R- Advertising | National | Volunt (private) | Effectiveness | Quantitative (pre-post CS) | Non-hum (food envt) | Age |
| Kliemann (2014) (ID:52970799) | Reference serving sizes for the Brazilian population: An analysis of processed food labels | L Am & Car (Brazil) | N- Labelling | National | Regulatory | Effectiveness | Quantitative (post CS) | Non-hum (food envt) | (none) |
| Kliemann (2016) (ID:52969416) | Serving size on nutrition labeling for processed foods sold in Brazil: Relationship to energy value | L Am & Car (Brazil) | N- Labelling | National | Regulatory | Effectiveness | Quantitative (post CS) | Non-hum (food envt) | (none) |
| Kok (2017) (ID:52935547) | Accuracy of nutrition labels of pre-packaged foods in Malaysia | East As & Pac (Malaysia) | N- Labelling | National | Mixed | Effectiveness | Quantitative (post CS) | Non-hum (food envt) | (none) |
| Kong (2017) (ID:52948574) | The presence and accuracy of nutritional labelling of pre-packaged foods in Shanghai | East As & Pac (China) | N- Labelling | National | Regulatory | Effectiveness | Quantitative (post CS) | Non-hum (food envt) | (none) |
| Korff (2020) (ID:52946182) | The South African sodium regulation (R214): Does it make provision for processed foods frequently consumed by young children? | Sub-Sah Afr (South Africa) | I- Reformulat | National | Regulatory | Effectiveness | Quantitative (post CS) | Non-hum (food envt) | (none) |
| Kovac (2019) (ID:52935417) | Does the ban on trans-fats improve public health? In search of the optimal policy response | N Am (USA) Euro (Denmark, France, Germany, Netherlands, Switzerland, UK) East As & Pac (Australia) | N- Labelling I- Reformulat | National State | Regulatory Volunt (private) Volunt (public/non-profit) Voluntary (PPP) | Effectiveness | Quantitative (pre-post CS) | Humans (health) | Age |
| Kovic (2018) (ID:52947519) | The impact of junk food marketing regulations on food sales: an ecological study | N Am (Canada, USA) L Am & Car (Brazil, Chile, Colombia, Ecuador, Mexico, Peru) East As & Pac (Australia, Indonesia, Malaysia, Philippines, Rep Korea, Singapore, Thailand) M East & N Afr (Saudi Arabia, United Arab Emirates) South Asia (India) Sub-Sah Afr (South Africa) Euro (Austria, Belgium, Bulgaria, Croatia, Czech Republic, Denmark, Estonia, France, Finland, Germany, Greece, Hungary, Ireland, Italy, Latvia, Lithuania, Netherlands, Norway, Poland, Portugal, Romania, Russia, Serbia, Slovakia, Slovenia, Spain, Sweden, Switzerland, UK) | R- Advertising | National International | Regulatory Volunt (private) Mixed | Effectiveness | Quantitative (pre-post CS) | Humans (behaviour) | Place |
| Kraak (2014) (ID:52945020) | A Q Methodology Study of Stakeholders' Views about Accountability for Promoting Healthy Food Environments in England through the Responsibility Deal Food Network | Euro (UK) | Wide range | State | Voluntary (PPP) | Implementation | Quantitative (post CS) | N/A | N/A |
| Kraemer (2016) (ID:52949172) | Sodium content on processed foods for snacks | L Am & Car (Brazil) | N- Labelling | National | Regulatory | Effectiveness | Quantitative (post CS) | Non-hum (food envt) | (none) |
| Kubik (2010) (ID:52962028) | State but not district nutrition policies are associated with less junk food in vending machines and school stores in US public schools. | N Am (USA) | O- Settings | State | Regulatory Volunt (public/non-profit) | Effectiveness | Quantitative (post CS) | Humans (food envt) | Place Education |
| Kumar (2017) (ID:52936550) | Do labels influence purchase decisions of food products? Study of young consumers of an emerging market | South Asia (India) | N- Labelling | National | Regulatory | Effectiveness | Quantitative (post CS) | Humans (behaviour) | Gender/sex Education SES Age |
| Kunkel (2014) (ID:52964623) | Solution or smokescreen? Evaluating industry self-regulation of televised food marketing to children. | N Am (USA) | R- Advertising | National | Volunt (private) | Effectiveness | Quantitative (pre-post CS) | Non-hum (food envt) | (none) |
| Kunkel (2015) (ID:52949567) | Evaluating Industry Self-Regulation of Food Marketing to Children | N Am (USA) | R- Advertising | National | Volunt (private) | Effectiveness | Quantitative (pre-post CS) | Non-hum (food envt) | (none) |
| Landwehr (2020) (ID:52944424) | Industry Self-Regulation of Food Advertisement to Children: Compliance versus Effectiveness of the EU Pledge | Euro (Germany) | R- Advertising | International | Volunt (private) | Effectiveness | Quantitative (pre-post CS) | Non-hum (food envt) | Age |
| Laverty (2019) (ID:52947294) | Quantifying the impact of the Public Health Responsibility Deal on salt intake, cardiovascular disease and gastric cancer burdens: interrupted time series and microsimulation study | Euro (UK) | I- Reformulat | State | Voluntary (PPP) | Cost-effectiveness | Quantitative (pre-post FU/TS) | N/A | N/A |
| Lavrisa (2020) (ID:52946623) | Regulating children's exposure to food marketing on television: are the restrictions during children's programmes enough? | Euro (Slovenia) | R- Advertising | National | Volunt (private) | Effectiveness | Quantitative (pre-post CS) | Non-hum (food envt) | Age |
| Law (2020) (ID:52944378) | An Analysis of the Stock Market Reaction to the Announcements of the UK Soft Drinks Industry Levy | Euro (UK) | U- Economic | National | Regulatory | Effectiveness | Quantitative (pre-post FU/TS) | Non-hum (other) | (none) |
| Law (2020) (ID:52946792) | The impact of UK soft drinks industry levy on manufacturers' domestic turnover | Euro (UK) | U- Economic | National | Regulatory | Effectiveness | Quantitative (pre-post FU/TS) | Humans (food envt) | (none) |
| Lee (2010) (ID:52952187) | Trans fatty acids content and fatty acid profiles in the selected food products from Korea between 2005 and 2008 | East As & Pac (Rep Korea) | N- Labelling | National | Regulatory | Effectiveness | Quantitative (pre-post CS) | Non-hum (food envt) | (none) |
| Lee (2018) (ID:52963849) | Effect of TV food advertising restriction on food environment for children in South Korea | East As & Pac (Rep Korea) | R- Advertising | National | Regulatory | Effectiveness | Quantitative (post CS) | Humans (food envt) | (none) |
| Lee-Kwan (2014) (ID:52950346) | Restaurant menu labeling use among adults--17 states, 2012 | N Am (USA) | N- Labelling | National | Regulatory | Effectiveness | Quantitative (post CS) | Humans (behaviour) | Place Race Gender/sex Age |
| Lee-Kwan (2016) (ID:52949136) | Factors Associated with Self-Reported Menu-Labeling Usage among US Adults | N Am (USA) | N- Labelling | State | Regulatory | Effectiveness | Quantitative (post CS) | Humans (behaviour) | Race Gender/sex Education SES Age |
| Leite (2020) (ID:52941984) | Nutritional quality of foods and non-alcoholic beverages advertised on Brazilian free-to-air television: a cross-sectional study | L Am & Car (Brazil) | R- Advertising | National | Regulatory | Effectiveness | Quantitative (post CS) | Non-hum (food envt) | (none) |
| Leon-Flandez (2017) (ID:52948139) | Evaluation of compliance with the Spanish Code of self-regulation of food and drinks advertising directed at children under the age of 12 years in Spain, 2012 | Euro (Spain) | R- Advertising | National | Volunt (private) | Effectiveness | Quantitative (post CS) | Non-hum (food envt) | (none) |
| Lessard (2013) (ID:52950886) | Consistency of compliance with nutrition-related regulations among delaware child care centers | N Am (USA) | O- Settings | State | Regulatory | Effectiveness | Quantitative (post CS) | Humans (food envt) | Education |
| Levi (2018) (ID:52947766) | Evaluation of Australian soup manufacturer compliance with national sodium reduction targets | East As & Pac (Australia) | I- Reformulat | National | Voluntary (PPP) | Effectiveness | Quantitative (post-post CS) | Non-hum (food envt) | (none) |
| Lin (2019) (ID:52958049) | Dietary Guidance and New School Meal Standards: Schoolchildren's Whole Grain Consumption Over 1994-2014. | N Am (USA) | O- Settings | National | Regulatory | Effectiveness | Quantitative (pre-post CS) | Humans (behaviour) | Race Gender/sex SES Age Education |
| Liu (2014) (ID:52950192) | Implementation of international code of marketing breast-milk substitutes in China | East As & Pac (China) | R- Advertising | National | Regulatory | Effectiveness | Quantitative (post CS) | Humans (behaviour, policy characts) Non-hum (food envt) | (none) |
| Long (2010) (ID:52952193) | Evaluating the impact of a connecticut program to reduce availability of unhealthy competitive food in schools | N Am (USA) | O- Settings | State | Volunt (public/non-profit) | Effectiveness | Quantitative (pre-post FU/TS) | Humans (food envt) | Age Education SES |
| Long (2013) (ID:52950813) | Impact of Connecticut legislation incentivizing elimination of unhealthy competitive foods on National School Lunch Program participation | N Am (USA) | O- Settings | State | Volunt (public/non-profit) | Effectiveness | Quantitative (post CS) | Humans (behaviour, food envt, policy characts) | SES Education |
| Lopez-Olmedo (2018) (ID:52947664) | The socioeconomic disparities in intakes and purchases of less-healthy foods and beverages have changed over time in urban Mexico | L Am & Car (Mexico) | U- Economic | National | Regulatory | Effectiveness | Quantitative (pre-post FU/TS) | Humans (behaviour) | Place SES |
| Lowery (2020) (ID:52946085) | Reformulation of packaged foods and beverages in the colombian food supply | L Am & Car (Colombia) | I- Reformulat | National | Volunt (private) | Effectiveness | Quantitative (post-post CS) | Non-hum (food envt) | (none) |
| Lwin (2020) (ID:52966815) | A macro-level assessment of introducing children food advertising restrictions on children's unhealthy food cognitions and behaviors | East As & Pac (Singapore) | R- Advertising | National | Regulatory | Effectiveness | Quantitative (pre-post FU/TS) | Humans (behaviour) Non-hum (food envt) | Gender/sex Age |
| Machado (2019) (ID:52947435) | Nutritional composition of brazilian food products marketed to children | L Am & Car (Brazil) | R- Advertising | National | Volunt (private) | Effectiveness | Quantitative (post CS) | Non-hum (food envt) | Age |
| Mantilla (2018) (ID:52958986) | Cost-Effectiveness of Product Reformulation in Response to the Health Star Rating Food Labelling System in Australia. | East As & Pac (Australia) | N- Labelling | International | Volunt (public/non-profit) | Cost-effectiveness | Quantitative (M/S) | N/A | N/A |
| Mayhew (2016) (ID:52949174) | Nutrition labelling, marketing techniques, nutrition claims and health claims on chip and biscuit packages from sixteen countries | N Am (Canada) L Am & Car (Argentina, Brazil, Chile, Colombia) East As & Pac (China, Malaysia) M East & N Afr (United Arab Emirates) -South Asia (India) Sub-Sah Afr (South Africa) Euro (Poland, Sweden, Turkey) | N- Labelling | National | Regulatory Volunt (public/non-profit) | Effectiveness | Quantitative (post CS) | Non-hum (food envt) | (none) |
| Mendoza-Velazquez (2019) (ID:52958583) | Special excise tax on food and beverages and its impact on inflation in Mexico in terms of dynamics, persistence, and change of regime | L Am & Car (Mexico) | U- Economic | National | Regulatory | Effectiveness | Quantitative (pre-post FU/TS) | Non-hum (food envt) | (none) |
| Merlo (2018) (ID:52947755) | State-Level Guidance and District-Level Policies and Practices for Food Marketing in US School Districts | N Am (USA) | O- Settings | State | Regulatory | Effectiveness | Quantitative (post CS) | Humans (policy characts) | Place Race |
| Meza-Hernandez (2020) (ID:52946425) | Nutritional quality of food and beverages offered in supermarkets of lima according to the peruvian law of healthy eating | L Am & Car (Peru) | N- Labelling | National | Regulatory | Effectiveness | Quantitative (post CS) | Non-hum (food envt) | (none) |
| Millett (2012) (ID:52951187) | Impacts of a national strategy to reduce population salt intake in England: Serial cross sectional study | Euro (UK) | I- Reformulat | National | Volunt (public/non-profit) | Effectiveness | Quantitative (pre-post CS) | Humans (behaviour) | Race Gender/sex Age Occupation |
| Monge-Rojas (2017) (ID:52948469) | Progress towards elimination of trans-fatty acids in foods commonly consumed in four Latin American cities | L Am & Car (Argentina, Brazil, Costa Rica, Mexico) | N- Labelling I- Reformulat | National International | Regulatory Volunt (public/non-profit) | Effectiveness | Quantitative (post-post CS) | Non-hum (food envt) | (none) |
| Montero-Campos (2015) (ID:52960321) | [Sodium in breads and snacks of high consumption in Costa Rica. Basal content and verification of nutrition labeling]. | L Am & Car (Costa Rica) | N- Labelling | International | Volunt (public/non-profit) | Effectiveness | Quantitative (post CS) | Non-hum (food envt) | (none) |
| Moorman (2012) (ID:52971543) | Unintended Nutrition Consequences: Firm Responses to the Nutrition Labeling and Education Act | N Am (USA) | N- Labelling | National | Regulatory | Effectiveness | Quantitative (pre-post CS) | Non-hum (other) | (none) |
| Moran (2017) (ID:52959565) | Trends in Nutrient Content of Children's Menu Items in U.S. Chain Restaurants. | N Am (USA) | N- Labelling | National | Volunt (private) | Effectiveness | Quantitative (post-post CS) | Non-hum (food envt) | (none) |
| Myszkowska-Ryciak (2018) (ID:52947955) | Nutrition-related practices in kindergartens in the context of changes to legal regulations on foodstuffs used in canteen menus for children | Euro (Poland) | O- Settings | National | Regulatory | Effectiveness | Quantitative (post-post FU/TS) | Humans (food envt) | Education |
| Nakamura (2018) (ID:52947487) | Evaluating the 2014 sugar-sweetened beverage tax in Chile: An observational study in urban areas | L Am & Car (Chile) | U- Economic | National | Regulatory | Effectiveness | Quantitative (pre-post FU/TS) | Humans (behaviour, food envt) | SES |
| Namba (2013) (ID:52946421) | Exploratory analysis of fast-food chain restaurant menus before and after implementation of local calorie-labeling policies, 2005-2011 | N Am (USA) | N- Labelling | State | Regulatory | Effectiveness | Quantitative (pre-post CS) | Humans (food envt) | Age |
| Neyens (2017) (ID:52948308) | Empty pledges: a content analysis of Belgian and Dutch child-targeting food websites | Euro (Belgium, Netherlands) | R- Advertising | International | Volunt (private) | Effectiveness | Quantitative (post CS) | Humans (food envt) | (none) |
| Ng (2014) (ID:52950043) | The healthy weight commitment foundation pledge: Calories purchased by U.S. households with children, 2000-2012 | N Am (USA) | I- Reformulat | National | Voluntary (PPP) | Effectiveness | Quantitative (pre-post FU/TS) | Humans (behaviour) | (none) |
| Ng (2014) (ID:52950044) | The healthy weight commitment foundation pledge: Calories sold from U.S. consumer packaged goods, 2007-2012 | N Am (USA) | I- Reformulat | National | Voluntary (PPP) | Effectiveness | Quantitative (pre-post CS) | Humans (behaviour) | Place Race Occupation Gender/sex SES Age |
| Ng (2018) (ID:52947890) | Did high sugar-sweetened beverage purchasers respond differently to the excise tax on sugar-sweetened beverages in Mexico? | L Am & Car (Mexico) | U- Economic | National | Regulatory | Effectiveness | Quantitative (pre-post FU/TS) | Humans (behaviour) | SES Education |
| Ni (2011) (ID:52961883) | Sodium content of processed foods in the United Kingdom: analysis of 44,000 foods purchased by 21,000 households. | Euro (UK) | I- Reformulat | National | Volunt (public/non-profit) | Effectiveness | Quantitative (post CS) | Humans (food envt) | (none) |
| Ni (2017) (ID:52948187) | Effects of a voluntary front-of-pack nutrition labelling system on packaged food reformulation: The health star rating system in New Zealand | East As & Pac (N Zealand) | N- Labelling | International | Volunt (public/non-profit) | Effectiveness | Quantitative (pre-post CS) | Non-hum (food envt) | (none) |
| Nicholas (2013) (ID:52950983) | The impact of the food-based and nutrient-based standards on lunchtime food and drink provision and consumption in secondary schools in England | Euro (UK) | O- Settings | State | Regulatory | Effectiveness | Quantitative (post CS) | Humans (food envt) | SES |
| Nicholson (2014) (ID:52950344) | State farm-to-school laws influence the availability of fruits and vegetables in school lunches at US public elementary schools | N Am (USA) | O- Settings | State | Regulatory Volunt (public/non-profit) | Effectiveness | Quantitative (post CS) | Humans (food envt) | (none) |
| Niederdeppe (2013) (ID:52950847) | News coverage of sugar-sweetened beverage taxes: pro- and antitax arguments in public discourse | N Am (USA) | U- Economic | State | Regulatory | News coverage | Quantitative (post CS) | N/A | N/A |
| Nilson (2017) (ID:52948098) | The impact of voluntary targets on the sodium content of processed foods in Brazil, 2011-2013 | L Am & Car (Brazil) | I- Reformulat | National | Volunt (public/non-profit) | Effectiveness | Quantitative (pre-post CS) | Non-hum (food envt) | (none) |
| Nilson (2017) (ID:52948195) | Sodium reduction in processed foods in Brazil: Analysis of food categories and voluntary targets from 2011 to 2017 | L Am & Car (Brazil) | I- Reformulat | National | Volunt (public/non-profit) | Effectiveness | Quantitative (pre-post CS) | Non-hum (food envt) | (none) |
| No (2014) (ID:52950024) | Food references and marketing in popular magazines for children and adolescents in New Zealand: A content analysis | East As & Pac (N Zealand) | R- Advertising | National | Volunt (private) | Effectiveness | Quantitative (post CS) | Non-hum (food envt) | Age |
| Oh (2016) (ID:52949084) | Correlates of Reported Use and Perceived Helpfulness of Calorie Information in Restaurants Among U.S. Adults | N Am (USA) | N- Labelling | National | Regulatory | Effectiveness | Quantitative (post CS) | Humans (behaviour) | Race Gender/sex Education Age Social capital |
| Okeyo (2020) (ID:52944033) | The food and nutrition environment at secondary schools in the Eastern Cape, South Africa as reported by learners | Sub-Sah Afr (South Africa) | O- Settings | National | Volunt (public/non-profit) | Effectiveness | Quantitative (post CS) | Humans (behaviour, food envt) | Place Race Gender/sex Education Age |
| Olsson (2016) (ID:52949006) | School meals do not have a given place in Swedish school's quality management | Euro (Sweden) | O- Settings | National | Regulatory | Effectiveness | Quantitative (post CS) | Humans (policy characts) | (none) |
| Olstad (2020) (ID:52966231) | Baseline results from the Eat, Play, Live trial: A randomized controlled trial within a natural experiment examining the role of nutrition policy and capacity building in improving food environments in recreation and sport facilities | N Am (Canada) | O- Settings | State | Volunt (public/non-profit) | Effectiveness | Quantitative (post CS) | Non-hum (food envt) | (none) |
| Otite (2013) (ID:52951158) | Trends in trans fatty acids reformulations of us supermarket and brand-name foods between 2007 and 2011 | N Am (USA) | I- Reformulat | National | Regulatory | Effectiveness | Quantitative (post-post CS) | Non-hum (food envt) | (none) |
| Ovrebo (2020) (ID:52956801) | The effects of an abrupt increase in taxes on candy and soda in Norway: an observational study of retail sales. | Euro (Norway) | U- Economic | National | Regulatory | Effectiveness | Quantitative (pre-post CS) | Humans (behaviour) | (none) |
| Ovrum (2014) (ID:52950291) | Evaluating free school fruit: results from a natural experiment in Norway with representative data | Euro (Norway) | O- Settings | National | Volunt (public/non-profit) | Effectiveness | Quantitative (post CS) | Humans (behaviour) | Age |
| Palakshappa (2016) (ID:52948882) | Association between state school nutrition laws and subsequent child obesity | N Am (USA) | O- Settings | State | Regulatory | Effectiveness | Quantitative (post CS) | Humans (health) | Education |
| Park (2020) (ID:52956605) | Progress on sodium reduction in South Korea. | East As & Pac (Rep Korea) | I- Reformulat | National | Volunt (public/non-profit) | Effectiveness | Quantitative (pre-post CS) | Humans (behaviour) | Gender/sex Age |
| Patterson (2015) (ID:52949778) | Improvements in school meal quality in Sweden after the introduction of new legislation-a 2-year follow-up | Euro (Sweden) | O- Settings | National | Regulatory | Effectiveness | Quantitative (pre-post FU/TS) | Humans (food envt) | (none) |
| Pearce (2011) (ID:52951653) | Short communicationKey differences between school lunches and packed lunches in primary schools in England in 2009 | Euro (UK) | O- Settings | State | Regulatory | Effectiveness | Quantitative (post CS) | Humans (behaviour) | (none) |
| Pearce (2013) (ID:52950790) | Portion weights of food served in English schools: have they changed following the introduction of nutrient-based standards? | Euro (UK) | O- Settings | State | Regulatory | Effectiveness | Quantitative (post-post CS) | Non-hum (food envt) | Education |
| Peart (2012) (ID:52951402) | Does competitive food and beverage legislation hurt meal participation or revenues in high schools? | N Am (USA) | O- Settings | State | Regulatory | Effectiveness | Quantitative (pre-post FU/TS) | Humans (behaviour, food envt) Non-hum (food envt) | (none) |
| Pedraza (2018) (ID:52947592) | Mexican households' purchases of foods and beverages vary by store-type, taxation status, and SES | L Am & Car (Mexico) | U- Economic | National | Regulatory | Effectiveness | Quantitative (pre-post FU/TS) | Humans (behaviour) | SES |
| Pedraza (2019) (ID:52946374) | The caloric and sugar content of beverages purchased at different store-types changed after the sugary drinks taxation in Mexico | L Am & Car (Mexico) | U- Economic | National | Regulatory | Effectiveness | Quantitative (pre-post FU/TS) | Humans (behaviour) Non-hum (food envt) | (none) |
| Pedraza (2020) (ID:52946497) | Mexican households' food shopping patterns in 2015: analysis following (none)ssential food and sugary beverage taxes | L Am & Car (Mexico) | U- Economic | National | Regulatory | Effectiveness | Quantitative (post CS) | Humans (behaviour) | SES |
| Pell (2019) (ID:52947199) | Support for, and perceived effectiveness of, the UK soft drinks industry levy among UK adults: Cross-sectional analysis of the International Food Policy Study | Euro (UK) | U- Economic | National | Regulatory | Public consultation | Quantitative (post CS) | N/A | N/A |
| Pell (2020) (ID:52956633) | Anticipatory changes in British household purchases of soft drinks associated with the announcement of the Soft Drinks Industry Levy: A controlled interrupted time series analysis. | Euro (UK) | U- Economic | National | Regulatory | Effectiveness | Quantitative (pre-post FU/TS) | Humans (behaviour) | (none) |
| Pereda (2020) (ID:52946580) | Price impact of taxes on sugary drinks in Brazil | L Am & Car (Brazil) | U- Economic | National | Regulatory | Effectiveness | Quantitative (pre-post CS) | Humans (behaviour) | (none) |
| Pereira Machado (2016) (ID:52935365) | Serving sizes and energy values on the nutrition labels of regular and diet/light processed and ultra-processed dairy products sold in Brazil | L Am & Car (Brazil) | N- Labelling | National | Regulatory | Effectiveness | Quantitative (post CS) | Non-hum (food envt) | (none) |
| Perez-Ferrer (2018) (ID:52947737) | Compliance with nutrition standards in Mexican schools and their effectiveness: a repeated cross-sectional study | L Am & Car (Mexico) | O- Settings | National | Regulatory | Effectiveness | Quantitative (post-post CS) | Humans (behaviour) Non-hum (food envt) | Place |
| Peters (2017) (ID:52948208) | The sodium content of processed foods in South Africa during the introduction of mandatory sodium limits | Sub-Sah Afr (South Africa) | I- Reformulat | National | Regulatory | Effectiveness | Quantitative (post CS) | Non-hum (food envt) | (none) |
| Phillips (2010) (ID:52952115) | Changes in school environments with implementation of arkansas act 1220 of 2003 | N Am (USA) | O- Settings | State | Regulatory | Effectiveness | Quantitative (pre-post CS) | Humans (food envt) | Education |
| Phulkerd (2020) (ID:52946083) | Changes in population-level consumption of taxed and non-taxed sugar-sweetened beverages (Ssb) after implementation of ssb excise tax in thailand: A prospective cohort study | East As & Pac (Thailand) | U- Economic | National | Regulatory | Effectiveness | Quantitative (post-post FU/TS) | Humans (behaviour) | Gender/sex Place Occupation Education SES Age |
| Pigat (2018) (ID:52947866) | A probabilistic intake model to estimate the impact of reformulation by the food industry among Irish consumers | Euro (Ireland) | I- Reformulat | National | Volunt (private) | Effectiveness | Quantitative (M/S) | Humans (behaviour) Non-hum (food envt) | Age |
| Pinto (2020) (ID:52946799) | Food and beverage advertising to children and adolescents on television: A baseline study | N Am (Canada) | R- Advertising | National | Volunt (private) | Effectiveness | Quantitative (post CS) | Non-hum (food envt) | Age |
| Pitts (2018) (ID:52967813) | One-Year Follow-Up Examination of the Impact of the North Carolina Healthy Food Small Retailer Program on Healthy Food Availability, Purchases, and Consumption | N Am (USA) | S- Retail & catering | State | Volunt (public/non-profit) | Effectiveness | Quantitative (pre-post CS) | Humans (behaviour, health) Non-hum (food envt) | SES |
| Polacsek (2012) (ID:52961544) | Examining compliance with a statewide law banning junk food and beverage marketing in Maine schools. | N Am (USA) | R- Advertising | State | Regulatory | Effectiveness  Implementation | Quantitative (post CS) | Humans (food envt, policy characts) Non-hum (food envt) | (none) |
| Potvin Kent (2011) (ID:52951911) | Self-regulation by industry of food marketing is having little impact during children's preferred television | N Am (Canada) | R- Advertising | National | Volunt (private) | Effectiveness | Quantitative (post CS) | Non-hum (food envt) | Age |
| Potvin Kent (2011) (ID:52961904) | Food marketing on children's television in two different policy environments. | N Am (Canada) | R- Advertising | National State | Regulatory Volunt (private) | Effectiveness | Quantitative (post CS) | Non-hum (food envt) | Race Age |
| Potvin Kent (2012) (ID:52951309) | A nutritional comparison of foods and beverages marketed to children in two advertising policy environments | N Am (Canada) | R- Advertising | National State | Regulatory Volunt (private) | Effectiveness | Quantitative (post CS) | Non-hum (food envt) | Race |
| Potvin Kent (2013) (ID:52950607) | Internet marketing directed at children on food and restaurant websites in two policy environments | N Am (Canada) | R- Advertising | National State | Regulatory Volunt (private) | Effectiveness | Quantitative (post CS) | Non-hum (food envt) | Race |
| Potvin Kent (2014) (ID:52950127) | The influence of the Children's Food and Beverage Advertising Initiative: Change in children's exposure to food advertising on television in Canada between 2006-2009 | N Am (Canada) | R- Advertising | National | Volunt (private) | Effectiveness | Quantitative (pre-post CS) | Humans (food envt) | (none) |
| Potvin Kent (2014) (ID:52950222) | Changes in the volume, power and nutritional quality of foods marketed to children on television in Canada | N Am (Canada) | R- Advertising | National | Volunt (private) | Effectiveness | Quantitative (pre-post CS) | Non-hum (food envt) | Age |
| Potvin Kent (2018) (ID:52947594) | The effectiveness of the food and beverage industry's self-established uniform nutrition criteria at improving the healthfulness of food advertising viewed by Canadian children on television | N Am (Canada) | R- Advertising | National | Volunt (private) | Effectiveness | Quantitative (pre-post CS) | Non-hum (food envt) | (none) |
| Potvin Kent (2018) (ID:52947811) | The effectiveness of self-regulation in limiting the advertising of unhealthy foods and beverages on children's preferred websites in Canada | N Am (Canada) | R- Advertising | National | Volunt (private) | Effectiveness | Quantitative (post CS) | Non-hum (food envt) | (none) |
| Potvin Kent (2020) (ID:52966064) | The physical activity and nutrition-related corporate social responsibility initiatives of food and beverage companies in Canada and implications for public health | N Am (Canada) | R- Advertising | National | Volunt (private) | Effectiveness | Quantitative (post CS) | Non-hum (policy characts) | (none) |
| Powell (2010) (ID:68341293) | Trends in Exposure to Television Food Advertisements Among Children and Adolescents in the United States | N Am (USA) | R- Advertising | National | Volunt (private) | Effectiveness | Quantitative (pre-post CS) | Humans (behaviour) | Race Age |
| Powell (2011) (ID:65923922) | Trends in the Nutritional Content of Television Food Advertisements Seen by Children in the United States | N Am (USA) | R- Advertising | National | Volunt (private) | Effectiveness | Quantitative (pre-post CS) | Non-hum (food envt) | Age |
| Powell (2013) (ID:68341292) | Nutritional Content of Food and Beverage Products in Television Advertisements Seen on Children's Programming | N Am (USA) | R- Advertising | National | Volunt (private) | Effectiveness | Quantitative (post CS) | Humans (behaviour) Non-hum (food envt) | Age |
| Prado (2020) (ID:52946784) | Compliance of infant formula promotion on websites of Brazilian manufacturers and drugstores | L Am & Car (Brazil) | R- Advertising | National | Regulatory | Effectiveness | Quantitative (post CS) | Non-hum (food envt) | (none) |
| Pravst (2017) (ID:52948185) | Changes in average sodium content of prepacked foods in Slovenia during 2011-2015 | Euro (Slovenia) | I- Reformulat | National | Volunt (public/non-profit) | Effectiveness | Quantitative (post-post CS) | Humans (behaviour) Non-hum (food envt) | (none) |
| Prowse (2018) (ID:52947707) | Food marketing in recreational sport settings in Canada: A cross-sectional audit in different policy environments using the Food and beverage Marketing Assessment Tool for Settings (FoodMATS) | N Am (Canada) | O- Settings | State | Volunt (public/non-profit) | Effectiveness | Quantitative (post CS) | Non-hum (food envt) | Age |
| Prowse (2019) (ID:52946935) | Exploring Nutrition Labelling of Food and Beverages in Vending Machines in Canadian Recreational Sport Settings | N Am (Canada) | O- Settings | State | Volunt (public/non-profit) | Effectiveness | Quantitative (post CS) | Non-hum (food envt) | (none) |
| Pulker (2018) (ID:52947504) | Alignment of supermarket own brand foods' front-of-pack nutrition labelling with measures of nutritional quality: An australian perspective | East As & Pac (Australia) | N- Labelling | International | Volunt (public/non-profit) | Effectiveness | Quantitative (post CS) | Non-hum (food envt) | (none) |
| Pulker (2018) (ID:52947944) | Ultra-processed family foods in Australia: nutrition claims, health claims and marketing techniques | East As & Pac (Australia) | N- Labelling | International | Volunt (public/non-profit) | Effectiveness | Quantitative (post CS) | Non-hum (food envt) | Age |
| Quitral (2019) (ID:52967413) | Comparison of sugar and non- caloric sweetener content in beverages before and after implementing Chilean law 20.606 | L Am & Car (Chile) | N- Labelling O- Settings R- Advertising | National | Regulatory | Effectiveness | Quantitative (pre-post CS) | Non-hum (food envt) | (none) |
| Rajbhandari-Thapa (2017) (ID:52948532) | Effect of the Strong4Life School Nutrition Program on Cafeterias and on Manager and Staff Member Knowledge and Practice, Georgia, 2015 | N Am (USA) | O- Settings | National State | Regulatory Voluntary (PPP) | Effectiveness  Implementation | Quantitative (pre-post FU/TS) | Humans (food envt) | (none) |
| Ramos (2015) (ID:52960447) | Influence of Spanish TV commercials on child obesity. | Euro (Spain) | R- Advertising | National | Volunt (private) | Effectiveness | Quantitative (post CS) | Non-hum (food envt) | Age |
| Ratnayake (2014) (ID:52950161) | Mandatory trans fat labeling regulations and nationwide product reformulations to reduce trans fatty acid content in foods contributed to lowered concentrations of trans fat in Canadian women's breast milk samples collected in 2009-2011 | N Am (Canada) | N- Labelling I- Reformulat | National | Mixed | Effectiveness | Quantitative (pre-post CS) | Humans (health) | (none) |
| Reilly (2019) (ID:52967016) | Secondary school implementation of a healthy eating policy | East As & Pac (Australia) | O- Settings | State | Regulatory | Effectiveness | Quantitative (post CS) | Non-hum (food envt) | Place Religion Education |
| Restrepo (2016) (ID:52969836) | Denmark's Policy on Artificial Trans Fat and Cardiovascular Disease | Euro (Denmark) | I- Reformulat | National | Regulatory | Effectiveness | Quantitative (pre-post CS) | Humans (health) | (none) |
| Restrepo (2020) (ID:52935403) | The effects of soda taxes on adolescent sugar intake and blood sugar | N Am (USA) | U- Economic | State | Regulatory | Effectiveness | Quantitative (post-post FU/TS) | Humans (behaviour, health) | (none) |
| Restrepo (2020) (ID:52957702) | Intake of trans-fats among US youth declined from 1999-2000 to 2009-2010. | N Am (USA) | I- Reformulat | National | Regulatory | Effectiveness | Quantitative (pre-post CS) | Humans (health) | (none) |
| Reyes (2020) (ID:52957959) | Changes in the amount of nutrient of packaged foods and beverages after the initial implementation of the Chilean Law of Food Labelling and Advertising: A (none)xperimental prospective study. | L Am & Car (Chile) | N- Labelling | National | Regulatory | Effectiveness | Quantitative (pre-post CS) | Non-hum (food envt) | (none) |
| Ribeiro (2013) (ID:52971195) | Processed foods aimed at children and adolescents: Sodium content, adequacy according to the dietary reference intakes and label compliance | L Am & Car (Brazil) | N- Labelling | National | Regulatory | Effectiveness | Quantitative (post CS) | Non-hum (food envt) | (none) |
| Ricardo (2019) (ID:52958253) | Trans Fat Labeling Information on Brazilian Packaged Foods. | L Am & Car (Brazil) | N- Labelling | National | Regulatory | Effectiveness | Quantitative (Post CS) | Non-hum (food envt) | (none) |
| Rida (2018) (ID:52967873) | Assessment of Nutrition Knowledge of Childcare Providers Regarding the Implementation of the 2017 CACFP Meal Pattern Update | N Am (USA) | O- Settings | National | Volunt (public/non-profit) | Implementation | Quantitative (post CS) | N/A | N/A |
| Riis (2012) (ID:52964979) | State school policies and youth obesity. | N Am (USA) | O- Settings R- Advertising | State | Regulatory | Effectiveness | Quantitative (pre-post CS) | Humans (health) | Education |
| Rippin (2017) (ID:52968563) | An exploration of socio-economic and food characteristics of high trans fatty acid consumers in the Dutch and UK national surveys after voluntary product reformulation | Euro (Netherlands, UK) | I- Reformulat | National | Volunt (private) Volunt (public/non-profit) | Effectiveness | Quantitative (post CS) | Humans (behaviour) | Race Occupation Gender/sex Education SES Age Place |
| Ritchie (2015) (ID:52949874) | Policy improves what beverages are served to young children in child care | N Am (USA) | O- Settings | National | Volunt (public/non-profit) | Implementation Effectiveness | Quantitative (pre-post CS) | Humans (food envt) | (none) |
| Roberto (2012) (ID:52951346) | Choosing front-of-package food labelling nutritional criteria: how smart were 'Smart Choices'? | N Am (USA) | N- Labelling | National | Volunt (private) | Effectiveness | Quantitative (post CS) | Non-hum (food envt) | (none) |
| Roberts (2012) (ID:52951500) | Compliance with children's television food advertising regulations in Australia | East As & Pac (Australia) | R- Advertising | National | Regulatory Volunt (private) | Effectiveness | Quantitative (post CS) | Non-hum (food envt) | (none) |
| Roberts (2014) (ID:52970864) | Children's exposure to food advertising: An analysis of the effectiveness of self-regulatory codes in Australia | East As & Pac (Australia) | R- Advertising | National | Volunt (private) | Effectiveness | Quantitative (post CS) | Non-hum (food envt) | Age |
| Robinson (2019) (ID:52946306) | Point of choice kilocalorie labelling in the UK eating out of home sector: a descriptive study of major chains | Euro (UK) | N- Labelling | State | Voluntary (PPP) | Effectiveness | Quantitative (post CS) | Humans (food envt) Non-hum (food envt) | (none) |
| Royo-Bordonada (2016) (ID:52948999) | The extent and nature of food advertising to children on Spanish television in 2012 using an international food-based coding system and the UK nutrient profiling model | Euro (Spain) | R- Advertising | National | Volunt (private) | Effectiveness | Quantitative (post CS) | Non-hum (food envt) | (none) |
| Salgado (2019) (ID:52944456) | Understanding Heterogeneity in Price Changes and Firm Responses to a National Unhealthy Food Tax in Mexico | L Am & Car (Mexico) | U- Economic | National | Regulatory | Effectiveness | Quantitative (pre-post FU/TS) | Humans (food envt) | (none) |
| Samuels (2010) (ID:52952138) | Implementation of California State School Competitive Food and Beverage Standards | N Am (USA) | O- Settings | State | Regulatory | Effectiveness | Quantitative (pre-post CS) | Non-hum (food envt) | Education |
| Sanchez-Romero (2016) (ID:52948995) | Projected Impact of Mexico's Sugar-Sweetened Beverage Tax Policy on Diabetes and Cardiovascular Disease: A Modeling Study | L Am & Car (Mexico) | U- Economic | National | Regulatory | Cost-effectiveness | Quantitative (M/S) | N/A | N/A |
| Sanchez-Romero (2020) (ID:52946710) | Association between tax on sugar sweetened beverages and soft drink consumption in adults in Mexico: Open cohort longitudinal analysis of Health Workers Cohort Study | L Am & Car (Mexico) | U- Economic | National | Regulatory | Effectiveness | Quantitative (pre-post FU/TS) | Humans (behaviour) | Education SES |
| Sanchez-Vaznaugh (2010) (ID:52962110) | 'Competitive' food and beverage policies: are they influencing childhood overweight trends?. | N Am (USA) | O- Settings | State | Regulatory | Effectiveness | Quantitative (pre-post CS) | Humans (health) | Gender/sex Education |
| Sandoval (2019) (ID:52946448) | The effect of 'Traffic-Light' nutritional labelling in carbonated soft drink purchases in Ecuador | L Am & Car (Ecuador) | N- Labelling | National | Regulatory | Effectiveness | Quantitative (pre-post FU/TS) | Humans (behaviour, food envt) | SES |
| Sanjeevi (2020) (ID:52946126) | Stronger State School Nutrition Laws Are Associated With Healthier Eating Behaviors and Optimal Weight Status in US Adolescents | N Am (USA) | O- Settings | State | Regulatory | Effectiveness | Quantitative (post-post FU/TS) | Humans (behaviour, health) | Race Gender/sex Education SES |
| Sarda (2020) (ID:63632509) | Appropriation of the Front-of-Pack Nutrition Label Nutri-Score across the French Population: Evolution of Awareness, Support, and Purchasing Behaviors between 2018 and 2019 | Euro (France) | N- Labelling | National | Volunt (public/non-profit) | Effectiveness | Quantitative (post-post CS) | Humans (behaviour) | Occupation Gender/sex Education SES Age |
| Scarborough (2020) (ID:52946480) | Impact of the announcement and implementation of the UK soft drinks industry levy on sugar content, price, product size and number of available soft drinks in the UK, 2015-19: A controlled interrupted time series analysis | Euro (UK) | U- Economic | National | Regulatory | Effectiveness | Quantitative (pre-post FU/TS) | Non-hum (food envt) | (none) |
| Scarpelli (2020) (ID:52946465) | Changes in nutrient declaration after the food labeling and advertising law in Chile: A longitudinal approach | L Am & Car (Chile) | N- Labelling | National | Regulatory | Effectiveness | Quantitative (pre-post CS) | Non-hum (food envt) | (none) |
| Schmacker (2020) (ID:52946822) | Do prices and purchases respond similarly to soft drink tax increases and cuts? | Euro (Denmark) | U- Economic | National | Regulatory | Effectiveness | Quantitative (pre-post FU/TS) | Humans (behaviour, food envt) | (none) |
| Schwartz (2015) (ID:52970000) | Comparing Current Practice to Recommendations for the Child and Adult Care Food Program | N Am (USA) | O- Settings | National | Volunt (public/non-profit) | Effectiveness | Quantitative (post CS) | Non-hum (food envt) | Gender/sex |
| Scourboutakos (2019) (ID:52947270) | Assessing the Early Impact of Menu-Labeling on Calories in Chain Restaurants in Ontario, Canada | N Am (Canada) | N- Labelling | State | Regulatory | Effectiveness | Quantitative (pre-post CS) | Non-hum (food envt) | (none) |
| Shahid (2020) (ID:52946418) | Uptake of australia's health star rating system 2014-2019 | East As & Pac (Australia) | N- Labelling | International | Volunt (public/non-profit) | Effectiveness | Quantitative (pre-post CS) | Non-hum (food envt) | (none) |
| Shankar (2012) (ID:72748917) | An evaluation of the UK Food Standards Agency's salt campaign | Euro (UK) | I- Reformulat | National | Volunt (public/non-profit) | Effectiveness | Quantitative (pre-post CS) | Humans (behaviour) | SES Gender/sex Education |
| Silva (2015) (ID:52935420) | An Evaluation of the Effect of Child-Directed Television Food Advertising Regulation in the United Kingdom | Euro (UK) | R- Advertising | National | Regulatory Volunt (private) | Effectiveness | Quantitative (pre-post FU/TS) | Humans (behaviour) | Age |
| Silveira (2013) (ID:52950968) | Reporting of trans-fat on labels of Brazilian food products | L Am & Car (Brazil) | N- Labelling | National | Regulatory | Effectiveness | Quantitative (post CS) | Non-hum (food envt) | (none) |
| Smed (2016) (ID:52948823) | The effects of the Danish saturated fat tax on food and nutrient intake and modelled health outcomes: An econometric and comparative risk assessment evaluation | Euro (Denmark) | U- Economic | National | Regulatory | Effectiveness | Quantitative (M/S) | Humans (behaviour, health) Non-hum (food envt) | Gender/sex Age |
| Smed (2019) (ID:52946283) | The effects of voluntary front-of-pack nutrition labels on volume shares of products: the case of the Dutch Choices | Euro (Denmark) | N- Labelling | National | Volunt (public/non-profit) | Effectiveness | Quantitative (pre-post CS) | Humans (behaviour) | (none) |
| Smith (2013) (ID:52950869) | Infant food marketing strategies undermine effective regulation of breast-milk substitutes: trends in print advertising in Australia, 1950-2010 | East As & Pac (Australia) | R- Advertising | International National | Volunt (private) Volunt (public/non-profit) | Effectiveness | Quantitative (pre-post CS) | Non-hum (food envt) | (none) |
| Smith (2015) (ID:63155262) | Gains Made By Walmart's Healthier Food Initiative Mirror Preexisting Trends | N Am (USA) | N- Labelling U- Economic S- Retail & catering | National | Volunt (private) | Effectiveness | Quantitative (pre-post CS) | Humans (behaviour) | (none) |
| Smith (2016) (ID:63155263) | Walmart and Other Food Retail Chains: Trends and Disparities in the Nutritional Profile of Packaged Food Purchases | N Am (USA) | N- Labelling S- Retail & catering U- Economic | National | Volunt (private) | Effectiveness | Quantitative (pre-post CS) | Humans (behaviour) | Race SES |
| Smithers (2019) (ID:52935526) | Discretionary food advertising on television in 2017: a descriptive study | East As & Pac (Australia) | R- Advertising | National | Volunt (private) | Effectiveness | Quantitative (post CS) | Non-hum (food envt) | Age |
| Sparks (2018) (ID:52947566) | Sodium levels of processed meat in australia: Supermarket survey data from 2010 to 2017 | East As & Pac (Australia) | I- Reformulat | National | Voluntary (PPP) | Effectiveness | Quantitative (pre-post CS) | Non-hum (food envt) | (none) |
| Speers (2011) (ID:52951857) | Child and adolescent exposure to food and beverage brand appearances during prime-time television programming | N Am (USA) | R- Advertising | National | Volunt (private) | Effectiveness | Quantitative (post CS) | Non-hum (food envt) | Age |
| Spiteri (2018) (ID:52947953) | Nutritional quality of new food products released into the Australian retail food market in 2015 - is the food industry part of the solution? | East As & Pac (Australia) | I- Reformulat | National | Volunt (private) | Effectiveness | Quantitative (post CS) | Non-hum (food envt) | Age |
| Stacey (2019) (ID:52947045) | Sugar-based beverage taxes and beverage prices: Evidence from South Africa's Health Promotion Levy | Sub-Sah Afr (South Africa) | U- Economic | National | Regulatory | Effectiveness | Quantitative (pre-post FU/TS) | Non-hum (food envt) | (none) |
| Stender (2014) (ID:52970961) | Tracing artificial trans fat in popular foods in Europe: a market basket investigation | Euro (Austria, Denmark, Iceland, Switzerland) | I- Reformulat | National | Regulatory | Effectiveness | Quantitative (Post CS) | Non-hum (food envt) | Race |
| Stevens (2013) (ID:52950984) | School lunches v. packed lunches: a comparison of secondary schools in England following the introduction of compulsory school food standards | Euro (UK) | O- Settings | State | Regulatory | Effectiveness | Quantitative (post CS) | Humans (food envt) Non-hum (food envt) | (none) |
| Stoltze (2019) (ID:52947134) | Prevalence of child-directed marketing on breakfast cereal packages before and after chile's food marketing law: A pre-and post-quantitative content analysis | L Am & Car (Chile) | N- Labelling | National | Regulatory | Effectiveness | Quantitative (pre-post CS) | Non-hum (food envt) | Age |
| Storey (2015) (ID:52949706) | Changes in mean intake of fatty acids and intake of saturated and trans fats from potatoes: NHANES 2005-2006, 2007-2008, and 2009-2010 | N Am (USA) | I- Reformulat | National | Regulatory | Effectiveness | Quantitative (pre-post CS) | Humans (behaviour) | Age |
| Sturm (2010) (ID:52935428) | Soda Taxes, Soft Drink Consumption, And Children's Body Mass Index | N Am (USA) | U- Economic | State | Regulatory | Effectiveness | Quantitative (post-post CS) | Humans (behaviour, health) | Race Gender/sex Education SES |
| Taber (2011) (ID:52965098) | State policies targeting junk food in schools: Racial/ethnic differences in the effect of policy change on soda consumption. | N Am (USA) | O- Settings | State | Regulatory Volunt (public/non-profit) | Effectiveness | Quantitative (post CS) | Humans (behaviour, health) | Race |
| Taber (2012) (ID:52951213) | Weight status among adolescents in states that govern competitive food nutrition content | N Am (USA) | O- Settings | State | Regulatory | Effectiveness | Quantitative (post-post CS) | Humans (health) | (none) |
| Taber (2012) (ID:52951241) | Banning all sugar-sweetened beverages in middle schools: Reduction of in-school access and purchasing but not overall consumption | N Am (USA) | O- Settings | State | Regulatory | Effectiveness | Quantitative (post-post CS) | Humans (behaviour, food envt) | (none) |
| Taber (2012) (ID:52951380) | Differences in nutrient intake associated with state laws regarding fat, sugar, and caloric content of competitive foods | N Am (USA) | O- Settings | State | Regulatory | Effectiveness | Quantitative (post CS) | Humans (behaviour) | Race |
| Taber (2013) (ID:52950657) | Association between state laws governing school meal nutrition content and student weight status: Implications for new USDA school meal standards | N Am (USA) | O- Settings | State | Regulatory | Effectiveness | Quantitative (post CS) | Humans (behaviour, health) | Place Race SES |
| Taber (2014) (ID:52950131) | How state taxes and policies targeting soda consumption modify the association between school vending machines and student dietary behaviors: A cross-sectional analysis | N Am (USA) | O- Settings U- Economic | State | Regulatory | Effectiveness | Quantitative (post CS) | Humans (behaviour) | (none) |
| Taber (2015) (ID:52949582) | The association between state bans on soda only and adolescent substitution with other sugar-sweetened beverages: A cross-sectional study | N Am (USA) | O- Settings | State | Regulatory | Effectiveness | Quantitative (Post CS) | Humans (behaviour, food envt) | Race Gender/sex |
| Taber (2015) (ID:52949678) | Socioeconomic Differences in the Association Between Competitive Food Laws and the School Food Environment | N Am (USA) | O- Settings | State | Regulatory | Effectiveness | Quantitative (post-post CS) | Humans (food envt) | SES |
| Taher (2020) (ID:52946596) | Cross-sectional associations between lunch-type consumed on a school day and British adolescents' overall diet quality | Euro (UK) | O- Settings | State | Regulatory | Effectiveness | Quantitative (pre-post CS) | Humans (behaviour) | Age Gender/sex |
| Taillie (2017) (ID:52948095) | Do high vs. low purchasers respond differently to a (none)ssential energy-dense food tax? Two-year evaluation of Mexico's 8% (none)ssential food tax | L Am & Car (Mexico) | U- Economic | National | Regulatory | Effectiveness | Quantitative (pre-post CS) | Humans (behaviour) | (none) |
| Taillie (2020) (ID:52946481) | An evaluation of Chile's law of food labeling and advertising on sugar-sweetened beverage purchases from 2015 to 2017: A before-and-after study | L Am & Car (Chile) | N- Labelling O- Settings R- Advertising | National | Regulatory | Effectiveness | Quantitative (pre-post CS) | Humans (behaviour) | Education |
| Tatlow-Golden, (2015) (ID:59693157) | Creating good feelings about unhealthy food: children's televised 'advertised diet' on the island of Ireland, in a climate of regulation | Euro (Ireland, UK) | R- Advertising | National | Regulatory | Effectiveness | Quantitative (post CS) | Non-hum (food envt) | (none) |
| Teng (2020) (ID:52946493) | Sweetened beverage taxes and changes in beverage price, imports and manufacturing: Interrupted time series analysis in a middle-income country | East As & Pac (Tonga) | U- Economic | National | Regulatory | Effectiveness | Quantitative (pre-post CS) | Non-hum (food envt) | (none) |
| Terry-Mcelrath (2012) (ID:52951284) | Factors affecting sugar-sweetened beverage availability in competitive venues of us secondary schools | N Am (USA) | O- Settings | National | Regulatory | Effectiveness  Implementation | Quantitative (pre-post CS) | Humans (food envt) | Education |
| Terry-Mcelrath (2015) (ID:52949447) | Regular soda policies, school availability, and high school student consumption | N Am (USA) | O- Settings | State | Regulatory | Effectiveness | Quantitative (post CS) | Humans (behaviour, food envt) | Race Gender/sex Education |
| Theodore (2017) (ID:52948287) | Pitfalls of the self-regulation of advertisements directed at children on Mexican television | L Am & Car (Mexico) | R- Advertising | National | Volunt (private) | Effectiveness | Quantitative (post CS) | Non-hum (food envt) | Age |
| Theodore (2018) (ID:52958940) | Lessons learned and insights from the implementation of a food and physical activity policy to prevent obesity in Mexican schools: An analysis of nationally representative survey results. | L Am & Car (Mexico) | O- Settings | National | Regulatory | Implementation | Quantitative (post CS) | N/A | N/A |
| Todd (2021) (ID:52935692) | Food away from home and caloric intake: The role of restaurant menu labeling laws | N Am (USA) | N- Labelling | State | Regulatory | Effectiveness | Quantitative (pre-post CS) | Humans (behaviour) | Age Race Education |
| Torres-Alvarez (2020) (ID:52946556) | Body weight impact of the sugar-sweetened beverages tax in Mexican children: A modeling study | L Am & Car (Mexico) | U- Economic | National | Regulatory | Effectiveness | Quantitative (M/S) | Humans (behaviour, health) | Age |
| Trandafilovic (2018) (ID:52936888) | Researching consumer habits regarding food label reading | Euro (Serbia) | N- Labelling | International | Regulatory | Effectiveness | Quantitative (post CS) | Humans (behaviour) | Gender/sex Education Age |
| Trevena (2014) (ID:52950276) | The Australian Food and Health Dialogue - the implications of the sodium recommendation for pasta sauces | East As & Pac (Australia) | I- Reformulat | National | Voluntary (PPP) | Effectiveness | Quantitative (pre-pre CS) | Non-hum (food envt) | (none) |
| Trevena (2014) (ID:68341298) | An Evaluation of the Effects of the Australian Food and Health Dialogue Targets on the Sodium Content of Bread, Breakfast Cereals and Processed Meats | East As & Pac (Australia) | I- Reformulat | National | Voluntary (PPP) | Effectiveness | Quantitative (post-post CS) | Non-hum (food envt) | (none) |
| Turner (2012) (ID:52951202) | Healthier Fundraising in U. S. Elementary Schools: Associations between Policies at the State, District, and School Levels | N Am (USA) | O- Settings | State | Regulatory | Effectiveness | Quantitative (post CS) | Humans (food envt) | Race Education |
| Turner (2013) (ID:52971089) | Classroom Parties in US Elementary Schools: The Potential for Policies to Reduce Student Exposure to Sugary Foods and Beverages | N Am (USA) | O- Settings | State | Regulatory | Effectiveness | Quantitative (post CS) | Humans (food envt) | Race Education |
| Turner (2014) (ID:52964576) | Perceived reactions of elementary school students to changes in school lunches after implementation of the United States Department of Agriculture's new meals standards: Minimal backlash, but rural and socioeconomic disparities exist. | N Am (USA) | O- Settings | National | Regulatory | Effectiveness | Quantitative (post CS) | Humans (behaviour) | Place SES |
| Turner (2020) (ID:52946944) | Association of State Laws Regarding Snacks in US Schools with Students' Consumption of Solid Fats and Added Sugars | N Am (USA) | O- Settings | National State | Regulatory | Effectiveness | Quantitative (post CS) | Humans (behaviour) | Race Gender/sex Education Age |
| Urquiaga (2014) (ID:52970762) | Assessment of the reliability of food labeling in Chile | L Am & Car (Chile) | N- Labelling | National | Regulatory | Effectiveness | Quantitative (post CS) | Non-hum (food envt) | (none) |
| USDA (2013) (ID:59693513) | Evaluation of the Fresh Fruit and Vegetable Program (FFVP): Final Evaluation Report | N Am (USA) | O- Settings | National | Volunt (public/non-profit) | Effectiveness  Implementation | Quantitative (post CS) | Humans (behaviour) | Race Gender/sex SES Age |
| Ustjanauskas (2014) (ID:52950090) | Food and beverage advertising on children's web sites | N Am (USA) | R- Advertising | National | Volunt (private) | Effectiveness | Quantitative (post CS) | Non-hum (food envt) | (none) |
| Vaala (2020) (ID:52946826) | Child-Oriented Marketing on Cereal Packaging: Associations With Sugar Content and Manufacturer Pledge | N Am (USA) | R- Advertising | National | Volunt (private) | Effectiveness | Quantitative (post CS) | Non-hum (food envt) | (none) |
| Vall (2020) (ID:52944427) | Impact of SSB Taxes on Sales | Euro (Spain) | U- Economic | State | Regulatory | Effectiveness | Quantitative (pre-post FU/TS) | Humans (behaviour, food envt) | Place |
| Van Camp (2012) (ID:52945253) | Stop or Go? How Is the UK Food Industry Responding to Front-of-Pack Nutrition Labels? | Euro (UK) | N- Labelling | National | Volunt (private) | Effectiveness | Quantitative (post CS) | Non-hum (food envt) | (none) |
| Van Camp (2012) (ID:52951428) | Changes in fat contents of US snack foods in response to mandatory trans fat labelling | N Am (USA) | N- Labelling | National | Regulatory | Effectiveness  News coverage | Quantitative (pre-post CS) | Non-hum (food envt) | (none) |
| Vanderlee (2019) (ID:52947427) | Evaluation of a voluntary nutritional information program versus calorie labelling on menus in Canadian restaurants: A quasi-experimental study design | N Am (Canada) | N- Labelling | State | Regulatory Volunt (public/non-profit) | Effectiveness | Quantitative (pre-post CS) | Humans (behaviour) | (none) |
| Vandevijvere (2020) (ID:52956728) | Uptake of Nutri-Score during the first year of implementation in Belgium. | Euro (Belgium) | N- Labelling | National | Volunt (public/non-profit) | Effectiveness | Quantitative (post CS) | Non-hum (food envt) | (none) |
| Vergeer (2019) (ID:52967786) | The effectiveness of voluntary policies and commitments in restricting unhealthy food marketing to Canadian children on food company websites | N Am (Canada) | R- Advertising | National | Volunt (private) | Effectiveness | Quantitative (post CS) | Non-hum (food envt) | Age |
| Vermote (2020) (ID:69573699) | Nutritional Content, Labelling and Marketing of Breakfast Cereals on the Belgian Market and Their Reformulation in Anticipation of the Implementation of the Nutri-Score Front-Of-Pack Labelling System | Euro (Belgium) | N- Labelling | National | Volunt (public/non-profit) | Effectiveness | Quantitative (pre-post CS) | Non-hum (food envt) | (none) |
| Vidal (2015) (ID:52970229) | School menus in Santa Catarina: Evaluation with respect to the National School Food Program regulations | L Am & Car (Brazil) | O- Settings | National | Regulatory | Effectiveness | Quantitative (post CS) | Non-hum (food envt) | (none) |
| Vieux (2013) (ID:52950796) | Dietary standards for school catering in france: Serving moderate quantities to improve dietary quality without increasing the food-related cost of meals | Euro (France) | O- Settings | National | Volunt (public/non-profit) | Implementation | Quantitative (pre-post CS) | N/A | N/A |
| Vilaro (2017) (ID:52948306) | Weekday and weekend food advertising varies on children's television in the USA but persuasive techniques and unhealthy items still dominate | N Am (USA) | R- Advertising | National | Volunt (private) | Effectiveness | Quantitative (post-post CS) | Non-hum (food envt) | Age |
| Vinje (2017) (ID:52948485) | Media audit reveals inappropriate promotion of products under the scope of the International Code of Marketing of Breast-milk Substitutes in SouthEast Asia | East As & Pac (Cambodia, Indonesia, Myanmar, Thailand, Vietnam) | R- Advertising | National International | Regulatory Volunt (public/non-profit) | Effectiveness  News coverage | Quantitative (post CS) | Non-hum (food envt, policy characts) | Age |
| Vyth (2010) (ID:59693897) | Actual use of a front-of-pack nutrition logo in the supermarket: consumers' motives in food choice | Euro (Netherlands) | N- Labelling | National | Volunt (public/non-profit) | Effectiveness | Quantitative (post CS) | Humans (behaviour) Non-hum (food envt) | Gender/sex Education |
| Wang (2011) (ID:52951630) | The changes of nutrition labeling of packaged food in hangzhou in china during 2008~2010 | East As & Pac (China) | N- Labelling | National | Volunt (public/non-profit) | Effectiveness | Quantitative (post-post CS) | Non-hum (food envt) | (none) |
| Wang (2016) (ID:52935435) | The impact of mandatory trans fat labeling on product mix and consumer choice: A longitudinal analysis of the U.S. Market for margarine and spreads | N Am (USA) | N- Labelling | National | Regulatory | Effectiveness | Quantitative (pre-post FU/TS) | Humans (behaviour, food envt) | Education SES Age |
| Watson (2014) (ID:52970671) | Determining the 'healthiness' of foods marketed to children on television using the Food Standards Australia New Zealand nutrient profiling criteria | East As & Pac (Australia) | R- Advertising | National | Volunt (private) | Effectiveness | Quantitative (post CS) | Non-hum (food envt) | Age |
| Watson (2017) (ID:52968576) | Advertising to children initiatives have not reduced unhealthy food advertising on Australian television | East As & Pac (Australia) | R- Advertising | National | Volunt (private) | Effectiveness | Quantitative (post CS) | Non-hum (food envt) | Age |
| Wellard (2011) (ID:52954297) | Fast facts: The availability and accessibility of nutrition information in fast food chains. | East As & Pac (Australia) | N- Labelling | National | Volunt (private) | Effectiveness | Quantitative (post CS) | Non-hum (food envt) | Place SES Age |
| Wellard (2015) (ID:52949771) | The availability and accessibility of nutrition information in fast food outlets in five states post-menu labelling legislation in New South Wales | East As & Pac (Australia) | N- Labelling | State | Regulatory | Effectiveness | Quantitative (pre-post CS) | Non-hum (food envt) | Place Age |
| Wellard (2016) (ID:52969376) | Investigating nutrient profiling and Health Star Ratings on core dairy products in Australia | East As & Pac (Australia) | N- Labelling | International | Volunt (public/non-profit) | Effectiveness | Quantitative (post CS) | Non-hum (food envt) | (none) |
| Wellard-Cole (2018) (ID:52947853) | Monitoring the changes to the nutrient composition of fast foods following the introduction of menu labelling in New South Wales, Australia: an observational study | East As & Pac (Australia) | N- Labelling | State | Regulatory | Effectiveness | Quantitative (pre-post CS) | Non-hum (food envt) | (none) |
| Wellard-Cole (2019) (ID:52946162) | Nutrient composition of Australian fast-food and fast-casual children's meals available in 2016 and changes in fast-food meals between 2010 and 2016 | East As & Pac (Australia) | S- Retail & catering | National State | Regulatory Volunt (private) | Effectiveness | Quantitative (post-post CS) | Non-hum (food envt) | Age |
| Wescott (2012) (ID:52936903) | Industry Self-Regulation to Improve Student Health: Quantifying Changes in Beverage Shipments to Schools | N Am (USA) | O- Settings | National | Volunt (private) | Effectiveness | Quantitative (pre-post CS) | Non-hum (food envt) | Education |
| Whalen (2019) (ID:52947299) | Children's exposure to food advertising: the impact of statutory restrictions | Euro (UK) | R- Advertising | National | Regulatory | Effectiveness | Quantitative (post-post CS) | Non-hum (food envt) | Age |
| Whatley (2011) (ID:52954086) | Impact of Maine's statewide nutrition policy on high school food environments. | N Am (USA) | O- Settings | State | Regulatory | Effectiveness | Quantitative (pre-post CS) | Humans (food envt) | (none) |
| White (2016) (ID:52949024) | A voluntary nutrition labeling program in restaurants: Consumer awareness, use of nutrition information, and food selection | N Am (Canada) | N- Labelling | National | Volunt (public/non-profit) | Effectiveness | Quantitative (post CS) | Humans (behaviour) | (none) |
| WHO (2016) (ID:59692979) | Assessment of the impact of a public health product tax | Euro (Hungary) | U- Economic | National | Regulatory | Effectiveness | Quantitative (post CS) | Humans (behaviour, health) Non-hum (other) | Education SES |
| Wiecha (2018) (ID:52946543) | Survey of Afterschool Programs Suggests Most Offer Fruit and Vegetables Daily | N Am (USA) | O- Settings | National | Volunt (public/non-profit) | Effectiveness | Quantitative (post CS) | Humans (food envt) | (none) |
| Wu (2014) (ID:52950300) | Changes in the energy and sodium content of main entrees in US chain restaurants from 2010 to 2011 | N Am (USA) | N- Labelling | National | Regulatory | Effectiveness | Quantitative (pre-post CS) | Non-hum (food envt) | Age |
| Yon (2015) (ID:52969988) | New School Meal Regulations and Consumption of Flavored Milk in Ten US Elementary Schools, 2010 and 2013 | N Am (USA) | O- Settings | National | Regulatory | Effectiveness | Quantitative (pre-post CS) | Humans (behaviour) | Gender/sex Age |
| Zaltz (2018) (ID:52947808) | Barriers and Facilitators to Compliance with a State Healthy Eating Policy in Early Care and Education Centers | N Am (USA) | O- Settings | National State | Volunt (public/non-profit) | Implementation | Quantitative (post CS) | N/A | N/A |
| Zhang (2016) (ID:52960021) | Usage and Understanding of Serving Size Information on Food Labels in the United States. | N Am (USA) | N- Labelling | National | Regulatory | Effectiveness | Quantitative (post-post CS) | Humans (behaviour) | Race Gender/sex Education Age |
| Zheng (2019) (ID:52935552) | Reducing Obesity by Taxing Soft Drinks: Tax Salience and Firms' Strategic Responses | N Am (USA) | U- Economic | State | Regulatory | Effectiveness | Quantitative (post-post CS) | Humans (behaviour) | (none) |
| Zupanic (2019) (ID:52947039) | Free sugar content in pre-packaged products: Does voluntary product reformulation work in practice? | Euro (Slovenia) | I- Reformulat | National | Volunt (private) | Effectiveness | Quantitative (post-post CS) | Non-hum (food envt) | Age |

Legend: **Policy categories according the WCRF NOURISHING:** N- Labelling ; O- Specific settings (schools, nurseries, healthcare, leisure/sports centres); U- Economic interventions (taxes and price reductions); R; I- Reformulation by manufacturers; S- Retail & catering services; **World Bank** **regions & countries**: East As & Pac = East Asia & Pacific; Euro = Europe; L Am & Car = Latin American & Caribbean; N Am = North America; M East & N Afr = Middle East & North Africa; Sub-Sah Afr = Sub-Saharan Africa; N Zealand = New Zealand; Rep Korea = Republic of Korea; **Other categories:** CS = Cross-sectional; Food envt = Food environment characteristics; FU/TS = Follow-up studies & time series; M/S = Modelling & Scenarios; Non-hum = Non-humans; Pol characts = Policy characteristics & status; SES = Socieconomic Status; Volunt = Voluntary

# Supplementary Table 5: Full-texts excluded for selected reasons (n=174)

This Table lists the full-texts excluded for the following reasons:

- Wrong policy level (eligibility code no 6)
- Wrong evaluation level (code no 7)
- Policy inventories (code no 9)
- Views of the general public except public consultations (code no 10)
- Insufficient focus on governance (for multiple policies, code 11)
- Overviews of reviews (code no 12)
- Wrong policy level, evaluation or general public views in the UK (code 13 so they could be retrieved separately) from those above
- Full-texts that we could not obtain

| **First author (year) (internal record ID)** | **Reason for exclusion** |
| --- | --- |
| Aaron (2017) (ID:52948221) | 9: Policy inventory |
| Abildso (2019) (ID:52947431) | 6: Policy at the local level, not in the UK |
| Abiola (2019) (ID:52946449) | 9: Policy inventory |
| Adams (2012) (ID:59693515) | 13: UK – local evaluation (northeast of England) |
| Akkaya (2017) (ID:52944594) | 7: Evaluation at the local level, not in the UK |
| Al-Bahlani (2014) (ID:52970758) | 9: Policy inventory |
| Allen (2020) (ID:52947019) | 9: Policy inventory |
| Amin (2015) (ID:52954304) | 7: Evaluation at the local level, not in the UK |
| Ayers (2020) (ID:52946319) | 9: Policy inventory |
| Bagwell (2014) (ID:52950129) | 13: UK – local policy & evaluation (London, England) |
| Bakhtiari (2020) (ID:52946635) | 9: Policy inventory |
| Barberio (2017) (ID:59594889) | 11: Evidence synthesis not considering governance |
| Beebeejaun (2019) (ID:52967036) | 7: Evaluation at the local level, not in the UK |
| Beets (2014) (ID:52970711) | 7: Evaluation at the local level, not in the UK |
| Bergallo (2018) (ID:52947722) | 9: Policy inventory |
| Bollinger (2011) (ID:52954891) | 6: Policy at the local level, not in the UK |
| Breda (2020) (ID:52965817) | 9: Policy inventory |
| Brock (2017) (ID:52939276) | 9: Policy inventory |
| Buse (2020) (ID:52935740) | 9: Policy inventory |
| Calancie (2015) (ID:52949711) | 7: Evaluation at the local level, not in the UK |
| Caputo (2020) (ID:52944393) | 10: Views of the general public, not in the UK |
| Cetthakrikul (2019) (ID:52947044) | 9: Policy inventory |
| Chan (2011) (ID:52965055) | 7: Evaluation at the local level, not in the UK |
| Chan (2018) (ID:52963943) | 11: Primary study of multiple policies of unclear governance approaches |
| Cole (2017) (ID:52948329) | 7: Evaluation at the local level, not in the UK |
| Correa (2019) (ID:52947158) | 7: Evaluation at the local level, not in the UK |
| Cortes (2017) (ID:52968987) | 10: Views of the general public, not in the UK |
| Crockett (2018) (ID:52947686) | 6: Policy at the local level, not in the UK |
| Cullerton (2020) (ID:52946498) | 10: Views of the general public, not in the UK |
| Dias (2018) (ID:52947954) | 7: Evaluation at the local level, not in the UK |
| Darfour-Oduro (2020) (ID:52963684) | 11: Primary study of multiple policies of unclear governance approaches |
| Donaldson (2015) (ID:52949658) | 9: Policy inventory |
| Duran (2020) (ID:52946672) | 10: Views of the general public, not in the UK |
| Duran-Aguero (2017) (ID:52969091) | 10: Views of the general public, not in the UK |
| Elbel (2011) (ID:52936524) | 6: Policy at the local level, not in the UK |
| Ensaff (2013) (ID:56993435) | 13: UK – local evaluation (two schools in Yorkshire, England) |
| Esdaile (2019) (ID:52947233) | 9: Policy inventory |
| Eyler (2012) (ID:52951410) | 9: Policy inventory |
| Fitzpatrick (2017) (ID:52948239) | 7: Evaluation at the local level, not in the UK |
| Fletcher (2014) (ID:52964628) | 13: UK – local evaluation (six schools in London and South-East England) |
| Ford (2020) (ID:52957776) | 13: UK – local evaluation (urban central Scotland) |
| Foster (2018) (ID:52958710) | 9: Policy inventory |
| Garcia (2017) (ID:52948542) | 6: Policy at the local level, not in the UK |
| Gillison (2020) (ID:52946643) | 10: Views of the general public, not in the UK |
| Gourdet (2014) (ID:52950367) | 9: Policy inventory |
| Greathouse (2014) (ID:52950262) | 9: Policy inventory |
| Green (2018) (ID:52947770) | 7: Evaluation at the local level, not in the UK |
| Gregori (2019) (ID:52946311) | 10: Views of the general public, not in the UK |
| Gruner (2018) (ID:52963939) | 7: Evaluation at the local level, not in the UK |
| Hajdú (2018) (ID:52935639) | 10: Views of the general public, not in the UK |
| Hanratty (2012) (ID:52951455) | 13: UK – local policy & evaluation (local authority in North-West England) |
| Hardy (2019) (ID:52946452) | 9: Policy inventory |
| Hawkes (2011) (ID:52951799) | 9: Policy inventory |
| Hawkes (2011) (ID:52951651) | 9: Policy inventory |
| Hersey (2010) (ID:52965274) | 9: Policy inventory |
| Huang (2018) (ID:52947559) | 9: Policy inventory |
| Ickovics (2019) (ID:52941215) | 7: Evaluation at the local level, not in the UK |
| James (2017) (ID:52948114) | 13: UK – local evaluation (two hospitals in the south-west of England) |
| Jensen (2015) (ID:52949506) | 9: Policy inventory |
| Jia (2017) (ID:52964059) | 6: Policy at the local level, not in the UK |
| Jongenelis (2017) (ID:52959354) | 10: Views of the general public, not in the UK |
| Jou (2014) (ID:52950351) | 6: Policy at the local level, not in the UK |
| Julia (2015) (ID:52949792) | 10: Views of the general public, not in the UK |
| Kasture (2019) (ID:52946284) | 9: Policy inventory |
| Keeble (2019) (ID:52947271) | 9: Policy inventory |
| Keeble (2019) (ID:52946795) | 13: UK – local policy (local authority level, England) |
| Keeble (2019) (ID:52946841) | 13: UK – local policy (local authority level, England) |
| Kehm (2015) (ID:52949751) | 9: Policy inventory |
| Kersting (2020) (ID:52956635) | 9: Policy inventory |
| Kim (2011) (ID:52951789) | 9: Policy inventory |
| Knox (2020) (ID:52944379) | 10: Views of the general public, not in the UK |
| Kubik (2011) (ID:52961884) | 7: Evaluation at the local level, not in the UK |
| Kubik (2015) (ID:52964381) | 6: Policy at the local level, not in the UK |
| Kuntz (2012) (ID:52964862) | 7: Evaluation at the local level, not in the UK |
| Lam (2018) (ID:52947792) | 13: UK – local evaluation (one city in Eastern England) |
| Lange (2019) (ID:52946333) | 6: Policy at the local level, not in the UK |
| Lankford (2013) (ID:52950930) | 9: Policy inventory |
| Larson (2011) (ID:52956279) | 9: Policy inventory |
| Lawlis (2017) (ID:52948421) | 7: Evaluation at the local level, not in the UK |
| Lee (2020) (ID:52946097) | 9: Policy inventory |
| Lee (2020) (ID:52946031) | 9: Policy inventory |
| Lhotakova (2015) (ID:52944780) | 10: Views of the general public, not in the UK |
| Limbu (2019) (ID:52935749) | 7: Evaluation at the local level, not in the UK |
| Lin (2016) (ID:52969570) | 10: Views of the general public, not in the UK |
| Lloyd-Williams (2014) (ID:52950256) | 9: Policy inventory |
| Mann (2017) (ID:52948473) | 7: Evaluation at the local level, not in the UK |
| Mann (2018) (ID:52959163) | 7: Evaluation at the local level, not in the UK |
| Maria (2017) (ID:52968972) | 7: Evaluation at the local level, not in the UK |
| Mariath (2020) (ID:52946471) | 9: Policy inventory |
| Marriott (2020) (ID:52946864) | 6: Policy at the local level, not in the UK |
| Martinez (2020) (ID:52946423) | 7: Evaluation at the local level, not in the UK |
| Masis (2017) (ID:52948463) | 7: Evaluation at the local level, not in the UK |
| Masse (2013) (ID:52950963) | 9: Policy inventory |
| Maubach (2010) (ID:52972281) | 10: Views of the general public, not in the UK |
| Mazariegos (2017) (ID:52942203) | 7: Evaluation at the local level, not in the UK |
| McGuffin (2013) (ID:52950714) | 9: Policy inventory |
| McLaren (2016) (ID:59603959) | 11: Evidence synthesis not considering governance |
| McLean (2014) (ID:52950280) | 10: Views of the general public, not in the UK |
| McSweeney (2018) (ID:52947569) | 13: UK – local evaluation (one hospital in the northeast of England) |
| Melendez-Illanes (2019) (ID:52967206) | 7: Evaluation at the local level, not in the UK |
| Mendez (2015) (ID:52969971) | 7: Evaluation at the local level, not in the UK |
| Micha (2018) (ID:60657841) | 11: Evidence synthesis not considering governance |
| Miller (2017) (ID:52944749) | 7: Evaluation at the local level, not in the UK |
| Moore (2010) (ID:52965260) | 13: UK – local evaluation (one local authority in Wales) |
| Moreira (2019) (ID:52935739) | 10: Views of the general public, not in the UK |
| Moretto (2014) (ID:52950181) | 7: Evaluation at the local level, not in the UK |
| Musicus (2020) (ID:52946172) | 9: Policy inventory |
| Nathan (2016) (ID:52941140) | 7: Evaluation at the local level, not in the UK |
| Ng (2018) (ID:52947789) | 9: Policy inventory |
| Ng (2020) (ID:52946746) | 9: Policy inventory |
| Nivedhakumari (2020) (ID:52946650) | 7: Evaluation at the local level, not in the UK |
| Nixon (2015) (ID:52970325) | 6: Policy at the local level, not in the UK |
| Ohene-Darko (2020) (ID:52943410) | 7: Evaluation at the local level, not in the UK |
| Olivares (2017) (ID:52959455) | 10: Views of the general public, not in the UK |
| Olstad (2011) (ID:52951746) | 7: Evaluation at the local level, not in the UK |
| Olstad (2016) (ID:59600886) | 11: Evidence synthesis not considering governance |
| Ortega-Avila (2018) (ID:52947898) | 10: Views of the general public, not in the UK |
| Patino (2020) (ID:52946813) | 9: Policy inventory |
| Pearce (2013) (ID:52950988) | 13: UK – local evaluation (4 local authorities in Essex, Leicester City, Manchester and Sheffield) |
| Penn-Newman (2018) (ID:52947468) | 9: Policy inventory |
| Phulkerd (2017) (ID:52948502) | 9: Policy inventory |
| Pomeranz (2017) (ID:52948090) | 9: Policy inventory |
| Pomeranz (2019) (ID:52944518) | 9: Policy inventory |
| Pomeranz (2020) (ID:52946292) | 9: Policy inventory |
| Pulos (2010) (ID:52935443) | 6: Policy at the local level, not in the UK |
| Reis (2011) (ID:52946628) | 9: Policy inventory |
| Rejman (2011) (ID:52954268) | 7: Evaluation at the local level, not in the UK |
| Robertson-James (2017) (ID:52959278) | 6: Policy at the local level, not in the UK |
| Rodriguez-Fernandez (2014) (ID:52970713) | 9: Policy inventory |
| Rosewarne (2020) (ID:52946582) | 9: Policy inventory |
| Sacks (2020) (ID:52946365) | 9: Policy inventory |
| Schneider (2012) (ID:52951210) | 9: Policy inventory |
| Shi (2018) (ID:52947870) | 7: Evaluation at the local level, not in the UK |
| Shroff (2012) (ID:52936644) | 9: Policy inventory |
| Sinclair (2014) (ID:59603951) | 6: Policy at the local level, not in the UK |
| Sisnowski (2015) (ID:52960448) | 9: Policy inventory |
| Sloan (2020) (ID:52957603) | 9: Policy inventory |
| Soares (2017) (ID:52963987) | 7: Evaluation at the local level, not in the UK |
| Soekarjo (2018) (ID:52947892) | 9: Policy inventory |
| Sofí­a (2020) (ID:56539181) | 9: Policy inventory |
| Solh (2010) (ID:52952442) | 6: Policy at the local level, not in the UK |
| Spence (2013) (ID:52950667) | 13: UK – local evaluation (12 schools in North East England) |
| Spence (2014) (ID:59693182) | 13: UK – local evaluation (six schools in Northumberland, England) |
| Stran (2013) (ID:52950840) | Could not be obtained |
| Swartz (2011) (ID:59603669) | 6: Policy at the local level, not in the UK |
| Swift 2018 (ID:52967983) | 13: UK – explores general public views other than public consultations) |
| Sze (2016) (ID:59387195) | 7: Evaluation at the local level, not in the UK |
| Taylor (2019) (ID:52935514) | 6: Policy at the local level, not in the UK |
| Thomas-Meyer (2017) (ID:52948129) | 13: UK – explores general public views other than public consultations) |
| Tuangratananon (2019) (ID:52958662) | 9: Policy inventory |
| Vallgarda (2018) (ID:52947534) | 9: Policy inventory |
| van Herpen (2014) (ID:52960936) | 6: Policy at the local level, not in the UK |
| Vandevijvere (2015) (ID:52936419) | 9: Policy inventory |
| Vandevijvere (2019) (ID:52947231) | 9: Policy inventory |
| Velasco (2019) (ID:52963660) | 7: Evaluation at the local level, not in the UK |
| Vercammen (2020) (ID:52946685) | 9: Policy inventory |
| Vergeer (2020) (ID:52956696) | 9: Policy inventory |
| Vézina-Im (2017) (ID:52956104) | 11: Evidence synthesis not considering governance |
| von Philipsborn (2018) (ID:52958910) | 9: Policy inventory |
| von Philipsborn (2018) (ID:52947791) | 9: Policy inventory |
| Watson (2017) (ID:52947840) | 9: Policy inventory |
| Webster (2011) (ID:59700515) | 9: Policy inventory |
| White (2020) (ID:52946381) | 7: Evaluation at the local level, not in the UK |
| Win (2020) (ID:52946331) | 9: Policy inventory |
| Wright (2019) (ID:52946787) | 6: Policy at the local level, not in the UK |
| Wu (2013) (ID:52954735) | 9: Policy inventory |
| Zaganjor (2018) (ID:52963858) | 9: Policy inventory |
| Zheng (2019) (ID:52935452) | 6: Policy at the local level, not in the UK |
| Zheng (2019) (ID:52967436) | 6: Policy at the local level, not in the UK |
| Zhou (2015) (ID:52949849). | Could not be obtained |
| Zorbas (2020) (ID:52946764) | 9: Policy inventory |
| Zurita-Corvalan (2018) (ID:52968067) | 7: Evaluation at the local level, not in the UK |

**Note:** Perez-Escamilla (2018) (ID:52947859) was included in the evidence map but not the policy it describes for the UK since it was implemented at the local level (Birmingham, England).

# Supplementary Table 6: Number of publications by World Bank region and country (n=482)

Note: a publication can cover more than one World Bank region and country (non-mutually exclusive category).

| **World Bank regions & countries** | **N publications** |
| --- | --- |
| **East Asia & Pacific** | **87** |
| Australia | 50 |
| Cambodia | 1 |
| China | 6 |
| Fiji | 5 |
| French Polynesia | 2 |
| Indonesia | 2 |
| South Korea | 9 |
| Malaysia | 4 |
| Myanmar | 1 |
| Nauru | 2 |
| New Zealand | 12 |
| Philippines | 2 |
| Samoa | 3 |
| Singapore | 2 |
| Thailand | 6 |
| Tonga | 1 |
| Vietnam | 2 |
| Belgium | 4 |
| Bulgaria | 1 |
| Croatia | 1 |
| Czech Republic | 1 |
| Denmark | 13 |
| Estonia | 1 |
| Finland | 4 |
| France | 14 |
| Germany | 5 |
| Greece | 1 |
| Hungary | 5 |
| Iceland | 2 |
| Ireland | 5 |
| Italy | 1 |
| Latvia | 2 |
| Lithuania | 1 |
| Netherlands | 6 |
| Norway | 3 |
| Poland | 4 |
| Portugal | 5 |
| Romania | 1 |
| Russia | 1 |
| Serbia | 2 |
| Slovakia | 1 |
| Slovenia | 6 |
| Spain | 14 |
| Sweden | 5 |
| Switzerland | 3 |
| Turkey | 2 |
| United Kingdom | 54 |
| **Latin America & Caribbean** | **93** |
| Argentina | 7 |
| Barbados | 2 |
| Brazil | 25 |
| Chile | 19 |
| Colombia | 7 |
| Costa Rica | 4 |
| Ecuador | 4 |
| Guatemala | 2 |
| Jamaica | 1 |
| Mexico | 40 |
| Peru | 4 |
| Puerto Rico | 1 |
| Uruguay | 1 |
| Venezuela | 1 |
| **Middle East & North Africa** | **6** |
| Israel | 1 |
| Saudi Arabia | 4 |
| United Arab Emirates | 2 |
| **North America** | **185** |
| Canada | 41 |
| United States | 146 |
| **South Asia** | **5** |
| India | 5 |
| **Sub-Saharan Africa** | **12** |
| Nigeria | 1 |
| South Africa | 11 |
| **Unclear / not reported** | **1** |

# Supplementary Table 7: Name of the policies included by country, policy level and N publications

As per information reported primarily in the studies. The international policies are listed in orange at the end. Please note that the number of publications by country reported in Supplementary Table 6 and 7 do not always match. This is because in Supplementary Table 7, some are listed under an international policy (e.g., Gulf Cooperation Council Pledge) rather than under the specific country (e.g., Saudia Arabia), depending on the focus of the evaluation.

| **Policy** | **Policy level** | | | **Governance approach** | | | | | **N publi-cations** |
| --- | --- | --- | --- | --- | --- | --- | --- | --- | --- |
|  | **Int’l** | **Nation-al** | **State** | **Mandatory** | **Voluntary (by public sector)** | **Voluntary (by private sector)** | **Voluntary**  **(PPP)** | **Mixed** |  |
| **Argentina** | | | | | | | | | |
| Back-of-pack labelling |  | x |  | x |  |  |  |  | 1 |
| Less Salt, More Life |  | x |  |  |  |  | x |  | 1 |
| Salt reduction |  | x |  | x |  |  |  |  | 2 |
| Trans fat limits |  | x |  | x |  |  |  |  | 3 |
| **Australia** | | | | | | | | | |
| Association of National Advertisers code |  | x |  |  |  | x |  |  | 1 |
| Australian Children’s Television Standards |  | x |  | x |  |  |  |  | 2 |
| Coca-Cola Soft Drink Reformulation Strategy |  | x |  |  |  | x |  |  | 1 |
| Daily Intake Guide (DIG) label |  | x |  |  |  | x |  |  | 1 |
| Food and Health Dialogue (including one study before the targets deadline) |  | x |  |  |  |  | x |  | 9 |
| Food policy in health facilities |  |  | x | x |  |  |  |  | 3 |
| Healthy Australia Commitment |  | x |  |  |  | x |  |  | 1 |
| Healthy Food Partnership |  | x |  |  |  |  | x |  | 2 |
| Marketing in Australia of Infant Formulas: Manufacturers and Importers Agreement (MAIF) |  | x |  |  |  | x |  |  | 2 |
| Menu kilojoule labelling |  |  | x | x |  |  |  |  | 5 |
| Quick Service Restaurant Industry’s Initiative (QSRII) |  | x |  |  |  | x |  |  | 8 |
| Australian Responsible Children's Marketing Initiative (RCMI) |  | x |  |  |  | x |  |  | 9 |
| Salt reduction |  |  | x |  | x |  |  |  | 1 |
| Salt reduction (Australian Division of World Action on Salt and Health; AWASH) |  | x |  |  | x |  |  |  | 1 |
| School Food policies |  |  | x | x |  |  |  |  | 2 |
| Trans fat pledge |  | x |  |  |  | X |  |  | 1 |
| **Austria** | | | | | | | | | |
| Code of Conduct for the Austrian Radio Broadcasting Organizers |  | x |  |  |  | x |  |  | 1 |
| Trans-fat limit |  | x |  | x |  |  |  |  | 2 |
| **Barbados** | | | | | | | | | |
| SSB tax |  | x |  | x |  |  |  |  | 2 |
| **Belgium** | | | | | | | | | |
| The Belgium Pledge |  | x |  |  |  | x |  |  | 1 |
| Nutri-Score label |  | x |  |  | x |  |  |  | 2 |
| **Brazil** | | | | | | | | | |
| Advertising restrictions (different regulations) |  | x |  | x |  |  |  |  | 3 |
| Marketing control of breastmilk substitutes |  | x |  | x |  |  |  |  | 3 |
| Marketing to children on packages |  | x |  |  |  | x |  |  | 1 |
| Health in Action |  | x |  |  |  |  | x |  | 1 |
| Back-of-pack labelling |  | x |  | x |  |  |  |  | 6 |
| Salt reduction |  | x |  |  | x |  |  |  | 2 |
| School food policy |  |  | x | x |  |  |  |  | 1 |
| School Food Programme (PNAE) |  | x |  | x |  |  |  |  | 2 |
| Trans-fat labelling |  | x |  | x |  |  |  |  | 3 |
| Trans-fat ban/limit (mandatory) + menu labelling (mandatory; proposition) + recommendations in general food-related national policies and for marketing (voluntary) |  | x |  |  |  |  |  | x | 1 |
| Trans fat labelling and ban on the hydrogenation of vegetable oil |  | x |  | x |  |  |  |  | 1 |
| SSB tax reduction |  | x |  | x |  |  |  |  | 1 |
| **Bulgaria** | | | | | | | | | |
| Framework for Responsible Commercial Communication of Food and Drinks |  | x |  | x |  |  |  |  | 1 |
| **Cambodia** | | | | | | | | | |
| Marketing control of breastmilk substitutes |  | x |  | x |  |  |  |  | 1 |
| **Canada** | | | | | | | | | |
| Advertising policy |  |  | x | x |  |  |  |  | 4 |
| Children's Food and Beverage Advertising Initiative (CAI) |  | x |  |  |  | x |  |  | 13 |
| Food policy in public recreational facilities |  |  | x |  | x |  |  |  | 5 |
| Health Check menu labelling |  | x |  |  | x |  |  |  | 1 |
| Healthy Eating Strategy |  | x |  |  |  |  |  | x | 1 |
| Back-of-pack labelling |  | x |  | x |  |  |  |  | 2 |
| Menu labelling (some mandatory, some voluntary/PPP) |  |  | x |  |  |  |  | x | 4 |
| Salt reduction public information/education campaign |  | x |  |  | x |  |  |  | 1 |
| School food policy (some mandatory, some voluntary by public or voluntary sector) |  |  | x |  |  |  |  | x | 6 |
| Trans-fat labelling (mandatory) + trans-fat limits (voluntary) |  | x |  |  |  |  |  | x | 3 |
| Trans-fats limits mentioned alone |  | x |  |  | x |  |  |  | 1 |
| Trans-fat limit in food services establishments |  |  | x | x |  |  |  |  | 1 |
| **Chile** | | | | | | | | | |
| Back-of-pack labelling |  | x |  | x |  |  |  |  | 1 |
| Back-of-pack labelling |  | x |  |  | x |  |  |  | 1 |
| Chilean Code of Advertising Ethics |  | x |  |  |  | x |  |  | 1 |
| Law of Food Labeling and Advertising (including one study before implementation) |  | x |  | x |  |  |  |  | 10 |
| SSB tax |  | x |  | x |  |  |  |  | 5 |
| Trans-fat limits (mandatory) + labelling (mandatory) + recommendations in a general food-related national policy (voluntary) + advertising code (voluntary) |  | x |  |  |  |  |  | x | 1 |
| **China** | | | | | | | | | |
| Back-of-pack labelling (2007-2013) |  | x |  |  | x |  |  |  | 2 |
| Back-of-pack labelling (2013) |  | x |  | x |  |  |  |  | 1 |
| Chinese Regulations of the WHO Code for marketing of breastmilk substitutes |  | x |  | x |  |  |  |  | 1 |
| Mondelez Hope Kitchen |  | x |  |  |  |  | x |  | 1 |
| **Colombia** | | | | | | | | | |
| Back-of-pack labelling |  | x |  | x |  |  |  |  | 1 |
| Columbian Code of Advertising Self-Regulation |  | x |  |  |  | x |  |  | 2 |
| Front-of-pack warning labels |  | x |  | x |  |  |  |  | 1 |
| “Responsible self-regulation” agreement |  | x |  |  |  | x |  |  | 1 |
| School food policy (Law No 1355) |  | x |  | x |  |  |  |  | 1 |
| Trans-fat labelling |  | x |  |  | x |  |  |  | 1 |
| **Costa Rica** | | | | | | | | | |
| Trans-fat recommendation in general food-related national policies |  | x |  |  | x |  |  |  | 1 |
| **Czech Republic** | | | | | | | | | |
| Czech Advertising Standards Council Code of Advertising Practice |  | x |  |  |  | x |  |  | 1 |
| **Denmark** | | | | | | | | | |
| Advertising restrictions |  | x |  |  |  |  |  | x | 1 |
| Soft drink tax |  | x |  | x |  |  |  |  | 1 |
| Tax on saturated fat |  | x |  | x |  |  |  |  | 7 |
| Trans-fat limit/ban on foods |  | x |  | x |  |  |  |  | 3 |
| **Ecuador** | | | | | | | | | |
| Advertising restrictions - National Assembly of Ecuador Law on Communications |  | x |  | x |  |  |  |  | 1 |
| Traffic Light |  | x |  | x |  |  |  |  | 2 |
| Trans-fat labelling (mandatory) + recommendation in a general food-related national policy (voluntary) |  | x |  |  |  |  |  | x | 1 |
| **Fiji** | | | | | | | | | |
| Advertizing and Promotion of Unhealthy Foods and Non-Alcoholic Beverages to Children Regulation |  | x |  | x |  |  |  |  | 2 |
| Marketing control of breastmilk substitutes |  | x |  | x |  |  |  |  | 2 |
| Salt reduction policies |  | x |  |  | x |  |  |  | 1 |
| SSB tax |  | x |  | x |  |  |  |  | 2 |
| **Finland** | | | | | | | | | |
| Advertising restrictions |  | x |  |  |  | x |  |  | 1 |
| School food standards & universal free school meals |  | x |  | x |  |  |  |  | 2 |
| Tax on sweets, ice cream and soft drinks |  | x |  | x |  |  |  |  | 1 |
| **France** | | | | | | | | | |
| Advertising restrictions |  | x |  | x |  |  |  |  | 1 |
| Advertising restrictions |  | x |  |  |  |  |  | x | 1 |
| FoP labelling |  | x |  |  |  |  |  | x | 1 |
| Nutri-Score |  | x |  |  | x |  |  |  | 1 |
| School Food Standards (2001-2011) |  | x |  |  | x |  |  |  | 3 |
| Soda tax |  | x |  | x |  |  |  |  | 5 |
| Trans-fat reduction |  | x |  |  | x |  |  |  | 1 |
| Vending machine ban |  | x |  | x |  |  |  |  | 1 |
| **French Polynesia** | | | | | | | | | |
| SSB tax |  | x |  | x |  |  |  |  | 2 |
| **Germany** | | | | | | | | | |
| German Standards Advertising Council (Deutscher Werberat) Code of Conduct on Commercial Communications for Foods and Beverages |  | x |  |  |  | x |  |  | 1 |
| Trans fat guidelines |  | x |  |  |  |  | x |  | 1 |
| **Guatemala** | | | | | | | | | |
| Nutrition Facts Label |  | x |  |  | x |  |  |  | 1 |
| **Hungary** | | | | | | | | | |
| Hungarian Advertising Code of Ethics, Article 18 |  | x |  |  |  | x |  |  | 1 |
| Public health product tax |  | x |  | x |  |  |  |  | 3 |
| School food standards & subsidized/free meals |  | x |  | x |  |  |  |  | 1 |
| **Iceland** | | | | | | | | | |
| School food standards & universal free school meals |  | x |  | x |  |  |  |  | 1 |
| Trans-fat ban |  | x |  | x |  |  |  |  | 1 |
| **India** | | | | | | | | | |
| Back-of-pack labelling |  | x |  | x |  |  |  |  | 1 |
| Back-of-pack labelling |  | x |  |  | x |  |  |  | 1 |
| Advertising pledge |  | x |  |  |  | x |  |  | 1 |
| Marketing control of breast milk substitutes |  | x |  | x |  |  |  |  | 1 |
| Shubh Aarambh |  | x |  |  |  |  | x |  | 1 |
| **Indonesia** | | | | | | | | | |
| Advertising restrictions |  | x |  |  |  |  |  | x | 1 |
| Marketing control of breastmilk substitutes |  | x |  | x |  |  |  |  | 1 |
| **Ireland** |  |  |  |  |  |  |  |  |  |
| Advertising restrictions for children |  | x |  |  |  |  |  | x | 1 |
| Advertising restrictions for children |  | x |  | x |  |  |  |  | 1 |
| Food and Drink Industry Ireland |  | x |  |  |  | x |  |  | 1 |
| Calorie Menu Labelling scheme |  | x |  |  | x |  |  |  | 1 |
| SSB tax |  | x |  | x |  |  |  |  | 1 |
| **Israel** | | | | | | | | | |
| Choices Logo |  | x |  |  | x |  |  |  | 1 |
| **Jamaica** | | | | | | | | | |
| Trans-fat labelling |  | x |  |  | x |  |  |  | 1 |
| **Latvia** | | | | | | | | | |
| School food standards |  | x |  | x |  |  |  |  | 1 |
| **Malaysia** | | | | | | | | | |
| Advertising restrictions |  | x |  |  |  | x |  |  | 1 |
| Advertising restrictions |  | x |  |  |  |  |  | x | 1 |
| Back-of-pack labelling |  | x |  | x |  |  |  |  | 1 |
| Back-of-pack labelling (mandatory for some food, voluntary for others) |  | x |  |  |  |  |  | x | 1 |
| **Mexico** | | | | | | | | | |
| Advertising restrictions to children |  | x |  |  |  | x |  |  | 1 |
| Advertising restrictions |  | x |  |  |  |  |  | x | 1 |
| Food (mandatory) and physical activity policy |  | x |  | x |  |  |  |  | 1 |
| Nutritional Guidelines for Food Sold in Schools |  | x |  | x |  |  |  |  | 1 |
| Partnership for Child Well-being |  | x |  |  |  |  | x |  | 1 |
| Regulation of Food and Beverage Advertisements to Children through Media |  | x |  | x |  |  |  |  | 1 |
| School Food Standards |  | x |  | x |  |  |  |  | 1 |
| SSB Tax |  | x |  | x |  |  |  |  | 19 |
| Tax on nonessential/ processed foods |  | x |  | x |  |  |  |  | 3 |
| 11 % SSB excise tax and 8 % energy-dense food ad valorem tax combined |  | x |  | x |  |  |  |  | 9 |
| Trans-fat limits (mandatory) + labelling (voluntary) + recommendation in a general food-related national policy (voluntary) |  | x |  |  |  |  |  | x | 1 |
| Especificaciones generales de etiquetado para alimentos y bebidas no alcohólicas preenvasados |  | x |  |  | x |  |  |  | 1 |
| **Myanmar** | | | | | | | | | |
| Marketing control of breastmilk substitutes |  | x |  | x |  |  |  |  | 1 |
| **Nauru** | | | | | | | | | |
| SSB tax |  | x |  | x |  |  |  |  | 2 |
| **Netherlands (the)** | | | | | | | | | |
| Dutch Advertising Code, Advertising Code for Food Products |  | x |  |  |  | x |  |  | 1 |
| Healthy Choice logo |  | x |  |  | x |  |  |  | 2 |
| National Agreement to Improve Product Composition: salt, saturated fat, sugar (calories) |  | x |  |  |  |  | x |  | 1 |
| Task Force for the Improvement of the Fatty Acid Composition |  | x |  |  |  | x |  |  | 2 |
| **New Zealand** | | | | | | | | | |
| Advertising restrictions |  | x |  |  |  |  | x |  | 1 |
| Advertising Standards Authority code |  | x |  |  |  | x |  |  | 4 |
| Healthy School Food Guidelines |  | x |  | x |  |  |  |  | 1 |
| Obesity strategy (Healthy Eating/Healthy Action) (under consultation) |  | x |  |  |  |  |  | x | 1 |
| Salt targets (different voluntary approaches) |  | x |  |  | x |  | x |  | 1 |
| Tick label |  | x |  |  | x |  |  |  | 2 |
| **Nigeria** | | | | | | | | | |
| Various diet-related policies |  | x |  |  |  |  |  | x | 1 |
| **Norway** | | | | | | | | | |
| Advertising restrictions |  | x |  |  |  |  |  | x | 1 |
| School Fruit Scheme |  | x |  |  | x |  |  |  | 1 |
| SSB and candy tax |  | x |  | x |  |  |  |  | 1 |
| **Peru** | | | | | | | | | |
| Advertising restrictions |  | x |  |  |  |  |  | x | 1 |
| Advertising controls |  | x |  |  |  | x |  |  | 1 |
| Trans-fat ban + labelling |  | x |  | x |  |  |  |  | 1 |
| Warning labels |  | x |  | x |  |  |  |  | 1 |
| **Philippines** | | | | | | | | | |
| Adopt-a-School’ Program |  | x |  |  |  |  | x |  | 1 |
| Responsible Advertising to Children Initiative (the ‘Philippines Pledge’) |  | x |  |  |  | x |  |  | 2 |
| Various Department of Education policy ‘Orders’ for schools |  | x |  | x |  |  |  |  | 1 |
| **Poland** | | | | | | | | | |
| Childcare food standards |  | x |  | x |  |  |  |  | 1 |
| Back-of-pack labelling |  | x |  |  | x |  |  |  | 1 |
| Federation of Food Producers Code on Advertising Food to Children |  | x |  |  |  | x |  |  | 1 |
| School Meal Reform |  | x |  | x |  |  |  |  | 1 |
| **Portugal** | | | | | | | | | |
| Portuguese Food Industry and Pledge on Diet, Physical Activity and Health: Advertising and Marketing Directed to Children |  | x |  |  |  | x |  |  | 1 |
| Salt reduction in products (voluntary by public sector) & labelling (mandatory) |  | x |  |  |  |  |  | x | 2 |
| SSB tax (mandatory) |  | x |  | x |  |  |  |  | 2 |
| **Puerto Rico** | | | | | | | | | |
| Trans-fat ban + labelling |  | x |  | x |  |  |  |  | 1 |
| **Republic of Korea** | | | | | | | | | |
| Advertising restrictions |  | x |  | x |  |  |  |  | 1 |
| Nutrient labeling |  | x |  | x |  |  |  |  | 1 |
| Salt reduction strategy- multicomponent |  | x |  |  | x |  |  |  | 1 |
| School food policies |  | x |  |  |  |  |  | x | 2 |
| Special Act on Safety Management of Children’s Dietary Life (advertising) |  | x |  | x |  |  |  |  | 3 |
| Trans-fat labelling |  | x |  | x |  |  |  |  | 1 |
| **Romania** | | | | | | | | | |
| Romanian Advertising Council |  | x |  |  |  | x |  |  | 1 |
| **Russia** | | | | | | | | | |
| The Russian Pledge ‘On Limitations on Advertising to Children’ |  | x |  |  |  | x |  |  | 1 |
| **Samoa** | | | | | | | | | |
| Salt reduction strategy |  | x |  |  |  |  |  | x | 1 |
| SSB tax |  | x |  | x |  |  |  |  | 2 |
| **Saudi Arabia** | | | | | | | | | |
| Menu energy-labelling |  | x |  | x |  |  |  |  | 1 |
| Sugary drink excise tax |  | x |  | x |  |  |  |  | 2 |
| **Serbia** | | | | | | | | | |
| The Law on Advertising |  | x |  | x |  |  |  |  | 1 |
| **Singapore** | | | | | | | | | |
| Singapore Code of Advertising Practice (SCAP) |  | x |  | x |  |  |  |  | 1 |
| Singapore Responsible Advertising to Children Pledge |  | x |  |  |  | x |  |  | 1 |
| **Slovenia** | | | | | | | | | |
| Advertising restrictions |  | x |  |  |  | x |  |  | 2 |
| Advertising restrictions |  | x |  |  |  |  |  | x | 1 |
| Salt reduction action plan |  | x |  |  | x |  |  |  | 1 |
| School Meals Act |  | x |  | x |  |  |  |  | 1 |
| Sugar reformulation |  | x |  |  |  | x |  |  | 1 |
| **South Africa** | | | | | | | | | |
| Advertising restrictions |  | x |  | x |  |  |  |  | 1 |
| Advertising restrictions |  | x |  |  |  | x |  |  | 1 |
| Back-of-pack labelling |  | x |  |  | x |  |  |  | 1 |
| Salt limits |  | x |  | x |  |  |  |  | 3 |
| School food policies inc National School Nutrition Programme |  | x |  |  | x |  |  |  | 1 |
| SSB excise tax |  | x |  | x |  |  |  |  | 3 |
| Health In Action |  | x |  |  |  |  | x |  | 1 |
| **Spain** | | | | | | | | | |
| Advertising restrictions |  | x |  |  |  |  |  | x | 1 |
| Advertising aimed at Children (PAOS) |  | x |  |  |  | x |  |  | 10 |
| Marketing/Advertising law |  | x |  | x |  |  |  |  | 1 |
| School food standards |  | x |  | x |  |  |  |  | 1 |
| SSB tax |  |  | x | x |  |  |  |  | 1 |
| **Sweden** | | | | | | | | | |
| Advertising restrictions |  | x |  |  |  |  |  | x | 1 |
| Back-of-pack labelling |  | x |  |  | x |  |  |  | 1 |
| School food standards & universal free school meals |  | x |  | x |  |  |  |  | 3 |
| **Switzerland** | | | | | | | | | |
| Trans fat limits |  | x |  | x |  |  |  |  | 2 |
| **Thailand** | | | | | | | | | |
| 25% SFS policy and logo |  | x |  |  |  |  | x |  | 1 |
| Marketing control of breast milk substitutes |  | x |  |  | x |  |  |  | 1 |
| Marketing to children |  | x |  |  |  | x |  |  | 1 |
| Radio and TV advertising regulation |  | x |  | x |  |  |  |  | 2 |
| SSB tax |  | x |  | x |  |  |  |  | 1 |
| Thailand Children’s Food and Beverage Advertising Initiative (Thai Pledge) |  | x |  |  |  | x |  |  | 1 |
| **Tonga** | | | | | | | | | |
| SSB tax (mandatory) |  | x |  | x |  |  |  |  | 1 |
| **Turkey** | | | | | | | | | |
| Advertising restrictions |  | x |  |  |  |  |  | x | 1 |
| Back-of-pack labelling |  | x |  |  | x |  |  |  | 1 |
| **United Arab Emirates** | | | | | | | | | |
| Back-of-pack labelling |  | x |  |  | x |  |  |  | 1 |
| **United Kingdom** | | | | | | | | | |
| Advertising restrictions before Ofcom (2005-2007) |  | x |  |  |  | x |  |  | 1 |
| Marketing control of breast milk substitutes |  | x |  | x |  |  |  |  | 1 |
| Guideline Daily Amounts label |  | x |  |  |  | x |  |  | 2 |
| Ofcom's television food advertising restrictions (from 2008) |  | x |  | x |  |  |  |  | 7 |
| Peas Please |  | x |  |  |  | x |  |  | 1 |
| Price increase in SSBs at Jamie's Italian restaurants |  | x |  |  |  | x |  |  | 1 |
| Public Health Responsibility Deal (England) |  |  | x |  |  |  | x |  | 12 |
| Salt Reduction Programme |  | x |  |  | x |  |  |  | 6 |
| Scottish Healthcare Retail Standard |  |  | x | x |  |  |  |  | 1 |
| School food & nutrient standards |  |  | x | x |  |  |  |  | 7 |
| Soft Drink Industry Levy (SDIL) |  | x |  | x |  |  |  |  | 14 |
| Supermarket checkout policies |  | x |  |  |  | x |  |  | 2 |
| Traffic Light Labelling |  | x |  |  | x |  |  |  | 1 |
| **United States of America** | | | | | | | | | |
| Various advertising control policies, incl. Children’s Food and Beverage Advertising Initiative (CFBAI) and Children’s Advertising Review Unit self-regulation (CARU) |  | x |  |  |  | x |  |  | 3 |
| Balance Calories Initiative |  | x |  |  |  | x |  |  | 1 |
| Child and Adult Care Food Programme (CACFP) |  | x |  |  | x |  |  |  | 9 |
| Children’s Confection Advertising Initiative |  | x |  |  |  | x |  |  | 2 |
| Children’s Food and Beverage Advertising Initiative (CFBAI) |  | x |  |  |  | x |  |  | 21 |
| Childcare centre nutrition standards (some mandatory, some voluntary) |  |  | x |  |  |  |  | x | 7 |
| Community Eligibility Provision (CEP) |  | x |  |  | x |  |  |  | 1 |
| Farm to school programs (some mandatory, some voluntary) |  |  | x |  |  |  |  | x | 1 |
| Fresh Fruit and Vegetable Program |  | x |  |  | x |  |  |  | 1 |
| Food and nutrition policy preemption laws |  |  | x | x |  |  |  |  | 1 |
| Food retail programmes (some mandatory, some voluntary/PPP): |  |  | x |  |  |  |  | x | 2 |
| Head Start |  | x |  |  | x |  |  |  | 2 |
| Healthy default beverages |  |  | x |  |  | x |  |  | 1 |
| Healthy Eating Standards for After School Program snacks |  | x |  |  | x |  |  |  | 2 |
| Healthier Food Initiative |  | x |  |  |  | x |  |  | 2 |
| Healthy foodservice guidelines |  | x |  |  | x |  |  |  | 1 |
| Healthy Weight Commitment Foundation Pledge |  | x |  |  |  | x |  |  | 2 |
| Kids LiveWell |  | x |  |  |  | x |  |  | 1 |
| Menu calorie labelling by companies (prior to mandatory in 2016) |  | x |  |  |  | x |  |  | 1 |
| Menu calorie labelling (including one study before the implementation deadline) |  | x |  | x |  |  |  |  | 7 |
| Menu labelling |  |  | x | x |  |  |  |  | 6 |
| New Markets Tax Credit |  | x |  |  | x |  |  |  | 1 |
| National School Lunch program |  | x |  | x |  |  |  |  | 4 |
| Nutrition labeling and Education Act |  | x |  | x |  |  |  |  | 3 |
| Nutrition, Physical Activity, and Obesity Program (NPAO) |  |  | x |  | x |  |  |  | 1 |
| Obesity programs |  |  | x | x |  |  |  |  | 1 |
| Reformulation-various (voluntary/self-regulation & voluntary by public or voluntary sector) |  | x |  |  | x | x |  |  | 1 |
| Restaurant tax |  |  | x | x |  |  |  |  | 1 |
| Salt reduction including public information/ education campaign; and on-package nutrition information |  | x |  |  | x |  |  |  | 1 |
| School advertising policies |  |  | x | x |  |  |  |  | 1 |
| School food policies incl. meals, vending machines & shops (some mandatory, some voluntary) |  |  | x |  |  |  |  | x | 37 |
| School beverage guidelines |  | x |  |  |  | x |  |  | 1 |
| School nutrition policies |  | x |  | x |  |  |  |  | 5 |
| School wellness policies (national/state/district) |  | x | x | x |  |  |  |  | 7 |
| Smart Choices (front-of-pack label) |  | x |  |  |  | x |  |  | 1 |
| Smarter Lunchrooms approach |  | x |  |  | x |  |  |  | 1 |
| Smart Snacks |  | x |  | x |  |  |  |  | 4 |
| SSB tax |  |  | x | x |  |  |  |  | 14 |
| Trans-fat labelling |  | x |  | x |  |  |  |  | 6 |
| Trans-fat labelling (national) and limits (state level) |  | x | x | x |  |  |  |  | 1 |
| Working Group on Food Marketed to Children |  | x |  |  |  | x |  |  | 1 |
| **Vietnam** | | | | | | | | | |
| Marketing control of breast milk substitutes |  | x |  | x |  |  |  |  | 2 |
| India: Shubh Aarambh |  | x |  |  |  |  | x |  | 1 |
| **Americas** | | | | | | | | | |
| The Americas: Trans-fat-free Americas (voluntary/pledge) | x |  |  |  |  | x |  |  | 1 |
| Central America: Central American Technical Regulation of Nutrition Labeling | x |  |  |  | x |  |  |  | 3 |
| Mercosur countries (Latin America): Trans fat labelling | x |  |  | x |  |  |  |  | 1 |
| **Canada & USA** | | | | | | | | | |
| Guiding Stars shelf labels | x |  |  |  |  | x |  |  | 1 |
| **European Union** | | | | | | | | | |
| Food labelling | x |  |  | x |  |  |  |  | 2 |
| EU Pledge | x |  |  |  |  | x |  |  | 5 |
| **Gulf countries** | | | | | | | | | |
| Gulf Cooperation Council Pledge | x |  |  |  |  | x |  |  | 1 |
| **Australia & New Zealand** | | | | | | | | | |
| Health Star Rating | x |  |  |  | x |  |  |  | 11 |
| **World** | | | | | | | | | |
| WHO: International Code of marketing of breastmilk substitutes | x |  |  |  | x |  |  |  | 5 |

# Supplementary Figure 1. Interactive evidence map.

Figure legend:

Supplementary Figure 1. Interactive evidence map. Number of publications by world bank region, country, policy type and governance approach.

This figure is presented in HTML format as a separate item.
