# Supplementary material for: Inequalities in Research on Food Environment Policies: An Evidence Map of Global Evidence from 2010-2020
Source: Adv Nutr. 2024 Sep 23;15(11):100306. doi: 10.1016/j.advnut.2024.100306 (PMC11555335; doi:10.1016/j.advnut.2024.100306)
Supplement: multimedia component 2 [file mmc2.zip › Suppl Fig1. Interactive map.html]

EPPI-Mapper


X

- Filters
- Hide Headers
  Show Headers
- Fullscreen
  Exit Fullscreen
- About
- Submit a Study
- View Records

Generated using v.2.2.4 of the EPPI-Mapper
powered by EPPI Reviewer
and created with


by the
Digital Solution Foundry team.
